# Age at release affects developmental physiology and sex-specific phenotypic diversity of hatchery steelhead trout (*Oncorhynchus mykiss)*

**DOI:** 10.1371/journal.pone.0315016

**Published:** 2025-02-13

**Authors:** Mollie A. Middleton, Donald A. Larsen, Christopher P. Tatara, Barry A. Berejikian, Chris R. Pasley, Jon T. Dickey, Penny Swanson

**Affiliations:** 1 School of Aquatic and Fisheries Science, University of Washington, Seattle, Washington, United States of America; 2 Environmental and Fisheries Sciences Division, Northwest Fisheries Science Center, National Marine Fisheries Service, National Oceanic and Atmospheric Administration, Seattle, Washington, United States of America; 3 Environmental and Fisheries Sciences Division, Northwest Fisheries Science Center, National Marine Fisheries Service, National Oceanic and Atmospheric Administration, Port Orchard, Washington, United States of America; 4 Winthrop National Fish Hatchery, United States Fish and Wildlife Service, Winthrop, Washington, United States of America; National Cheng Kung University, TAIWAN

## Abstract

Most steelhead trout hatcheries increase growth rate during rearing to produce and release yearling smolts for harvest augmentation, but natural steelhead exhibit variable age of smoltification, so this common rearing practice may not be ideal for programs focused on recovering imperiled wild stocks; therefore, it is important to investigate and compare alternative hatchery rearing methods that promote life history diversity. Over six consecutive years, the Winthrop National Fish Hatchery on the Methow River, WA reared and released paired groups of age-1 (S1) and age-2 (S2) steelhead smolts. To understand how the two rearing methods affected developmental ontogeny and life-history, fish were sampled prior to hatchery release for factors associated with smoltification (size, gill Na+/K+ ATPase activity, and a qualitative smolt phenotype) and sexual maturation (sex, pituitary and testis mRNA transcripts, gonadosomatic index, and plasma 11-ketotestosterone). Our objectives were to quantify levels of smoltification and male maturation during hatchery rearing, combine metrics to estimate residualism (failure to migrate upon release), and compare the treatments by sex. Overall, S2 rearing produced 7.8% more smolts and 44-fold (4.4 vs. 0.1%) more precociously mature males than S1 rearing. Conversely, S1 rearing produced 31.6% more residuals than S2 rearing. While the proportion of total male residuals was comparable between treatments, the S1 treatment produced approximately five-fold more female residuals (20.6 vs. 4.2%). Because residuals contribute minimally to adult returns and the number of returning adult females is critical to the success of salmonid supplementation efforts, developing rearing techniques that maximize migration in females is a management priority. Physiological assessments are useful for characterizing and quantifying the effects and risks of different hatchery rearing regimes on steelhead life-history, in addition to providing sex-specific guidance to inform and optimize conservation management goals in supplementation programs.

## Introduction

Natural steelhead (*Oncorhynchus mykiss*) populations exhibit, arguably, the most diverse life history among Pacific salmon species [1–3]. Anadromous steelhead and resident rainbow trout often inhabit the same river systems, have been shown to interbreed in tributaries [4,5] and produce offspring that follow the parental phenotype or the alternate life history pathway [6,7]. Natural *O*. *mykiss* populations display extensive variation in age (one to seven years [8]) of smoltification (seawater adaptation [9]) in preparation for outmigration and in size, timing and age at maturation (reviewed by Kendall et al. [3]). This life-history complexity is often referred to as a “portfolio effect” and may help maintain population viability in the face of stochastic environmental variation [2,10,11]. The ontogeny of development in *O*. *mykiss* is influenced by genetic and maternal factors [12–15] linked to size, growth rate, and whole-body lipid content during specific times of the year [16,17]. The influence of these factors varies according to the sex of the fish [15,18,19]. Furthermore, life history plasticity of *O*. *mykiss* is strongly influenced by environmental conditions, most notably water temperature, food availability, and competition, as well as the interaction between genotype and the environment [19–21].

In order to mitigate for stock declines resulting from loss of habitat, overharvest, and hydropower development, among other factors, steelhead hatchery programs have been implemented throughout the U.S. Pacific Northwest and Canada [22,23]. The primary goal of steelhead hatcheries is to increase the number of returning anadromous (as opposed to resident) adults for stock restoration efforts and to augment sport, tribal, and commercial fisheries. However, hatchery-reared steelhead often differ from naturally reared steelhead both physiologically and behaviorally [24–27], and these differences have been implicated in the reduced fitness of hatchery fish compared with wild fish when spawning in the natural environment [28,29]. One of the most profound differences between natural and hatchery origin steelhead is juvenile growth pattern [30]. Naturally reared *O*. *mykiss* typically spend two to three years in freshwater before smolting (designated as S2 or S3) and migrating to the ocean, where they spend one or more years in seawater before returning to their natal stream to spawn [8,31]. By contrast, for economy of rearing space, time, and expense, juvenile hatchery steelhead are typically reared to smolt at one year of age (designated as S1 [32]). While decreasing time in culture reduces opportunities for catastrophic events, such as a disease outbreaks and water supply or water quality issues, rearing in this manner may require intentional artificial selection for advanced spawn timing in hatchery broodstocks [33,34] and induce unintentional selection for rapid growth rate during hatchery rearing. Furthermore, accelerated developmental schedules in hatchery *O*. *mykiss* are associated with high numbers of fish that “residualize” in the freshwater environment and either delay migration to the ocean by one or more years, never migrate at all, or die (reviewed by Hausch and Melnychuk [35]) [36]. Extremes of body size are considered the primary determinants of residualism [36–38]. Males and females of small size may residualize as non-smolting and non-maturing parr, and delay migration or maturation until the subsequent year(s) if they do not die. Conversely, rapid growth rates may result in precocious male maturation at one or two years of age with these fish remaining in freshwater to attempt spawning naturally with resident trout or anadromous females. While residual hatchery steelhead could be viewed as individuals representing segments of the natural steelhead life history portfolio [2], hatcheries can produce unnaturally large numbers of residuals which may increase rates of interbreeding and competition with natural populations, reduce anadromy, and complicate management of natural populations [36,39–44].

Because most juvenile steelhead appear immature at release and would not exhibit external signs of maturation for another year, the most effective way to describe sex-specific life history employs a combination of methods including body size measurements, smolt characteristics, and physiological markers of maturation. Smoltification is commonly characterized by examining visual changes in body morphology and pigmentation, as well as physiological measures of increased salinity tolerance such as Na+/K+ ATPase activity in gill tissue [45]. Increases in gill ATPase activity generally occur seasonally in freshwater, prior to seawater entry, and are associated with increased salinity tolerance and seaward migration [45,46]. Both male and female steelhead initiate maturation with minimal visible changes in phenotype (external secondary sex characteristics) up to one year in advance of spawning [47–49]. Unlike males, precocious maturation in females is rare since gonad development is more energetically “expensive” for females [50] and they typically initiate maturation either during or after smoltification associated with a change in salinity [51]. As a result, most previous work has been focused on male steelhead life-history and more specifically male precocious maturation.

Onset of maturation in vertebrates, including teleosts, is regulated by the brain-pituitary-gonad axis, thus, the endocrine factors in this pathway can be quantified and used as a suite of predictive biomarkers to characterize sexual maturation. In males, markers include protein hormones, such as follicle stimulating hormone (FSH), luteinizing hormone (LH), anti-Mullerian hormone (AMH), and insulin-like growth factor-3 (IGF3), and evaluating mRNA encoding for these proteins allows for earlier insight into maturation progression because it occurs prior to hormone production [48,49,52]. In brief, pituitary FSH has a regulatory effect on initiating spermatogenesis through a balance of inhibitory and stimulatory signals for spermatogonial proliferation from testicular AMH [53] and IGF3 [54], respectively. FSH simultaneously stimulates the production of spermatogenesis-inducing steroids, including plasma 11-ketotestosterone (11KT), which increase gradually during spermatogenesis (reviewed by Schulz et al. [52]). Finally, gonadosomatic index (GSI) begins to increase rapidly during the meiotic phase of spermatogenesis [55] concurrent with the first detectable levels of LH [52]. Collectively, previous work in age-1 hatchery, winter-run steelhead demonstrated that, in May approximately one year prior to the next annual spawning period, males initiating maturation had elevated expression of mRNA in the pituitary (*fshb* and *lhb*) and testis (*igf3*), reduced expression of testis *amh* mRNA, and higher GSI and plasma 11KT than their immature counterparts [49].

In the current investigation, we focused on examining the life-history composition of S1 and S2 reared summer-run steelhead at the Winthrop National Fish Hatchery (WNFH) on the Methow River in north-central Washington, USA. Steelhead hatchery programs that are tasked with recovery of stocks listed as threatened or endangered under the United States Endangered Species Act (Federal Register 70:37160) are mandated to use natural-origin broodstock that exhibit natural spawn timing [56]. Later spawn timing in natural-origin broodstock compared to hatchery-origin broodstock compels such programs to develop rearing methods that accommodate lost growth opportunity by producing age-2 smolts. The WNFH is one of the first programs to systematically examine the efficacy of S2 rearing (see also Bjornn and Ringe [57]). Historically, WNFH released only S1 steelhead smolts produced from hatchery-origin broodstock, but in release year 2010, the hatchery implemented a transition to using only natural-origin broodstock and releasing S2 steelhead smolts [56,58]. To hedge against the uncertainty associated with a full transition from S1 to S2 rearing, the WNFH conducted paired releases of both S1 and S2 steelhead for six consecutive years. Previous studies at WNFH highlighted the post-release similarities and differences in survival and migratory behavior between fish reared under these two regimes [36,58]. Post-release assessments can underestimate smoltification, maturation, and residualism due to sampling method and/or mortalities that occur prior to sampling or detection. This study consists of a quantitative physiological assessment of fish from both sexes in the S1 and S2 treatments conducted prior to release, as that may provide useful insights for improving our understanding of the observed differences in post-release performance and help guide steelhead supplementation programs in the future.

Our multi-year analysis of data including both sexes from two different ages at release in a summer-run population is novel. In five out of six release years, we sampled approximately 600 representative S1 and S2 reared fish for a suite of morphological and physiological measures associated with smoltification and sexual development in order to characterize potential residuals. We had three primary objectives: 1) characterize the sex-specific levels of a) smoltification and b) male maturation for both rearing treatments; 2) combine both morphological and physiological measures to predict sex-specific residualism for both rearing treatments; and 3) discuss the utility of individual measurements for predicting residual proportions within a hatchery setting in a cost-benefit framework. Based on our previous studies we developed the following working hypotheses: S2 rearing would produce more smolts and more precocious male maturation, but lower residualism overall compared to S1 rearing. Further, we hypothesized that S2 rearing would produce a higher proportion of male residuals than S1 rearing.

## Materials and methods

### Fish rearing conditions

**Fig 1:**
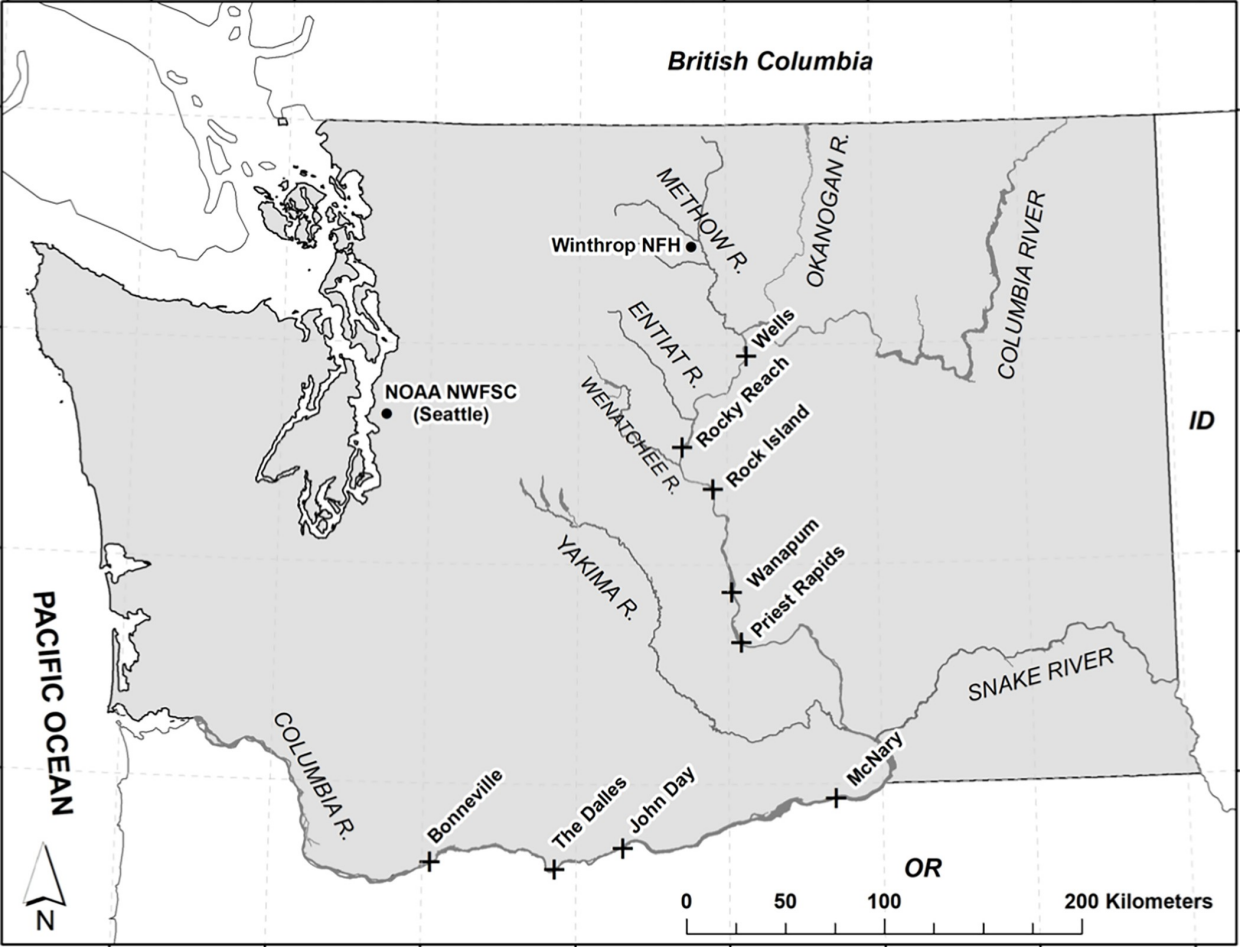
Map of Washington State. Location of the Winthrop National Fish Hatchery (NFH) and the NOAA Northwest Fisheries Science Center (NWFSC) laboratory in Washington State. Dams that fish pass through during migration are marked with an “X”, including Wells Dams (adjacent to Wells Hatchery). Tributary rivers for the migration route are also labeled. Figure created using USGS National Hydrography Dataset (NHD) data.

Broodstock were collected between 2010 and 2014 for the S1 release group and between 2009 and 2013 for the S2 release group. S1 broodstock were collected and spawned from fish returning to the collection facility at the Douglas County Public Utility District operated Wells Hatchery located at the base of Wells Dam in all years (47°56’49.2”N 119°52’14.16”W) (Fig 1). S2 broodstock were collected by angling in the Methow River in all years and were spawned at the United States Fish and Wildlife Service (USFWS) operated Winthrop National Fish Hatchery (WNFH) (48°28’30.147”N 120°11’16.1766”W) in north central Washington State, USA (Fig 1). Steelhead embryos for the S1 release were transferred to the WNFH at the eyed stage of development for rearing after a safe number of incubation degree days were obtained. Fish were reared to a target size at release, regardless of age class, with the goal of minimizing pronounced size differences, as determined by the WNFH’s management plan [56]. Detailed information on broodstock, rearing, and environmental conditions in release year (RY) 2011–2014 were previously reported by Tatara et al. [36,58] and USFWS [56]. Conditions were similar among years, including RY 2015 which is unreported.

### Fish sampling procedure

In each release year, steelhead from both rearing treatments were sampled during the last week of March, approximately one month prior to release from the hatchery. During each sampling event, approximately 600 fish from each release group were randomly netted from outdoor raceways. Fish were held in indoor raceways for no longer than 24 hours prior to sampling in order to minimize overall effects of multiple netting events. All fish were sampled according to the University of Washington Institutional Animal Care and Use Committee (Protocol #2313–90).

Fish were anesthetized with buffered 0.05% tricaine methanesulfonate (MS-222, Argent Chemical Laboratories, Redmond, WA) and measured for fork length (mm) and weighed (g) to the nearest 0.1 g. Smoltification was visually assessed based on phenotypic characteristics and fish were assigned a smolt phenotype of either parr, transitional, or smolt (modified from Gorbman et al. [59], see Tatara et al. [36] for visual reference). Precociously mature males were characterized based on expression of milt. Blood samples were collected from the caudal vasculature using pre-heparinized Natelson tubes (Fisher Scientific, Hampton, NH) and stored in 0.4 ml polypropylene tubes on ice until sex could be determined macroscopically via dissection of the gonad. Male blood samples were centrifuged at 3000 x g for 6 minutes and female blood samples were discarded. Plasma was removed and frozen on dry ice.

Gill filaments, including approximately 2 mm of supporting arch, were dissected, preserved in cold SEI buffer (250 mM sucrose, 10 mM Na_2_EDTA, and 50 mM imidazole [60]), and frozen on dry ice. Male pituitary glands were individually dissected and preserved in 0.5 ml RNA*later* (Ambion, Carlsbad, CA) for analysis of gonadotropin beta (*b*) subunit mRNAs. Paired testes were removed and weighed to the nearest 0.001 g. Gonadosomatic index (GSI) was calculated according to the following equation: $\frac{\mathrm{gonad}\;\mathrm{weight}\;(g)}{\mathrm{body}\;\mathrm{weight}\;(g)}\times 100$. In RY 2011 and 2012 only, one testis was preserved in 0.75 ml RNA*later* for analysis of mRNAs associated with spermatogenesis. In all years, a single testis was placed in 1 ml of fixative for histological analysis (See S1 File for histological methods and results).

Plasma and tissue samples were transported to the National Oceanic and Atmospheric Administration (NOAA), Northwest Fisheries Science Center, Seattle, WA for analysis. Plasma and gill samples were stored at -80°C. Tissues preserved in RNA*later* remained at room temperature for seven days before the solution was removed and tissues were stored at -80°C.

### Gill Na+/K+ ATPase activity

In RY 2012–2015, 200 gill samples were collected from each treatment regardless of sex. Gill samples were not collected in RY 2011. Gill Na+/K+ ATPase activity, reported in units of μmol ADP · mg protein^-1^ · hr^-1^, was measured according to previously described methods [61].

### Tissue mRNA analyses

Total RNA for pituitary and testis samples was isolated using Qiagen’s RNeasy Plus spin column kit (Qiagen, Valencia, CA) according to the manufacturer’s instructions. Integrity of the RNA was verified by an optical density (OD) absorption ratio OD 260 nm/OD 280 nm >1.9 and quantified by spectrophotometry at 260 nm using a NanoDrop ND-1000 (NanoDrop Technologies, Wilmington, DE). Total RNA was diluted with nuclease-free water (BioExpress, Radnor, PA) to 10.0 ng/μl and 30 ng per sample was reverse transcribed with Superscript II RNase H-reverse transcriptase (Invitrogen, Carlsbad, CA). Reverse transcription (RT) reaction conditions were as follows: 3.0 μl of 5X buffer, 1.5 μl of 0.1 M dTT, 0.75 μl of dNTPs (stock of 10 mM each dCTP, dGTP, dTTP and dATP; Promega, Madison WI), 0.225 μl random hexamers (500 ng/μl stock; Promega), 0.1875 μl Superscript II (200 U/μl; Invitrogen), 0.3 μl RNase inhibitor (20 U/μl; Promega), 6.0375 μl nuclease-free water (BioExpress) and 3.0 μl of template. RT reactions were conducted in a BioRad C1000 thermal cycler (BioRad, Hercules, CA) with the following temperature profile: 23°C for 10 min, 48°C for 60 min and 95°C for 10 min, followed by a 4°C incubation. Samples were diluted 1:1 with nuclease-free water (10 μl cDNA + 10 μl water) prior to measurement in real-time quantitative polymerase chain reaction (qPCR) assays.

Primers and probes for real-time RT-qPCR assays were designed according to sequence data using Primer Express 1.5 software (Applied Biosystems (ABI), Foster City, CA) and purchased from Integrated DNA Technologies (San Jose, CA). When possible, intron/exon splice junctions were used in primer design to avoid potential signal from contaminating genomic DNA. Primer and probe sequences for follicle stimulating hormone beta-subunit (*fshb*), luteinizing hormone beta-subunit (*lhb*), anti-Mullerian hormone (*amh*), insulin-like growth factor-3 (*igf3*), and a reference gene, elongation factor one alpha (*ef1a*), were previously reported by Middleton et al. [49]. For each transcript measured, eight randomly selected total RNA samples from each treatment were analyzed without RT reaction to test for amplification due to genomic DNA contamination (no amplification control, NAC). NAC samples showed no, or negligible, DNA contamination with all samples amplifying >7 cycles (<1% DNA contribution) beyond those of their reverse transcribed counterparts. No template controls (NTC), consisting of an RT reaction containing no RNA template, were also performed and showed no template contamination in the reagents used.

All assays were run on an ABI 7900 HT Fast Real-Time PCR System using 384-well plates and TaqMan Universal PCR Master Mix (ABI). PCR efficiency for each transcript was measured using a serial dilution of six pooled testis or pituitary RNA samples from within each RY as a reference standard. Thirteen standard curve dilutions were run in triplicate, samples were run in singles. In previous studies we found that the greatest source of variation was biological rather than technical [48,49] so we opted to run large sample numbers (approximately 300 males per treatment). Samples of the same tissue type within a RY (combining both treatments) were randomized and distributed evenly across two plates. In order to normalize between the two plates, three samples from each treatment, for a total of six samples, were combined to create an inter-plate pool. Twelve replicates of the inter-plate pool were run on each plate and results were adjusted so the average Ct values from each plate demonstrated a difference less than 1%. Reaction conditions were as follows for 12 μl PCRs: 6.0 μl of TaqMan Universal PCR MasterMix (ABI), 0.22 μl of forward primer (45 μM stock), 0.22 μl of reverse primer (45 μM stock), 0.24 μl of probe (10 μM stock), 2.32 μl nuclease-free water (BioExpress) and 3.0 μl of diluted RT reaction. Cycling parameters were: 50°C for 2 min, 95°C for 10 min and 45 cycles of 95°C for 15 s followed by 60°C for 1 min.

Stable expression of the reference gene *ef1a* was observed within each tissue type (data not shown). Therefore, transcript levels were calculated using the serially diluted total RNA sample standard curve and efficiency corrected by *ef1a* using the method described by Pierce et al. [62]. To avoid interpretation of the data being driven by a single spurious low sample, relative expression values for all mRNAs within a tissue type and RY were calculated based on the method described by Middleton et al. [49].

### Plasma 11-Ketotestosterone (11KT)

Plasma was heat treated prior to assay similar to the method of Schulz et al. [63]. Each sample was diluted 1:2 with sterile water (50 μl plasma + 100 μl water) and heated in a water bath at 80°C for one hour. Samples were then centrifuged at 18,000 x g for 6 min and supernatants were stored at -20°C until assay. Plasma 11KT (ng/ml) was measured by enzyme-linked immunosorbent assay (ELISA) similar to the method previously described by Cuisset et al. [64]. Acetylcholinesterase tracer and pre-coated (mouse anti-rabbit IgG) 96-well plates were purchased from Cayman Chemical (Ann Arbor, MI). Primary antibody was provided by Dr. David Kime (University of Sheffield, UK, retired).

### Statistical analyses

Data were tested for normality using the D’Agostino-Pearson omnibus *K*^2^ normality test. To control for heteroscedasticity, data for pituitary *fshb* and *lhb*, testis *amh* and *igf3*, GSI, and plasma 11KT were all log_10_ transformed prior to statistical analyses. A finite mixture model (fmm) was used to analyze frequency distributions of fork length and log_10_ transformed *fshb*, *lhb*, *amh*, *igf3*, GSI, and plasma 11KT data with the purpose of obtaining defined “threshold values” between modes. Based on the fmm analysis, “threshold values” were determined for each parameter at mode intersection points for the best fit models as determined by Bayesian information criterion (BIC) values using previously validated methods [49]. For more details on how modes are determined and their biological significance, see Middleton et al. [49].

Threshold values from the fmm analysis were then used to classify males as “immature” or “maturing” for all parameters except fork length, which was not used as a determining factor of maturation status and instead used exclusively for classifying residualism. Classification as a “maturing” male indicated that the maturation process had initiated but was unlikely to be completed prior to release. Classification as “maturing” was based on exceeding the lowest threshold value (i.e. inclusion in the middle or upper modes) or, in the case of *amh* failing to exceed the lowest threshold value (i.e. inclusion in the lower mode), for at least one of the six maturation parameters analyzed. Males were categorized as “immature” if they failed to exceed the lowest threshold value (i.e. inclusion in the lower mode), except for *amh* where males exceeding the lowest threshold value (i.e. inclusion in the upper mode) were categorized as “immature”. In most cases, fish classified as “maturing” exceeded threshold values for more than one maturation parameter. As detailed above, fish identified as producing milt at the time of sampling were classified as “mature”, having both initiated and completed the maturation process prior to sampling. Differences between “immature” and “maturing” fish required identification by physiological measurement and were the primary focus of our statistical hypotheses. Because precociously mature males are a visually identifiable phenotype that does not require categorization using physiological markers, we recognized them as biologically significant outliers. In lieu of including mature males in statistical analyses, we chose to utilize data for mature males as a demonstration of positive control for the physiological tools used. Additionally, mature males were examined as an indicator of fmm analysis validity and maturation status classification methods; i.e. in cases where the best fit was trimodal and values from mature males were available for analysis, mature males were contained to the upper mode resulting in classification as mature. We were therefore able to use a positive control confirmation method to determine that our physiological and statistical methods were congruent with visual maturity determinations at the time of sampling and thereby lend greater credibility to our statistical classifications of “immature” and “maturing”.

Fish were further categorized as either a “migrant” or “residual” according to the following sex-specific criteria. Females were categorized as “residual” based solely on failure to exceed the fork length threshold value (i.e. inclusion in the lower mode) obtained from the fmm analysis; no females exhibited signs of maturation, such as enlarged oocytes, at the time of sampling and as such all females were visually categorized as “immature”. Males were categorized as “residual” based on a combination of exceeding maturation metric threshold values (i.e. inclusion in the middle or upper modes) and failure to exceed fork length threshold values (i.e. inclusion in the lower mode). All fish not meeting the sex-specific criteria as described above were categorized as a “migrant”.

We chose to pool samples across years in order to include reasonable environmental differences in rearing conditions among release years (See S2 File for data from individual release years). Pooling samples served two purposes: 1) ensuring that the physiological tools and statistical results are robust to minor yearly environmental variation typical at hatcheries and 2) acknowledging the expectation that the age-at-release effect should be larger than any environmental effects. All data were graphed using Prism 7 software (GraphPad Software, La Jolla, CA). Statistical analyses were performed in R (version 4.0.2, R Foundation, Vienna, Austria). Fmm analyses were performed with the mixtools package (version 1.2.0 [65]). Two-way analysis of variance (ANOVA) and Tukey post-hoc tests were performed with the car package (version 3.0–8 [66]) and agricolae (version 1.3–3 [67]) packages, respectively. Two sample t-tests of S1 vs. S2 fish within a maturation category were performed with the built-in t.test function. Because mature males are biologically significant outliers, they were censored from both ANOVA and t-test analyses. Pearson’s chi-square tests for equality of proportions were performed to compare rearing treatments, sex, smolt phenotypes, male maturation, and residualism. The level of significance for all statistical analyses was set at α = 0.05, with adjustments for multiple testing performed by the applicable R packages.

## Results

The majority of fmm analyses resulted in a best model fit with either two or three modes, the exceptions being S1 *fshb* and *amh*, which were best fit with a single mode. BIC values and mode intersections (hereafter referred to as threshold values) are presented in Table 1.

**Table 1:** Results from finite mixture model (fmm) analysis for morphological and physiological characteristics used to assess maturation status and predicted residualism of age-1 (S1) and age-2 (S2) steelhead.

Treatment	Group	Measure	BIC fmm1	BIC fmm2	BIC fmm3	Best model fit	Threshold value 1	Threshold value 2
**S1**	**All Fish**	**Fork Length**	29264.26	**28521.88**	28540.37	fmm 2	146.6	NA
	**Male**	***fshb***	**1874.45**	1893.00	1905.08	fmm 1	NA	NA
		***lhb***	2860.86	**2420.25**	2422.39	fmm 2	44.7	NA
		***amh***	**-545.83**	-538.99	-533.06	fmm 1	NA	NA
		***igf3***	367.67	217.59	**207.63**	fmm 3	6.2	14.8
		**GSI**	-577.80	-1073.89	**-1138.83**	fmm 3	0.051	0.627
		**plasma 11KT**	223.40	15.29	**-11.11**	fmm 3	0.88	23.08
**S2**	**All Fish**	**Fork Length**	27354.16	**27066.63**	27067.72	fmm 2	147.6	NA
	**Male**	***fshb***	2900.81	**2638.44**	2645.39	fmm 2	270.0	NA
		***lhb***	4534.94	3433.19	**3147.48**	fmm 3	94.9	11306.5
		***amh***	-537.16	**-538.95**	-524.68	fmm 2	2.7	NA
		***igf3***	688.40	455.40	**429.29**	fmm 3	6.2	16.6
		**GSI**	2287.49	-546.14	**-881.53**	fmm 3	0.056	0.962
		**plasma 11KT**	2815.37	1408.64	**1174.14**	fmm 3	0.97	15.81

Best fit modes from fmm analysis for juvenile steelhead sampled at Winthrop National Fish Hatchery in release years 2011–2015 combined. Lowest Bayesian information criterion (BIC) value is indicated in bold. Threshold values 1 and 2 indicate the metric value at which modes intersect. For bimodal distributions, the lower and upper modes intersect at threshold value 1 and there is no threshold value 2. For trimodal distributions, the lower and middle modes intersect at threshold value 1 and the middle and upper modes intersect at threshold value 2. Abbreviations and units of measure are as follows: fork length (mm); pituitary follicle stimulating hormone beta-subunit (*fshb*) and luteinizing hormone beta-subunit (*lhb*), testis anti-Mullerian hormone (*amh*) and insulin-like growth factor-3 (*igf3*) (relative mRNA expression); gonadosomatic index (GSI; %); plasma 11-ketotestosterone (11KT; ng/ml).

### Objective 1a –Sex-specific smoltification by rearing treatment (fish size, gill ATPase activity, and smoltification phenotypes)

**Fig 2:**
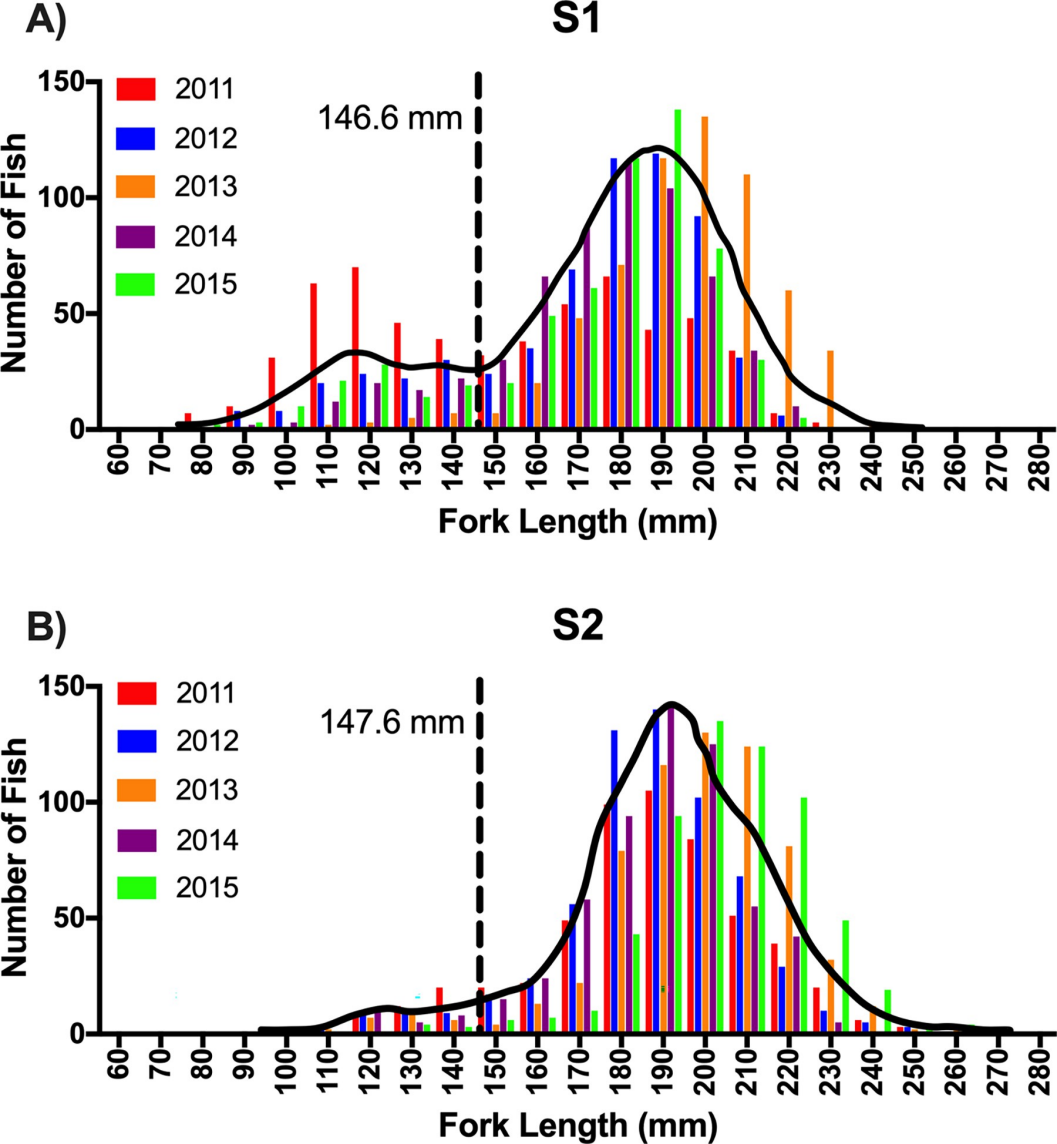
Frequency distribution–fork length. Frequency distributions of S1 (A) and S2 (B) fork length (mm) for juvenile steelhead sampled at Winthrop National Fish Hatchery in release years 2011–2015. Interleaved colored bars represent individual release years. Solid black lines indicate the density distribution for pooled release years. Dashed black reference lines indicate mode intersection as determined by finite mixture model analysis and establish the threshold value for predicted post-release status. Fish with fork lengths less than the reference line value (lower mode) were classified as residuals and those with fork lengths greater than the reference line value (upper mode) were classified as migrants.

**Fig 3:**
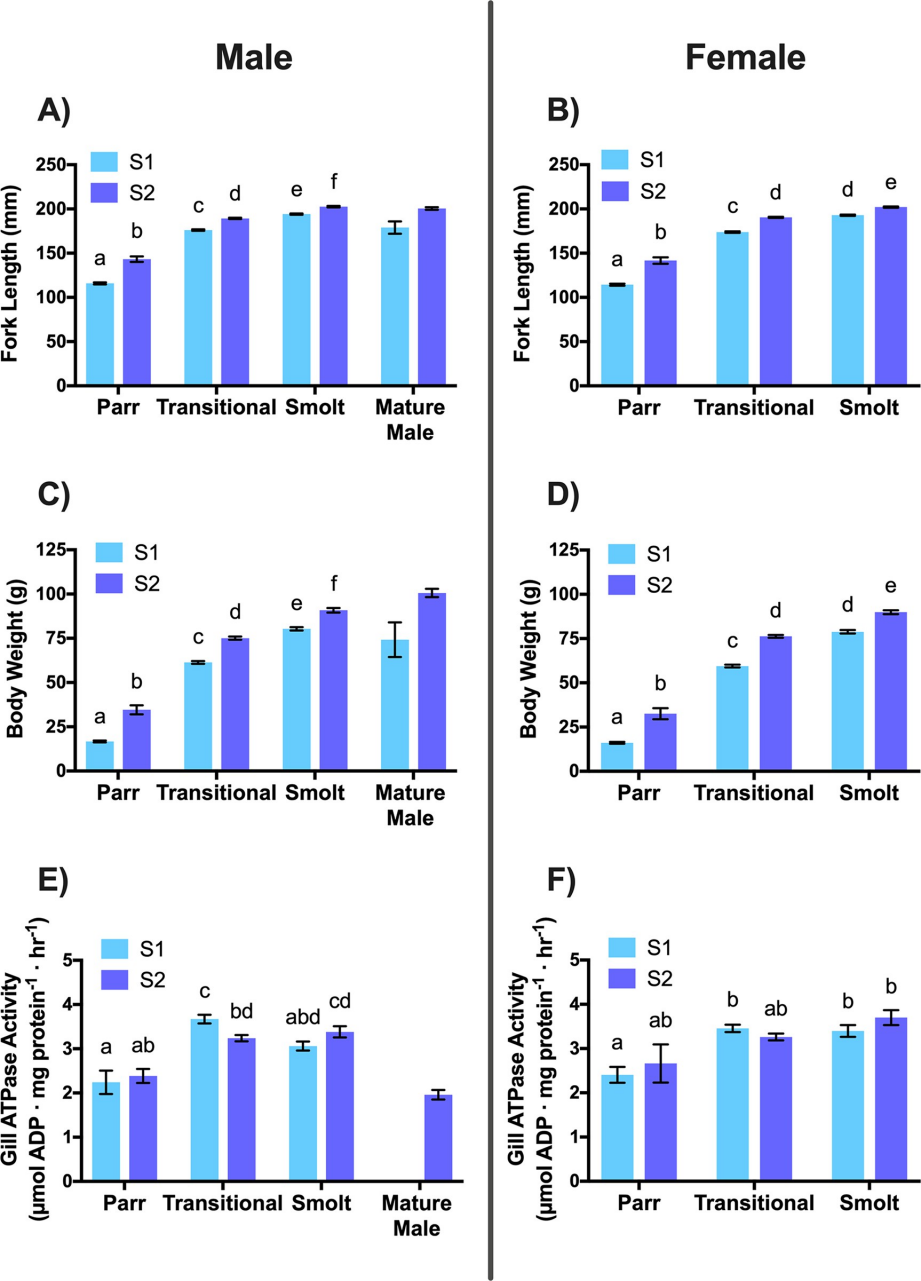
Male and female fork length, body weight, and gill ATPase activity by rearing treatment and smolt phenotype. Fork length (mm; A-B), body weight (g; C-D), and gill Na+/K+ ATPase activity (μmol ADP · mg protein^-1^ · hr^-1^; E-F) of juvenile steelhead sampled at Winthrop National Fish Hatchery in release years 2011–2015 combined. Data are separated according to rearing treatment (S1 in light blue, S2 in violet) and visually determined qualitative smolt phenotype for males (A, C, E) and females (B, D, F). Data are mean ± SEM. Different letters indicate significant differences (p < 0.05) as determined by two-way ANOVA with Tukey’s post-hoc test. Mature males were not included in statistical analyses but are included on the graphs for visual reference.

Frequency distributions for both S1 and S2 fork lengths were bimodal with similar threshold values of 146.6 and 147.6 mm, respectively (Fig 2; Table 1). Two-way ANOVA analyses revealed statistically significant interactions between the effects of treatment and smolt phenotype on fork length and body weight for both sexes (Table 2). Males and females within each rearing treatment increased in fork length (Fig 3A and 3B) and body weight (Fig 3C and 3D) from the parr to transitional to smolt phenotype (as identified visually). Although excluded from statistical analysis, S1 mature males were similar in fork length (179.0 ± 12.1 mm, mean ± SD) and body weight (74.20 ± 16.97 g, mean ± SD) to the S1 male transitional phenotype (fork length: 176.1 ± 23.2 mm, body weight: 61.38 ± 22.58 g; mean ± SD) (Fig 3A and 3C). S2 mature males were similar in fork length (200.5 ± 17.4 mm, mean ± SD) and body weight (100.60 ± 26.73 g, mean ± SD) to the S2 male smolt phenotype (fork length: 202.6 ± 18.5 mm, body weight: 90.84 ± 27.67 g; mean ± SD) (Fig 3A and 3C). S1 fork length and body weight were less than that of S2 fish within each smolt phenotype (Fig 3A–3D) regardless of sex.

**Table 2:** Two-way ANOVA analysis results for size and gill ATPase activity by sex (M, F) comparing rearing treatment (S1, S2), visually determined qualitative smolt phenotype (parr, transitional, smolt), and their interaction.

Sex	Measure	Source of variation	Sum of Squares	df	F-value	p-value
**Male**	**Fork Length**	**Treatment (S1/S2)**	111478	1	263.562	***
		**Smolt Phenotype**	1029557	2	1217.065	***
		Treatment * Smolt Phenotype	15154	2	17.914	***
	**Body Weight**	**Treatment (S1/S2)**	116301	1	216.6902	***
		**Smolt Phenotype**	752105	2	700.6563	***
		Treatment * Smolt Phenotype	2893	2	2.6952	.
	**gill ATPase activity**	**Treatment (S1/S2)**	5.60	1	3.5714	.
		**Smolt Phenotype**	52.78	2	16.8189	***
		Treatment * Smolt Phenotype	21.90	2	6.9792	***
**Female**	**Fork Length**	**Treatment (S1/S2)**	159408	1	376.254	***
		**Smolt Phenotype**	963686	2	1137.301	***
		Treatment * Smolt Phenotype	15251	2	17.999	***
	**Body Weight**	**Treatment (S1/S2)**	161440	1	315.647	***
		**Smolt Phenotype**	675531	2	660.399	***
		Treatment * Smolt Phenotype	5291	2	5.172	**
	**gill ATPase activity**	**Treatment (S1/S2)**	0.24	1	0.1388	
		**Smolt Phenotype**	28.56	2	8.1013	***
		Treatment * Smolt Phenotype	9.65	2	2.7359	.

Results from two-way ANOVA analysis of juvenile steelhead sampled at Winthrop National Fish Hatchery in release years 2011–2015 combined for size metrics and gill Na+/K+ ATPase activity with rearing treatment (S1, S2), visually determined qualitative smolt phenotype (parr, transitional, smolt), and their interaction as factors. Separate analyses were conducted for males and females. Asterisks indicate p-value significance < 0.05, symbol meanings are as follows

“***”, p-value < 0.001; “**”, p-value between 0.001 and 0.01; “.”, p-value between 0.05 and 0.1. Units of measure are as follows: fork length (mm); body weight (g); gill Na+/K+ ATPase activity (μmol ADP · mg protein^-1^ · hr^-1^).

Two-way ANOVA analyses revealed a statistically significant interaction between the effects of treatment and smolt phenotype on gill ATPase activity for males, but not for females (Table 2). Males and females within each rearing treatment demonstrated differing gill ATPase activity from parr to transitional to smolt phenotypes (as identified visually) (Fig 3E and 3F). S1 males demonstrated an increase in gill ATPase activity from the parr to transitional phenotype, but there was a decrease in gill ATPase activity from the transitional to smolt phenotype (Fig 3E). S1 females also demonstrated an increase in gill ATPase activity from the parr to transitional phenotype, but comparable gill ATPase activity in the transitional and smolt phenotypes (Fig 3F). The S2 rearing treatment displayed similar gill ATPase activity among smolt phenotypes for both sexes; however, male S2 smolts had higher gill ATPase activity than male S2 parr (Fig 3E and 3F). For both sexes, gill ATPase activity was similar between S1 and S2 fish within each visually identified smolt phenotype, except for the male transitional phenotype where S1 gill ATPase was greater than S2 (Fig 3E and 3F).

**Fig 4:**
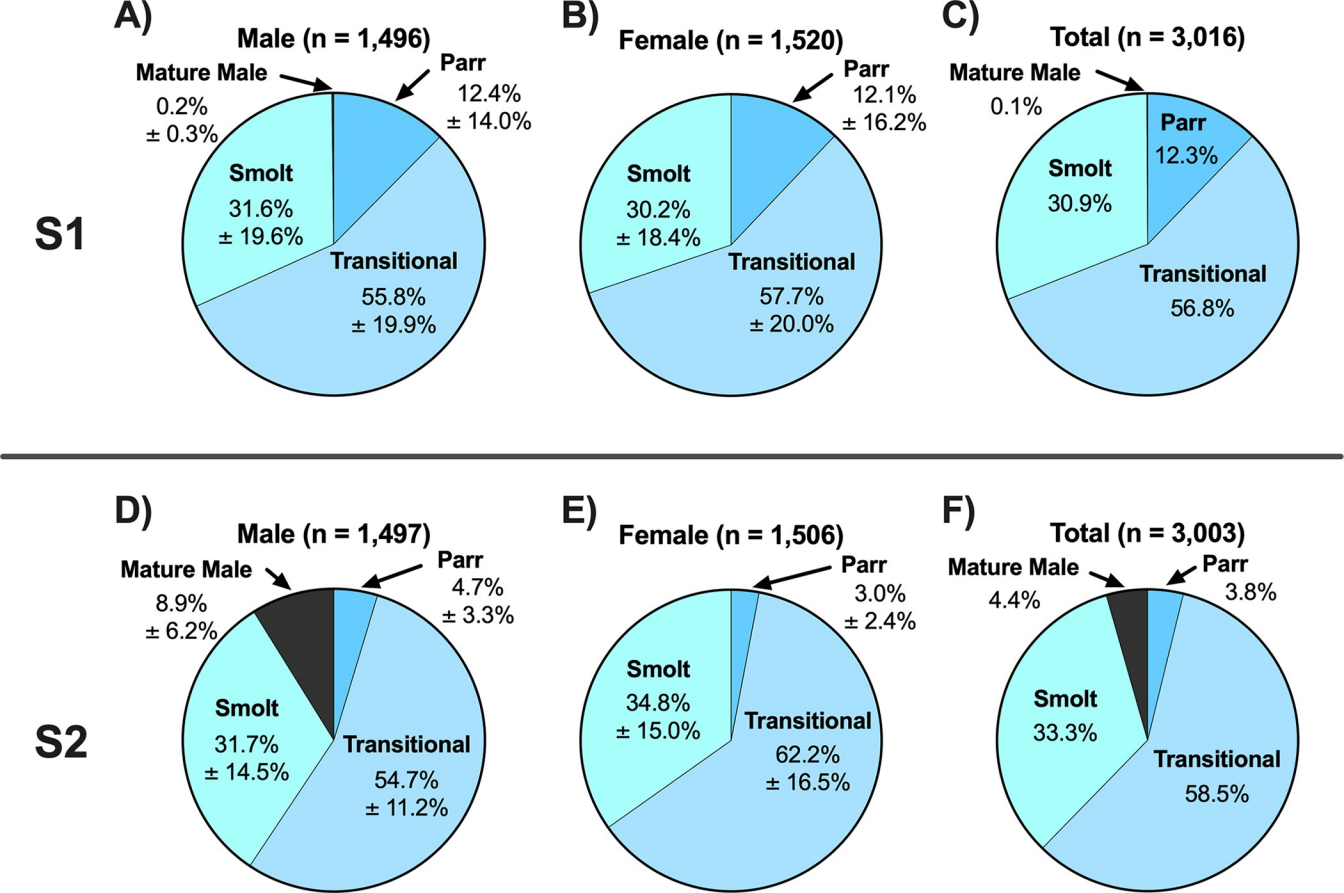
Proportion of smolt phenotype by rearing treatment and sex. Proportion of juvenile steelhead sampled at Winthrop National Fish Hatchery in release years 2011–2015 for each visually determined qualitative smolt phenotype (parr, transitional, smolt, mature male) separated according to rearing treatment (S1: A-C, S2: D-F) and sex (males: A, D; females: B, E; sexes combined: C, F). Proportions for males and females are represented as mean ± SD of individual release years. No SD is provided for the total (sexes combined) charts (C, F) because samples were pooled from all release years.

When each rearing treatment was separated by sex, S1 males and females displayed similar proportions of parr, transitional, and smolt phenotypes and very few (3 out of 1,496) mature males (Fig 4A and 4B; Table 3A). The S2 males and females also showed similar proportions of smolt phenotypes with some exceptions; there were more male than female parr and fewer males than females of the transitional phenotype (Fig 4D and 4E; Table 3B). The S1 rearing treatment produced a smaller proportion of mature males compared to the S2 rearing treatment (133 out of 1,497) (Fig 4A and 4D; Table 3C). After combining both sexes, proportions of the transitional phenotype were comparable between the S1 and S2 rearing treatments; however, there were more parr in the S1 treatment than the S2 treatment (S1: 370 out of 3,016; 12.3% vs. S2: 115 out of 3,003; 3.8%) and less smolts in the S1 than the S2 treatment (S1: 931 out of 3,016; 30.9% vs. S2: 999 out of 3,003; 33.3%) (Fig 4C and 4F; Table 3D).

**Table 3:** Pearson’s chi-square test for equality of proportions comparing rearing treatment (S1, S2), sex (M, F), visually determined smolt phenotype (parr, transitional, smolt), maturation status (immature, maturing, mature), and predicted residuals.

Test	Reference	*X* ^2^	df	N	p-value	Result
S1 male parr vs. S1 female parr	A	0.075	1	3016	0.7838	NS
S1 male transitional vs. S1 female transitional	A	1.088	1	3016	0.2969	NS
S1 male smolt vs. S1 female smolt	A	0.647	1	3016	0.4211	NS
S2 male parr vs. S2 female parr	B	5.808	1	3003	0.0080	M > F
S2 male transitional vs. S2 female transitional	B	17.430	1	3003	< 0.001	M < F
S2 male smolt vs. S2 female smolt	B	3.175	1	3003	0.0748	NS
S1 mature male vs. S2 mature male	C	125.760	1	6019	< 0.001	S1 < S2
S1 parr vs. S2 parr	D	144.630	1	6019	< 0.001	S1 > S2
S1 transitional vs. S2 transitional	D	1.804	1	6019	0.1793	NS
S1 smolt vs. S2 smolt	D	3.972	1	6019	0.0231	S1 < S2
S1 immature males vs. S2 immature males	E	14.294	1	2995	< 0.001	S1 > S2
S1 maturing males vs. S2 maturing males	F	1.497	1	2995	0.2212	NS
S1 male residuals vs. S1 female residuals	G	97.795	1	3011	< 0.001	M > F
S2 male residuals vs. S2 female residuals	H	547.480	1	3000	< 0.001	M > F
S1 male residuals vs. S2 male residuals	I	2.135	1	2995	0.1440	NS
S1 female residuals vs. S2 female residuals	I	186.190	1	3016	< 0.001	S1 > S2
S1 residuals vs. S2 residuals	J	37.464	1	6011	< 0.001	S1 > S2

Results from Pearson’s chi-square test for equality of proportions of juvenile steelhead sampled at Winthrop National Fish Hatchery in release years 2011–2015 combined. Letter in the reference column corresponds to test citations within the main text. P-values < 0.05 were considered statistically significant. Result column indicates whether the test result was not significant (NS) or whether the proportion of the first test group was determined to be greater than (>) or less than (<) that of the second test group.

### Objective 1b –Male maturation markers by rearing treatment

**Fig 5:**
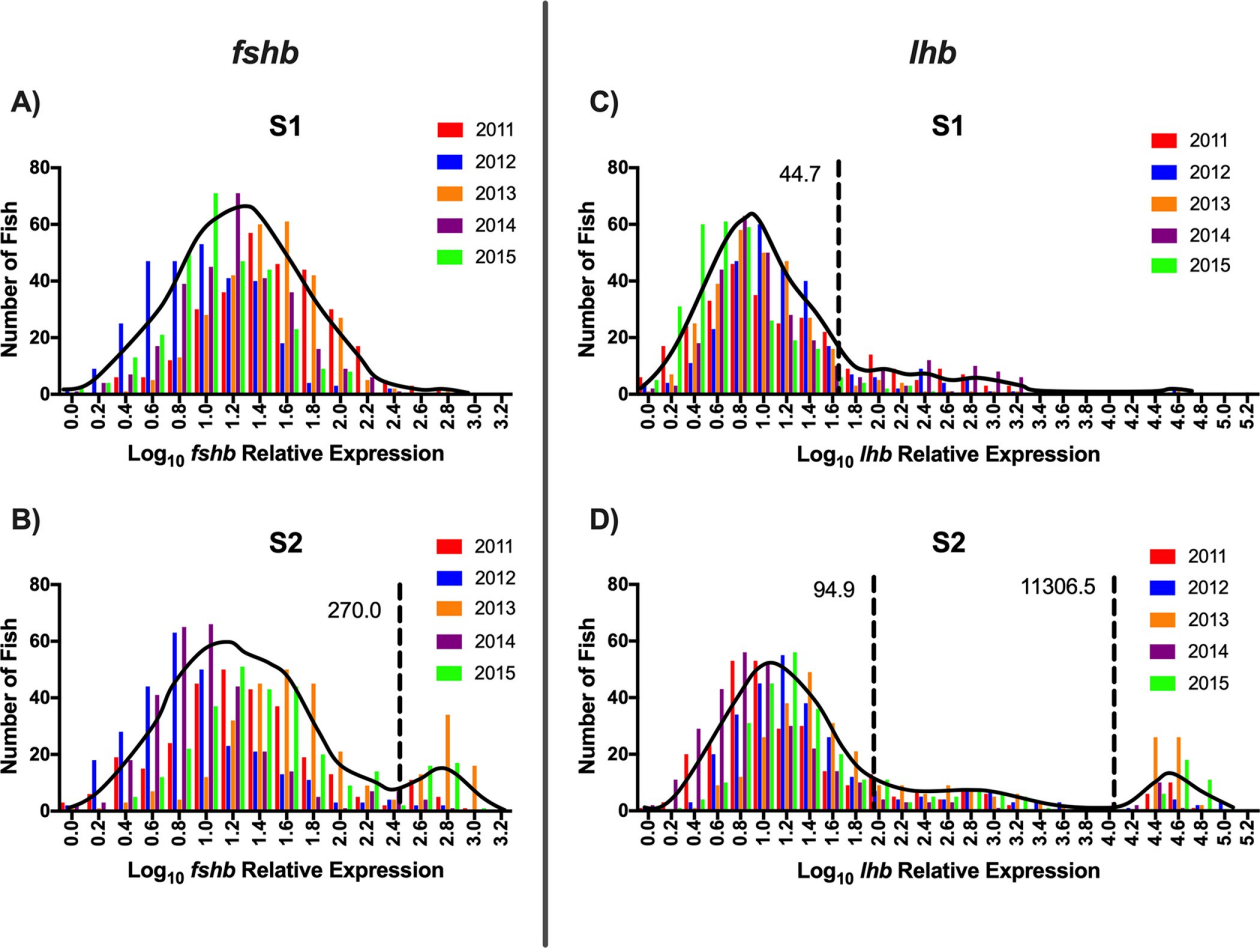
Frequency distribution–pituitary *fshb* and *lhb* mRNA. Frequency distributions of log10 transformed pituitary follicle stimulating hormone beta-subunit (*fshb*; A-B) and luteinizing hormone beta-subunit (*lhb*; C-D) mRNA relative expression for juvenile male steelhead sampled at Winthrop National Fish Hatchery in release years 2011–2015 separated according to rearing treatment (S1: A, C; S2: B, D). Interleaved colored bars represent individual release years. Solid black lines indicate the density distribution for pooled release years. Dashed black reference lines indicate mode intersection as determined by finite mixture model analysis and establish the threshold value for maturation status. For S2 *fshb* and S1 *lhb*, which are both bimodal, males with pituitary gene expression less than the dashed reference line value (lower mode) were classified as immature, while males with gene expression greater than the dashed reference line value (upper mode) were classified as maturing. For S2 *lhb*, which is trimodal, males with *lhb* less than 94.9 relative expression (lower mode) were classified as immature, males with relative expression between 94.9 and 11306.5 (middle mode) were classified as maturing, and males with relative expression greater than 11306.5 (upper mode) were classified as, and visually confirmed to be, mature.

**Fig 6:**
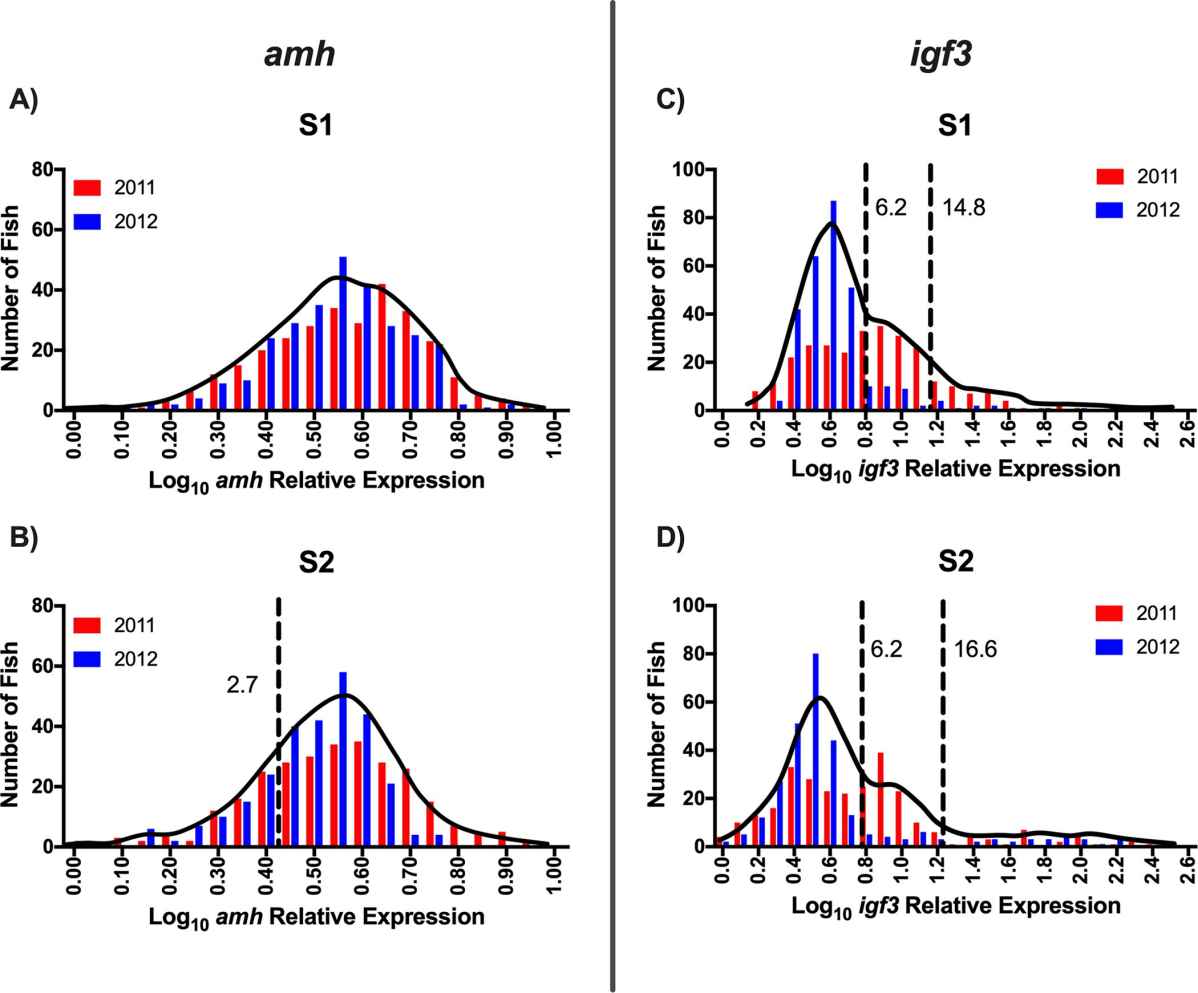
Frequency distribution–testis *amh* and *igf3* mRNA. Frequency distributions of log10 transformed testis anti-Mullerian hormone (*amh*; A-B) and insulin-like growth factor-3 (*igf3*; C-D) mRNA relative expression for juvenile male steelhead sampled at Winthrop National Fish Hatchery in release year 2011 and 2012 separated according to rearing treatment (S1: A, C; S2: B, D). Interleaved red and blue bars represent individual release years. Solid black lines indicate the density distribution for pooled release years. Dashed black reference lines indicate mode intersection as determined by finite mixture model analysis and establish the threshold value for maturation status. For S2 *amh*, which is bimodal, males with *amh* relative expression less than the dashed reference line value (lower mode) were classified as maturing, while males with *amh* relative expression greater than the dashed reference line value (upper mode) were classified as immature. For *igf3* both rearing treatments are trimodal, however, males in the third mode (greater than 14.8 relative expression for S1 and greater than 16.6 relative expression for S2) were not classified as mature because measurement of mature (spermiating) males is not possible with our method. Therefore, in both rearing treatments, males with *igf3* less than 6.2 relative expression (lower mode) were classified as immature and males with *igf3* greater than 6.2 relative expression (middle and upper modes) were classified as maturing. Due to logistical constraints, we were unable to measure testis *amh* and *igf3* in all years of the study.

**Fig 7:**
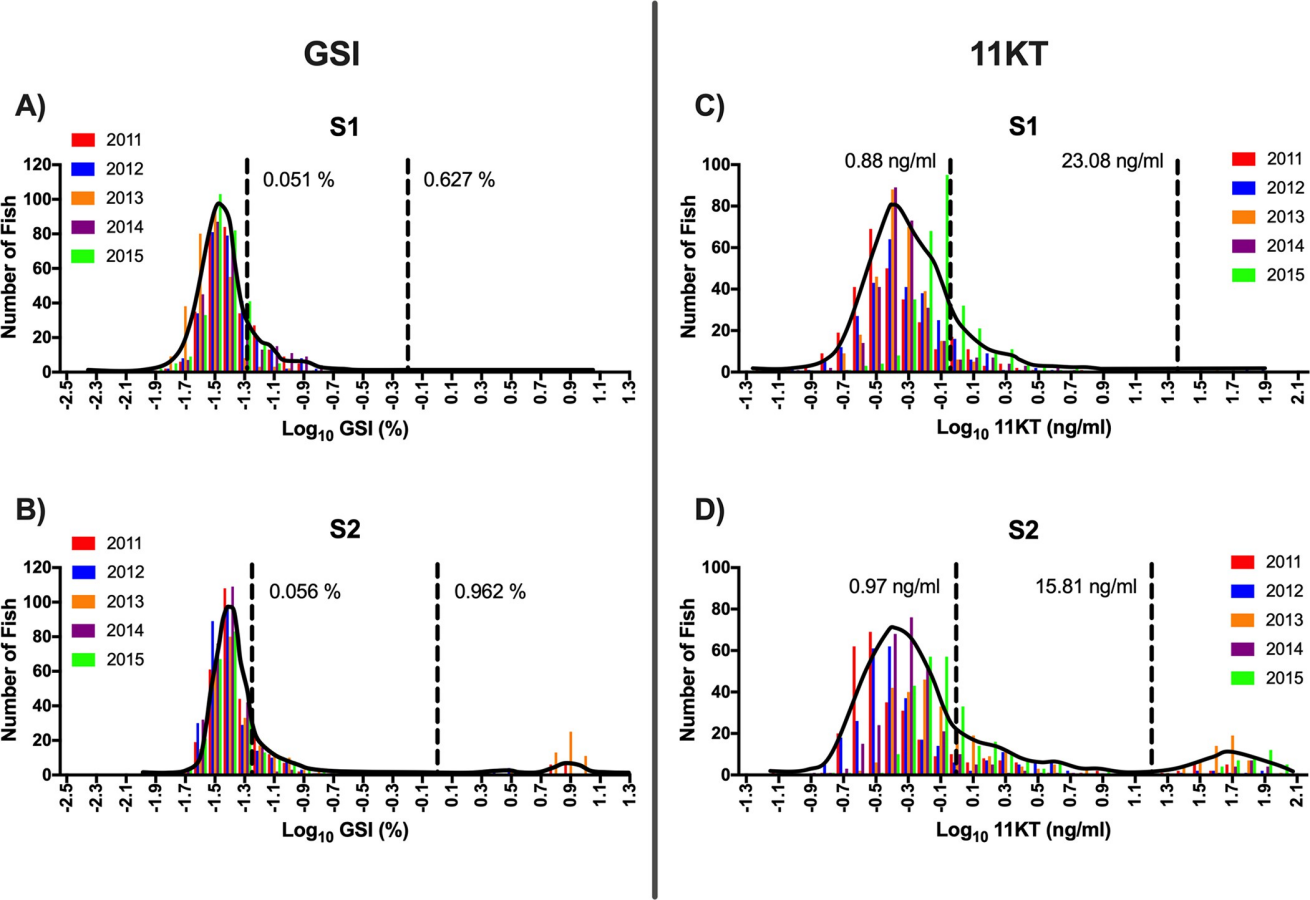
Frequency distribution–GSI and plasma 11KT. Frequency distributions of log10 transformed gonadosomatic index (GSI, %; A-B) and plasma 11-ketotestosterone (11KT, ng/ml; C-D) for juvenile male steelhead sampled at Winthrop National Fish Hatchery in release years 2011–2015 separated according to treatment (S1: A, C; S2: B, D). Interleaved colored bars represent individual release years. Mature male testes were not weighed in release year 2014 and 2015 so they are not represented for GSI. Solid black lines indicate the density distribution for pooled release years. Dashed black reference lines indicate mode intersection as determined by finite mixture model analysis and establish the threshold value for maturation status. GSI and plasma 11KT distributions are trimodal for both rearing treatments therefore in all panels, males with values less than the leftmost dashed reference line value (lower mode) were classified as immature, males with values between the two dashed reference lines (middle mode) were classified as maturing, and males with values greater than the rightmost dashed reference line value (upper mode) were classified as, and visually confirmed to be, mature.

**Fig 8:**
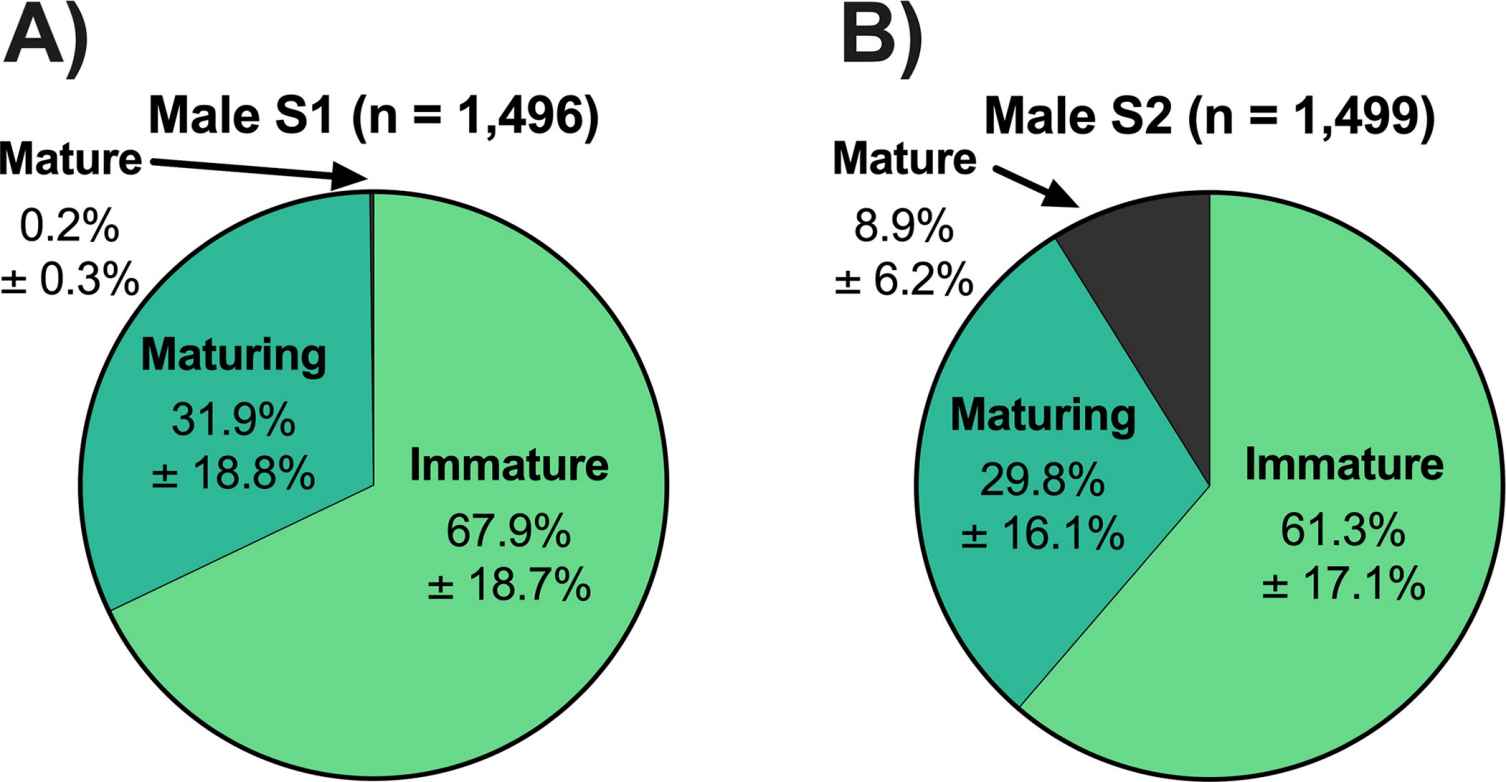
Proportion of male maturation status by rearing treatment. Proportion of juvenile male steelhead sampled at Winthrop National Fish Hatchery in release years 2011–2015 separated according to rearing treatment (S1: A, S2: B) for maturation status as determined by finite mixture model analysis (immature, maturing) or visual identification (mature). Proportion is represented as mean ± SD of individual release years.

The frequency distribution of pituitary *fshb* relative expression for the S1 rearing treatment was unimodal, while the *fshb* distribution for the S2 rearing treatment was bimodal (Fig 5A and 5B; Table 1). Because *fshb* for the S1 rearing treatment was unimodal, no threshold value was determined and thus, there is no comparison in threshold values between rearing treatments. For pituitary *lhb* relative expression, the S1 rearing treatment was best fit with a bimodal distribution, while that of the S2 treatment was trimodal (Fig 5C and 5D; Table 1). The *lhb* threshold value separating immature and maturing males (threshold value 1) for the S2 treatment was more than twice the value found for the S1 treatment (Table 1). The frequency distribution of testis *amh* relative expression for the S1 rearing treatment was unimodal, whereas that of the S2 distribution was bimodal (Fig 6A and 6B; Table 1). Because *amh* for the S1 rearing treatment was unimodal, no threshold value was determined and thus, there is no comparison in threshold values between rearing treatments. For testis *igf3* relative expression, both the S1 and S2 treatments were trimodal and had the same threshold value separating the immature males from the maturing males (threshold value 1) (Fig 6C and 6D; Table 1). Despite the trimodality of the *igf3* distributions for both rearing treatments, males in the upper modes were not classified as mature because the method used does not allow measurement of testis gene expression in spermiating males. Therefore, for *igf3*, only threshold value 1 (Table 1) was considered and males less than the threshold value were classified as immature while males greater than the threshold value were classified as maturing males. Frequency distributions for GSI and plasma 11KT were trimodal for both the S1 and S2 rearing treatments (Fig 7; Table 1). For both GSI and plasma 11KT, the threshold value separating immature males from maturing males (threshold value 1) was similar for both treatments, while the threshold value separating maturing males from mature males differed between treatments (threshold value 2) (Fig 7; Table 1). Visual identification of mature males via milt expression and nuptial coloration aligned with categorization of fish as mature when maturation marker distributions were trimodal, with the exception of *igf3* which was not measurable in mature fish. After categorizing maturation status based on available measures, we found that the S1 and S2 treatments produced similar proportions of maturing males, but the S1 treatment produced more immature males than the S2 treatment (Fig 8; Table 3E and 3F).

**Fig 9:**
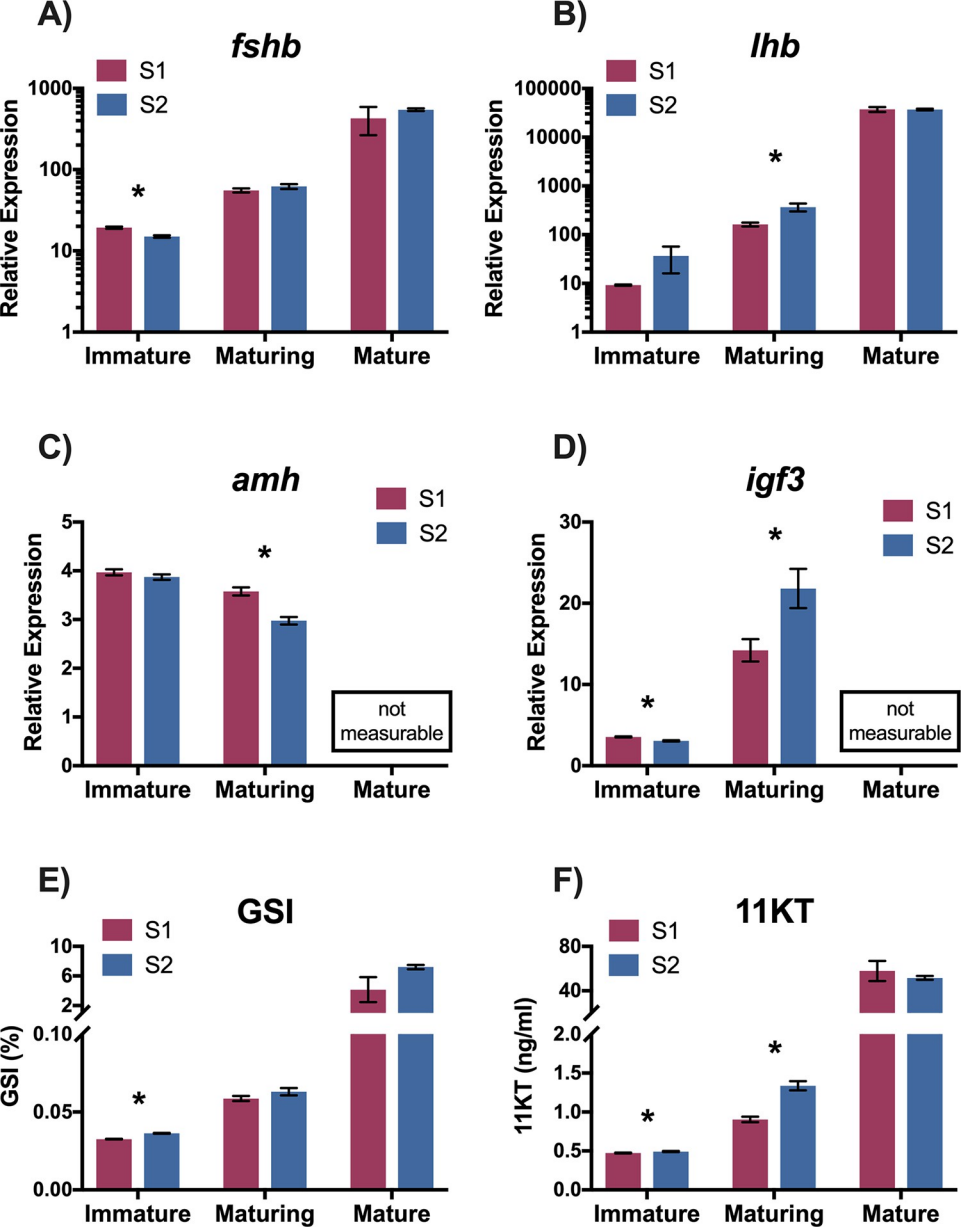
Pituitary and testis mRNA, GSI, and plasma 11KT by rearing treatment and maturation status. Pituitary mRNA (follicle stimulating hormone beta-subunit, *fshb*: A; luteinizing hormone beta-subunit, *lhb*: B), testis mRNA (anti-Mullerian hormone, *amh*: C; insulin-like growth factor-3, *igf3*: D) (relative mRNA expression); gonadosomatic index (GSI, %; E), and plasma 11-ketotestosterone (11KT, ng/ml; F) of juvenile male steelhead sampled at Winthrop National Fish Hatchery in release years 2011–2015 combined. Data are separated according to rearing treatment (S1 in red, S2 in blue) and maturation status as determined by finite mixture model analysis. Data are mean ± SEM. An asterisk indicates a significant difference (p < 0.05) between rearing treatments within a maturation category as determined by two-sample t-test. Mature males were not included in statistical analyses but are included on the graphs for visual reference.

For S1 and S2 immature males, mean values were significantly different for pituitary *fshb*, testis *igf3*, GSI and plasma 11KT, but S1 and S2 mean values were not significantly different for pituitary *lhb* and testis *amh* (Fig 9; Table 4). For pituitary *fshb* and testis *igf3*, S1 immature males demonstrated higher mean values than S2 immature males (Fig 9; Table 4). For GSI and plasma 11KT, S1 immature males demonstrated lower mean values than S2 immature males (Fig 9; Table 4). For maturing males, mean values were significantly different between the S1 and S2 treatments for pituitary *lhb*, testis *amh* and *igf3*, and plasma 11KT, but maturing male mean values were not significantly different for pituitary *fshb* and GSI between rearing treatments (Fig 9; Table 4). For testis *amh*, S1 maturing males demonstrated higher mean values than S2 maturing males (Fig 9; Table 4). For pituitary *lhb*, testis *igf3*, and plasma 11KT maturing males from the S1 rearing treatment demonstrated lower mean values than S2 maturing males (Fig 9; Table 4). Although they were excluded from statistical analysis, as expected, mature males from both rearing treatments exhibited higher values than immature or maturing males for pituitary *fshb* and *lhb*, GSI, and plasma 11KT (Fig 9).

**Table 4:** Two-sample t-test analysis results comparing male maturation markers between rearing treatments (S1, S2) by maturation status (immature, maturing).

Measure	Maturation Category	t	df	p-value
***fshb***	**Immature**	5.394	1831.3	< 0.001
	**Maturing**	-1.219	820.8	0.2234
***lhb***	**Immature**	-1.339	903.3	0.1810
	**Maturing**	-2.947	479.9	0.0034
***amh***	**Immature**	1.147	622.8	0.2518
	**Maturing**	5.207	508.1	< 0.001
***igf3***	**Immature**	5.752	614.8	< 0.001
	**Maturing**	-2.744	390.0	0.0063
**GSI**	**Immature**	-11.180	1915.9	< 0.001
	**Maturing**	-1.564	800.2	0.1183
**plasma 11KT**	**Immature**	-2.254	1877.3	0.0243
	**Maturing**	-6.299	725.4	< 0.001

Results from two-sample t-tests of juvenile male steelhead sampled at Winthrop National Fish Hatchery in release years 2011–2015 combined comparing means of male maturation markers between rearing treatments by maturation status. P-values < 0.05 were considered statistically significant. Abbreviations and units of measure are as follows: pituitary follicle stimulating hormone beta-subunit (*fshb*) and luteinizing hormone beta-subunit (*lhb*), testis anti-Mullerian hormone (*amh*) and insulin-like growth factor-3 (*igf3*) (relative mRNA expression); gonadosomatic index (GSI; %); plasma 11-ketotestosterone (11KT; ng/ml).

### Objective 2 –Sex-specific residualism predictions by rearing treatment

**Fig 10:**
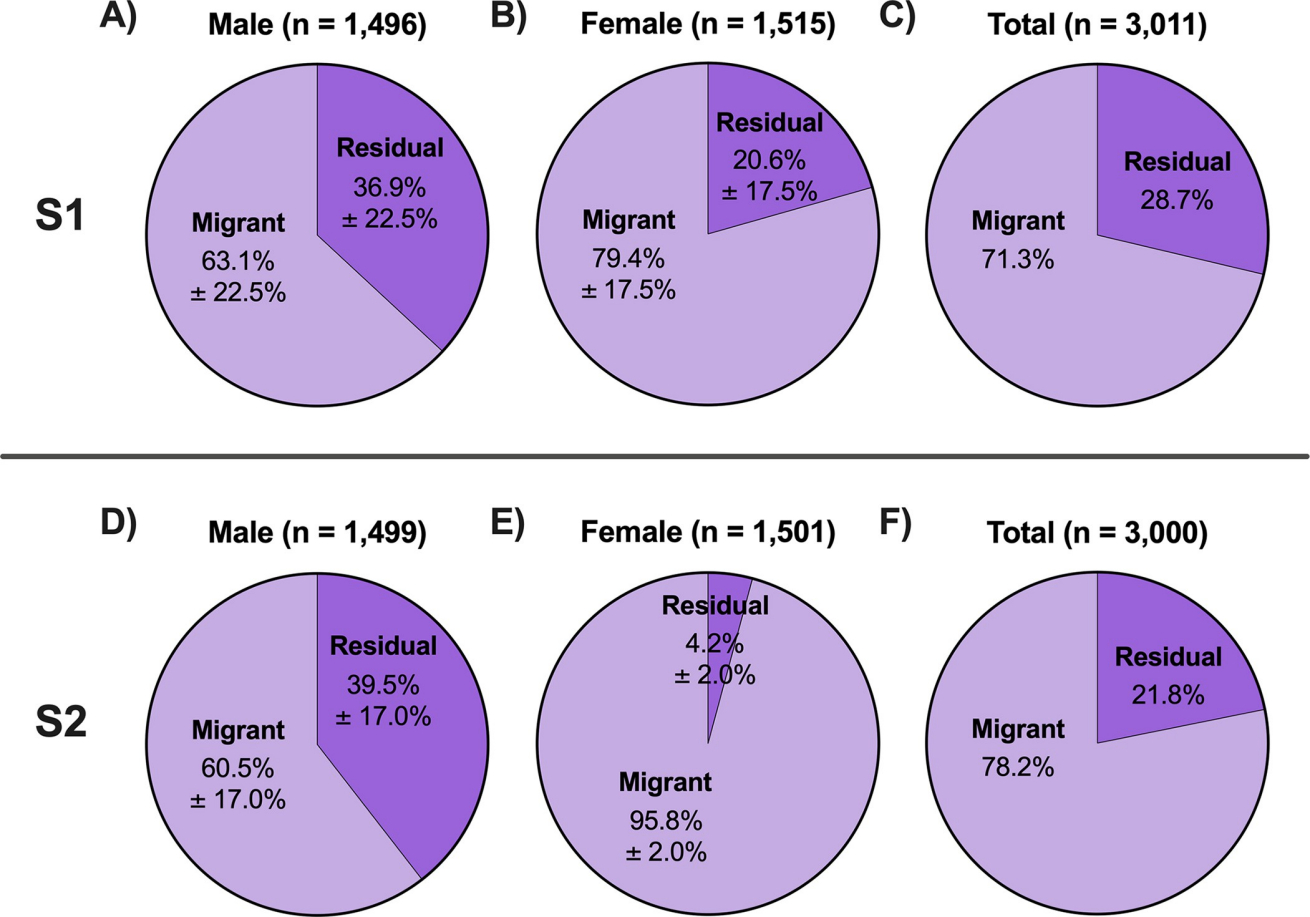
Proportion of predicted residuals by rearing treatment and sex. Proportion of juvenile steelhead sampled at Winthrop National Fish Hatchery in release years 2011–2015 predicted to residualize or migrate separated according to rearing treatment (S1: A-C, S2: D-F) and sex (males: A, D; females: B, E; sexes combined: C, F). Proportions for males and females are represented as mean ± SD of individual release years. No SD is provided for the total (sexes combined) charts (C, F) because samples were pooled from all release years.

While the S1 and S2 treatments exhibited similar proportions of residual males (36.9% S1 vs. 39.5% S2) (Fig 10A and 10D; Table 3I), the proportions of residual females were significantly different between the two treatments (20.6% S1 vs. 4.2% S2) (Fig 10B and 10E; Table 3I). Within the S1 rearing treatment the estimated proportion of residuals was male biased (36.9% males vs. 20.6% females) (Fig 10A and 10B; Table 3G). The male bias of residuals was even more pronounced in the S2 rearing treatment (39.5% males vs. 4.2% females) (Fig 10D and 10E; Table 3H).

After combining both sexes, the proportion of residuals produced by the S1 treatment was higher than the proportion produced by the S2 treatment (28.7% S1 vs. 21.8% S2) (Fig 10C and 10F; Table 3J). For comparison purposes, the smallest and largest 10% of fork lengths for each treatment, regardless of sex, are as follows (range, mean ± SD): S1 “small residuals”: 75–105 mm, 95.3 ± 7.4 mm; S2 “small residuals”: 99–135 mm, 123.6 ± 7.1 mm; S1 “large residuals”: 195–229 mm, 206.4 ± 8.2 mm; S2 “large residuals”: 223–273 mm, 234.0 ± 11.7 mm.

## Discussion

Based upon the results of the current study and previous efforts [48,49], we verified that the analytical methods and male maturation measures used were applicable in both winter- and summer-run steelhead. Winter-run steelhead are characterized by adult migration to the spawning grounds between November and April, while summer-run steelhead migrate to the spawning grounds between May and October and overwinter in freshwater [68] so we chose to confirm that our methods are robust to differences in run-timing. Additionally, the summer-run steelhead in the current study were sampled in March (12–14 months prior to their first spawning opportunity), approximately two months earlier than studies using the same markers in winter-run steelhead (sampled 10–12 months prior to their first spawning opportunity) [49] so we wanted to confirm our methods are also robust to deviations in sample collection timing. In comparison to winter-run steelhead [48,49] we found similar results in summer-run steelhead with a few minor differences. While we found that male summer-run steelhead of both ages had similar threshold values for separating immature from maturing fish via GSI and plasma 11KT, the observed threshold values for pituitary *fshb* and *lhb* and testis *igf3* were higher and testis *amh* thresholds were lower when compared to male winter steelhead [49]. In the two years where testis *amh* and *igf3* transcripts were measured for summer-run steelhead, we were able to enhance detection of maturing males, whereas these same transcripts contributed no additional value in winter-run steelhead [49]. Comparing male maturation measures between summer-run and winter-run steelhead suggests that summer-run steelhead initiate puberty and advance to the meiotic phase of spermatogenesis earlier than winter-run steelhead, potentially allowing for greater utility of *amh* and *igf3* in summer steelhead. We also note that in summer-run steelhead, mean pituitary *fshb* and *lhb*, testis *amh* and *igf3*, GSI, and plasma 11KT were statistically different between males classified as immature and maturing via histology two months earlier than when this class distinction can be confirmed in winter-run steelhead (March for summer-run steelhead in this study vs. May in winter-run steelhead) (S1 File, [49]). Because our methods produced similar results in both winter- and summer-run populations, in the present study we applied the same male maturation metrics across culture durations (one or two years) and broodstock origins (natural or hatchery).

Interannual variation in temperatures for hatcheries operating on surface water is unavoidable [58]. Thermal variability affects growth [69] and maturation rates [70], and accordingly we observed such variation in both the morphological and physiological metrics of smoltification and maturation across the five release years analyzed. Nonetheless, the annual temperature variation did not obscure the differences attributed to age at release or sex in most release years and pooling data across years by rearing treatment provided reasonable threshold estimates for smoltification, maturation, and predicted residualism. Additionally, conducting this study over multiple release years provided the advantage of observing interannual variability in rearing environment over time, a benefit that is unavailable in single year investigations.

The steelhead program at Winthrop National Fish Hatchery (WNFH) provided a unique opportunity because a single hatchery rarely raises juvenile steelhead to smolt at different ages over a substantial number of consecutive release years. Therefore, we were able to evaluate the interaction between release age and sex on smoltification and maturation, allowing us to predict post-release migratory potential. The sensitivity of smoltification and maturation indices used in this study enabled detection of profound shifts in physiological ontogeny of hatchery steelhead leading to different life history diversity profiles for each sex caused by rearing duration (S1 vs. S2). Culture regimes designed to compress smolting to age-1 (S1) limits expression of life history diversity and may contribute to unintentional domestication selection for juveniles with high growth rates under hatchery rearing conditions [30]. A logical method to counteract constriction of natural smolt life history diversity is to increase hatchery rearing duration to induce smoltification at age-2 (S2), but this may disrupt natural patterns of age-at-maturation, particularly for male steelhead [32,36,58]. Because smolt transformation can occur after release during juvenile migration, and ontogeny of maturation in steelhead is largely cryptic, morphological and physiological tools are required to quantify, describe, and predict the effects of rearing strategies on life history diversity of hatchery steelhead populations prior to release.

Although this study focused on male sexual maturation, one of the most important results was that the S2 rearing regime reduced the incidence of residualism in females approximately five-fold compared to the S1 rearing regime (20.6% predicted S1 residuals vs 4.2% predicted S2 residuals). Precocious male maturation has long been viewed as the primary driver of residualism in hatchery steelhead [3,35,48,49]. However, it is clear that a more comprehensive view of sex-specific life-history variation is essential to minimize residualism, maximize smolt production, and increase return of anadromous adults. Minimizing residualism in female steelhead is generally not associated with minimizing precocious maturation due to the propensity for females to mature after completing smoltification [51]. In fact, none of the 3,016 S1 and S2 female steelhead in this study showed signs of maturation upon gonadal examination during dissection. Instead, minimizing female residuals primarily depends on attaining sufficient growth to exceed a size threshold for smoltification, which is theorized to trigger migratory response to environmental cues [45,71]. Steelhead in the S2 treatment had an additional year of growth opportunity to reach smoltification size thresholds, largely explaining why S2 fish were larger overall regardless of sex and why ~96% of S2 females were classified as migrants versus only ~80% of S1 females. For perspective, only 0.06% (1 of 1,783) of marked residual steelhead were found to eventually return to the Methow River as an anadromous adult [36], representing a direct loss to adult productivity.

During the five release years sampled in this study, apparent survival rates of S2 steelhead were higher or equivalent to those of S1 steelhead during juvenile outmigration, with larger size at release being the primary driver of significant differences between rearing treatments [58], which aligns with studies in other steelhead populations [72,73]. For objective one (a), we hypothesized that the proportion of smolts would differ between rearing treatments and sexes; specifically, that the S2 treatment would produce more smolts and there would be a larger proportion of female smolts within each treatment. We found only a 2.4% (statistically significant) difference in smolt proportion between treatments (30.9% S1 vs. 33.3% S2) which is unlikely to have a meaningful effect on adult returns. Additionally, we found no difference in smolts proportions between sexes; both the S1 and S2 rearing treatments produced similar proportions of male and female smolts. For females, S2 rearing appears to be an effective practice for maximizing the likelihood of smoltification and anadromous returns in hatchery steelhead programs. Taken together, the substantial proportion of S2 females classified as migrants, apparent outmigration survival rates of S2 juveniles, and production of similar proportions of male and female smolts should favor increased survival of females under an S2 rearing strategy.

Summer-run steelhead males that are mature or maturing at the time of release complicate efforts to manage gene flow between hatcheries and natural-origin populations. Males that have initiated, but not completed, maturation cannot be reliably quantified because the maturation process begins months before associated external morphological changes are observed [47–49] and maturing males are visually indistinguishable from immature fish of either sex without additional physiological information. Regardless of age (S1 or S2), summer-run steelhead in this study exhibited multimodal distributions for pituitary *fshb* and *lhb* expression, testis *amh* and *igf3* expression, gonadosomatic index (GSI) and plasma 11-ketotestosterone (11KT) corresponding to their degree of maturation. However, we observed differences in the threshold values of *lhb*, *fshb*, and *amh* separating immature from maturing males between the S1 and S2 rearing treatments, which could be attributed to continued maturational development during the extra year of rearing in the S2 treatment. Therefore, it is possibly easier to identify maturing males under an S2 rearing regime compared to an S1 rearing regime. For objective one (b), we were able to visually determine that S2 rearing resulted in significantly higher levels of precocious male maturation than S1 rearing (8.9% S2 vs. 0.2% S1). Physiological metrics, and their associated threshold values, enabled further differentiation in maturation status, with the S1 treatment producing a higher proportion of immature males and both treatments producing similar proportions of maturing males.

Fork length, in addition to physiological markers for male maturation, provided another key indicator of migration potential. Both treatments displayed bimodality in fork length with similar migration threshold values (146.6 mm for S1 and 147.6 mm for S2) that were interpreted as the minimum size for migration. These size thresholds coincide with those derived using passive integrated transponder (PIT) tag detection data during the years encompassed by this study, indicating steelhead below 146 mm, and precociously mature males of any size, were more likely to residualize in freshwater than to outmigrate to the ocean [36]. Combining fork length data with male categorization of maturation allowed us to predict residualism and we hypothesized that the predicted proportion of residuals would differ between rearing treatments and sexes (objective two). The percentage of residuals (both sexes combined) was greater in the S1 than the S2 treatment (28.7% S1 vs. 21.8% S2). Within each rearing treatment, there was a higher proportion of male than female residuals, but the proportion of male residual steelhead was similar between treatments. Therefore, we conclude that the differences between S1 and S2 residuals overall was largely due to a combination of small, immature females. Additionally, the consistently smaller size of S1 reared residuals compared with S2 reared residuals suggests that size is a stronger determinant of residualism for S1 compared to S2 reared steelhead.

Patterns and causes of residualism have sex-specific implications for hatchery populations and the natural populations they are intended to supplement. Precocious male maturation may reduce the genetic diversity of both the hatchery and natural breeding components and reduces the number of anadromous adults available if the hatchery supplements a local recreational harvest when sufficient adult returns are forecast. Male steelhead typically spawn with multiple females in the natural environment [74,75] and hatcheries faced with male broodstock shortages may have to use milt from a single male to fertilize eggs from multiple females, creating genetic implications such as inbreeding depression and reduced genetic diversity [76,77]. Conversely, loss of one in five potential anadromous females on the spawning grounds or in hatchery broodstock from an S1 rearing regime presents a more immediate limitation to population productivity than a similar reduction in the number of returning males. Results of physiological metrics in this study revealed divergent life history trajectories for residual males between treatments, with more mature males in the S2 treatment and more immature males in the S1 treatment. Although *O*. *mykiss* natural life histories include male maturation without migration [48], S2 summer-run rearing appears to accelerate sexual development to a degree that reduces the natural age of first maturation for anadromous males from between three and five years [6] to two years. Because mature male hatchery steelhead do not migrate after release [36], and release coincides with anadromous spawning, precocious males can potentially participate in spawning events (using satellite or sneaker tactics) and sire offspring with anadromous females [75] necessitating appropriate strategies to manage gene flow between hatchery and natural populations. One such strategy, now employed by the WNFH, is a volitional release wherein fish that fail to exit the hatchery during the release window are collected and transported to an isolated local lake to serve a recreational fishery, thereby avoiding potential spawning interactions with the natural, anadromous population.

Although increased gill ATPase activity is considered one of the best diagnostic indicators of smoltification [78], S1 and S2 steelhead in this study showed only modest increases in gill ATPase activity as they progressed from parr to transitional to smolt phenotypes. This result is similar to previous work on steelhead smolts at WNFH, which found that gill ATPase activity levels were relatively low and uninformative [79]. It is possible gill ATPase activity is less useful for distinguishing smoltification status in this upper Columbia River stock because juvenile Methow River steelhead travel hundreds of kilometers to reach the ocean, and significant increases in gill ATPase activity often occur during, rather than prior to, freshwater downstream migration [80,81]. Thus, we utilized gill ATPase activity to track developmental ontogeny, but ultimately chose not to include this metric to predict individual migration status. Regardless, integrating the remaining morphological and physiological metrics yielded useful age- and sex-specific predictions about hatchery performance, such as the proportion of production likely to migrate or to become residuals and why (poor smoltification or maturation), and can inform changes to culture practices needed to reduce residual production.

Predictions regarding migratory potential, based on morphological and physiological metrics, are of limited value without validation through data on migratory performance of smolts and adult return rates (i.e. survival). Fortunately, parallel investigations of S1 and S2 post-release performance for this population were conducted during the same release years examined in the present study (2011–2015). Many of the sex- and age-specific shifts in life history diversity of hatchery steelhead documented in this study had consequences throughout the migratory life cycle extending to adulthood. Interestingly, smolt age-based survival differences continued throughout ocean residence and adult return migration, with the benefits of release age shifting among migration segments [82]. Despite longer residence in the Pacific Ocean, S1 steelhead had higher ocean segment survival than S2 steelhead which equilibrated between treatments during their adult upstream migration segment through the mainstem Columbia River [82]. However, subsequent migration to the Methow River spawning tributaries resulted in higher survival for S2 than S1 adults, leading to slightly higher overall survival for returning S2 adults [82].

In the same way that predicting migratory potential has limited value without data on survival, survival data has limited value to stock recovery programs without data on reproductive capabilities (i.e. reproductive success). Reproductive success of adult returns is influenced by an interaction between smolt release age and sex. Male S2 adults demonstrated deficits in competitive behavior resulting in lower participation in spawning events and siring fewer offspring than S1 males [75]. Precociously mature S2 males successfully sired offspring with adult anadromous females, but at lower rates than S2 anadromous males [75]. Females, however, demonstrated equal production of offspring regardless of smolt age at release [75]. Because reduced reproductive success of hatchery steelhead populations may also have a genetic basis [83,84], there is increasing emphasis on managing gene flow between hatchery and natural populations of steelhead [85–87]. However, the reduced potential for gene flow seen in anadromous S2 males may be offset by increased gene flow attributed to participation of large numbers of precociously mature S2 males in spawning events with natural-origin females [75]. These examples highlight the complexity of how simple changes in hatchery rearing regimes set trajectories that differentially affect developmental physiology between sexes, impacting demographic, ecological, and genetic interactions between natural and hatchery populations, as well as hatchery program goals for stock recovery.

Some of the specialized tools deployed in this experiment (gill ATPase activity, plasma 11KT, and mRNA transcripts) are not easily measured within a hatchery setting due to logistical, technical, and financial constraints. However, other metrics (sex, fork length, and GSI) are easily measured, technically straight-forward, and inexpensive. With these considerations in mind, we completed our third objective by conducting a semi-quantitative cost/benefit analysis of the suite of metrics used in this study for their utility in forecasting steelhead life-history (Table 5). While we found value in most of the metrics, basic parameters like fork length, sex, GSI, and plasma 11KT were categorized as relatively cost effective and diagnostically useful for capturing significant demographic information. Hatchery managers interested in embarking on a similar analysis could sample a minimum of 300 fish for fork length, sex, and GSI for only the added cost associated with additional labor, plus the one-time purchase required for an analytical balance (to accurately weigh testes) with a readability of 0.001 g. In order to measure plasma 11KT, managers could invest in a one-time purchase of equipment, training for onsite staff, and commercially available assay kits. Alternatively, hatcheries could collaborate with an external laboratory capable of running their samples for approximately $3.50 per sample at the time of this publication. Note that both reagent prices and labor cost typically increase over time, leading to potential increases in cost per sample in the future. We caveat that gill ATPase activity, while less diagnostically useful in this population and study, is a relatively inexpensive measure that may be effective in hatcheries with shorter migration distances to seawater. Additionally, we caveat that some measures did not meet a sufficient cost/benefit ratio to warrant our recommendation. On the one hand, body weight and smolt index are very inexpensive to put into practice, however, neither measure contributed significantly to our predication of residuals. Similarly, mRNA transcripts measures were key to our residual predictions, but the expense, expertise, and time commitment required to obtain the data make these measures unrealistic for year-to-year hatchery management. Finally, while gonadal histology is considered the “gold standard” for determining maturation status, our previous research found that histological staging can underestimate maturation proportions when compared to our other physiological metrics [49] and for that reason, along with a notable labor cost and time commitment, we do not recommend undertaking histology for similar investigations. Applying the appropriate tools can add value to existing hatchery monitoring and evaluation efforts, especially when interpreting survival and residualism results obtained from tagging and detection data [36,58].

**Table 5:** Cost benefit analysis of parameters measured in this study and their application to management decisions within a steelhead hatchery setting.

Measurement	Cost	Benefit to Hatchery management	Predictive value S1	Predictive value S2	Priority
**Sex**	$	+++	*****	*****	2
**Fork Length**	$	+++	*****	*****	1
**Body Weight**	$	+++	*	*	9
**Smolt Phenotype**	$	+++	**	**	7
**GSI**	$ $	+++	****	*****	3
**gill ATPase activity**	$ $ $	++	*	*	8
**plasma 11KT**	$ $ $	++	***	****	4
**Pituitary mRNA**	$ $ $ $	+	**	***	5
**Testis mRNA**	$ $ $ $	+	**	***	6

Analysis of associated costs of each parameter measured relative to the benefits to hatchery management and predictive value for each treatment, resulting in prioritization of each measure and its practicality for use by managers. Symbols are as follows

$—requires only labor hours during sampling.

$ $—requires dissection, a microbalance and additional time commitment, but otherwise only labor hours during sampling.

$ $ $—requires lab equipment and experience, minimal additional sampling experience, reagents are relatively inexpensive, and assays produce data relatively quickly.

$ $ $ $—requires specialized dissection and sterile technique at sampling, expensive equipment, molecular biology expertise, expensive reagents, data production fairly slow.

+—valuable for identifying maturation status, but cost likely outweighs benefits within a hatchery setting.

++—extremely relevant, obtaining results likely requires collaboration outside of the hatchery.

+++—extremely valuable, can be performed by onsite hatchery staff.

*—no/low predictive value for residuals.

**—low predictive value for residuals.

***—has predictive value but cost is a significant consideration.

****—high predictive value.

*****—best predictive value at a minimal cost.

Evolution of hatchery culture strategies to meet challenges presented by increased climatic variability, including water reuse systems to control for warmer ambient water temperatures and decreased water availability, is a topic of increasing importance in the Methow River Basin [88] and elsewhere [2,89–91]. The morphological and physiological tools employed in this study may have utility for forecasting shifts in life-history diversity associated with new hatchery culture strategies. Changes in culture, and their associated effects on post-release survival and adult demography, can yield beneficial information to improve hatchery performance for steelhead programs supporting stock recovery and mitigation for anthropogenic environmental perturbations.

## Supporting information

S1 File Testis histology methods and results. Methods and results for testis histology staging of juvenile male steelhead sampled at Winthrop National Fish Hatchery in release years 2011–2015.
(DOCX)

S2 File Supplementary results. Results for juvenile steelhead sampled at Winthrop National Fish Hatchery in individual release years. Results for maturation measures (male only) separated according to rearing treatment (S1, S2) and visually determined qualitative smolt phenotype (parr, transitional, smolt, mature male). Results for finite mixture model (fmm) analysis of body weight and gill Na+/K+ ATPase activity. Morphological measures separated according to rearing treatment (S1, S2) and maturation status as determined by fmm analysis (immature, maturing, mature).
(DOCX)

S1 Fig Fork length by rearing treatment and histological stage. Fork length (mm) of juvenile male steelhead sampled at Winthrop National Fish Hatchery in release years 2011–2015 separated according to rearing treatment (S1 in purple, S2 in blue) and histological stage (immature, stage 0; maturing, stage 1–4; mature, spermiating). Data are mean ± SEM. Graph in box (F) includes males combined across all release years. Different letters indicate significant differences (p < 0.05) as determined by two-way ANOVA with Tukey’s post-hoc test. Mature males were not included in statistical analyses but are included on the graphs for visual reference.
(TIFF)

S2 Fig Body weight by rearing treatment and histological stage. Body weight (g) of juvenile male steelhead sampled at Winthrop National Fish Hatchery in release years 2011–2015 separated according to rearing treatment (S1 in purple, S2 in blue) and histological stage (immature, stage 0; maturing, stage 1–4; mature, spermiating). Data are mean ± SEM. Graph in box (F) includes males combined across all release years. Different letters indicate significant differences (p < 0.05) as determined by two-way ANOVA with Tukey’s post-hoc test. Mature males were not included in statistical analyses but are included on the graphs for visual reference.
(TIFF)

S3 Fig Gill ATPase activity by rearing treatment and histological stage. Gill Na+/K+ ATPase activity (μmol ADP · mg protein^-1^ · hr^-1^) of juvenile male steelhead sampled at Winthrop National Fish Hatchery in release years 2012–2015 separated according to rearing treatment (S1 in purple, S2 in blue) and histological stage (immature, stage 0; maturing, stage 1–4; mature, spermiating). Data are mean ± SEM. Graph in box (E) includes males combined across all release years. Different letters indicate significant differences (p < 0.05) as determined by two-way ANOVA with Tukey’s post-hoc test. Mature males were not included in statistical analyses but are included on the graphs for visual reference.
(TIFF)

S4 Fig Pituitary *fshb* by rearing treatment and histological stage. Pituitary follicle stimulating hormone beta-subunit (*fshb*) mRNA relative expression of juvenile male steelhead sampled at Winthrop National Fish Hatchery in release years 2011–2015 separated according to rearing treatment (S1 in purple, S2 in blue) and histological stage (immature, stage 0; maturing, stage 1–4; mature, spermiating). Data are mean ± SEM. Graph in box (F) includes males combined across all release years. Different letters indicate significant differences (p < 0.05) as determined by two-way ANOVA with Tukey’s post-hoc test. Mature males were not included in statistical analyses but are included on the graphs for visual reference.
(TIFF)

S5 Fig Pituitary *lhb* by rearing treatment and histological stage. Pituitary luteinizing hormone beta-subunit (*lhb*) mRNA relative expression of juvenile male steelhead sampled at Winthrop National Fish Hatchery in release years 2011–2015 separated according to rearing treatment (S1 in purple, S2 in blue) and histological stage (immature, stage 0; maturing, stage 1–4; mature, spermiating). Data are mean ± SEM. Graph in box (F) includes males combined across all release years. Different letters indicate significant differences (p < 0.05) as determined by two-way ANOVA with Tukey’s post-hoc test. Mature males were not included in statistical analyses but are included on the graphs for visual reference.
(TIFF)

S6 Fig Testis *amh* and *igf3* by rearing treatment and histological stage. Testis anti-Mullerian hormone (*amh*; A-C) and insulin-like growth factor-3 (*igf3*; D-F) mRNA relative expression of juvenile male steelhead sampled at Winthrop National Fish Hatchery in release years 2011 and 2012 separated according to rearing treatment (S1 in purple, S2 in blue) and histological stage (immature, stage 0; maturing, stage 1–4; mature, spermiating). Data are mean ± SEM. Graphs in boxes (C and F) include males combined across all release years. Different letters indicate significant differences (p < 0.05) as determined by two-way ANOVA with Tukey’s post-hoc test. Measurement of mature (spermiating) males is not possible with our method. Due to logistical constraints we were unable to measure testis *amh* and *igf3* in all years of the study.
(TIFF)

S7 Fig GSI by rearing treatment and histological stage. Gonadosomatic index (GSI; %) of juvenile male steelhead sampled at Winthrop National Fish Hatchery in release years 2011–2015 separated according to rearing treatment (S1 in purple, S2 in blue) and histological stage (immature, stage 0; maturing, stage 1–4; mature, spermiating). Data are mean ± SEM. Graph in box (F) includes males combined across all release years. Different letters indicate significant differences (p < 0.05) as determined by two-way ANOVA with Tukey’s post-hoc test. Mature males were not included in statistical analyses but are included on the graphs for visual reference. In release years 2014 and 2015, mature male testes were not weighed so no data is shown.
(TIFF)

S8 Fig Plasma 11KT by rearing treatment and histological stage. Plasma 11-ketotestosterone (11KT; ng/ml) of juvenile male steelhead sampled at Winthrop National Fish Hatchery in release years 2011–2015 separated according to rearing treatment (S1 in purple, S2 in blue) and histological stage (immature, stage 0; maturing, stage 1–4; mature, spermiating). Data are mean ± SEM. Graph in box (F) includes males combined across all release years. Different letters indicate significant differences (p < 0.05) as determined by two-way ANOVA with Tukey’s post-hoc test. Mature males were not included in statistical analyses but are included on the graphs for visual reference.
(TIFF)

S9 Fig Male and female fork length by rearing treatment and smolt phenotype. Fork length (mm) of juvenile steelhead sampled at Winthrop National Fish Hatchery in release years 2011–2015 separated according to rearing treatment (S1 in light blue, S2 in violet) and visually determined qualitative smolt phenotype for males (A-E) and females (F-J). Data are mean ± SEM. Different letters indicate significant differences (p < 0.05) as determined by two-way ANOVA with Tukey’s post-hoc test. Mature males were not included in statistical analyses but are included on the graphs for visual reference. Graphs for pooled release years are contained in Fig 3.
(TIFF)

S10 Fig Male and female body weight by rearing treatment and smolt phenotype. Body weight (g) of juvenile steelhead sampled at Winthrop National Fish Hatchery in release years 2011–2015 separated according to rearing treatment (S1 in light blue, S2 in violet) and visually determined qualitative smolt phenotype for males (A-E) and females (F-J). Data are mean ± SEM. Different letters indicate significant differences (p < 0.05) as determined by two-way ANOVA with Tukey’s post-hoc test. Mature males were not included in statistical analyses but are included on the graphs for visual reference. Graphs for pooled release years are contained in Fig 3.
(TIFF)

S11 Fig Male and female gill ATPase activity by rearing treatment and smolt phenotype. Gill Na+/K+ ATPase activity (μmol ADP · mg protein^-1^ · hr^-1^) of juvenile steelhead sampled at Winthrop National Fish Hatchery in release years 2012–2015 separated according to rearing treatment (S1 in light blue, S2 in violet) and visually determined qualitative smolt phenotype for males (A-D) and females (E-H). Data are mean ± SEM. Different letters indicate significant differences (p < 0.05) as determined by two-way ANOVA with Tukey’s post-hoc test. Mature males were not included in statistical analyses but are included on the graphs for visual reference. Graphs for pooled release years are contained in Fig 3.
(TIFF)

S12 Fig Pituitary *fshb* by rearing treatment and smolt phenotype. Pituitary follicle stimulating hormone beta-subunit (*fshb*) mRNA relative expression of juvenile male steelhead sampled at Winthrop National Fish Hatchery in release years 2011–2015 separated according to rearing treatment (S1 in light blue, S2 in violet) and visually determined qualitative smolt phenotype. Data are mean ± SEM. Graph in gray box (F) includes males combined across all release years. Different letters indicate significant differences (p < 0.05) as determined by two-way ANOVA with Tukey’s post-hoc test. Mature males were not included in statistical analyses but are included on the graphs for visual reference.
(TIFF)

S13 Fig Pituitary *lhb* by rearing treatment and smolt phenotype. Pituitary luteinizing hormone beta-subunit (*lhb*) mRNA relative expression of juvenile male steelhead sampled at Winthrop National Fish Hatchery in release years 2011–2015 separated according to rearing treatment (S1 in light blue, S2 in violet) and visually determined qualitative smolt phenotype. Data are mean ± SEM. Graph in gray box (F) includes males combined across all release years. Different letters indicate significant differences (p < 0.05) as determined by two-way ANOVA with Tukey’s post-hoc test. Mature males were not included in statistical analyses but are included on the graphs for visual reference.
(TIFF)

S14 Fig Testis *amh* and *igf3* by rearing treatment and smolt phenotype. Testis anti-Mullerian hormone (*amh*; A-C) and insulin-like growth factor-3 (*igf3*; D-F) mRNA relative expression of juvenile male steelhead sampled at Winthrop National Fish Hatchery in release years 2011 and 2012 separated according to rearing treatment (S1 in light blue, S2 in violet) and visually determined qualitative smolt phenotype. Data are mean ± SEM. Graphs in gray boxes (C and F) include males combined across all release years. Different letters indicate significant differences (p < 0.05) as determined by two-way ANOVA with Tukey’s post-hoc test. Measurement of mature (spermiating) males is not possible with our method. Due to logistical constraints, we were unable to measure testis *amh* and *igf3* in all years of the study.
(TIFF)

S15 Fig GSI by rearing treatment and smolt phenotype. Gonadosomatic index (GSI; %) of juvenile male steelhead sampled at Winthrop National Fish Hatchery in release years 2011–2015 separated according to rearing treatment (S1 in light blue, S2 in violet) and visually determined qualitative smolt phenotype. Data are mean ± SEM. Graph in gray box (F) includes males combined across all release years. Different letters indicate significant differences (p < 0.05) as determined by two-way ANOVA with Tukey’s post-hoc test. Mature males were not included in statistical analyses but are included on the graphs for visual reference. In release years 2014 and 2015, mature male testes were not weighed so no data is shown.
(TIFF)

S16 Fig Plasma 11KT by rearing treatment and smolt phenotype. Plasma 11-ketotestosterone (11KT; ng/ml) of juvenile male steelhead sampled at Winthrop National Fish Hatchery in release years 2011–2015 separated according to rearing treatment (S1 in light blue, S2 in violet) and visually determined qualitative smolt phenotype. Data are mean ± SEM. Graph in gray box (F) includes males combined across all release years. Different letters indicate significant differences (p < 0.05) as determined by two-way ANOVA with Tukey’s post-hoc test. Mature males were not included in statistical analyses but are included on the graphs for visual reference.
(TIFF)

S17 Fig Frequency distribution–body weight. Frequency distributions of S1 (A) and S2 (B) body weight (g) for juvenile steelhead sampled at Winthrop National Fish Hatchery in release years 2011–2015, interleaved colored bars represent individual release years. Solid black lines indicate the density distribution for pooled release years. Dashed black reference lines indicate mode intersection as determined by finite mixture model analysis; however, these values were not used to categorize maturation status or residualism. In release year 2015, there is a single fish that is not represented graphically for the S2 treatment because it is large (in the 260 g bin) and accommodating its presence would reduce the spread of all other data.
(TIFF)

S18 Fig Frequency distribution–gill ATPase activity. Frequency distributions of S1 (A) and S2 (B) gill Na+/K+ ATPase activity (μmol ADP · mg protein^-1^ · hr^-1^) for juvenile steelhead sampled at Winthrop National Fish Hatchery in release years 2012–2015, interleaved colored bars represent individual release years. Solid black lines indicate the density distribution for pooled release years. Dashed black reference lines indicate mode intersection as determined by finite mixture model analysis; however, these values were not used to categorize maturation status or residualism.
(TIFF)

S19 Fig Fork length by rearing treatment and maturation status. Fork length (mm) of juvenile male steelhead sampled at Winthrop National Fish Hatchery in release years 2011–2015 separated according to rearing treatment (S1 in red, S2 in blue) and maturation status as determined by finite mixture model analysis. Data are mean ± SEM. Graph in gray box (F) includes males combined across all release years. An asterisk indicates a significant difference (p < 0.05) between rearing treatments within a maturation category as determined by two-sample t-test. Mature males were not included in statistical analyses but are included on the graphs for visual reference.
(TIFF)

S20 Fig Body weight by rearing treatment and maturation status. Body weight (g) of juvenile male steelhead sampled at Winthrop National Fish Hatchery in release years 2011–2015 separated according to rearing treatment (S1 in red, S2 in blue) and maturation status as determined by finite mixture model analysis. Data are mean ± SEM. Graph in gray box (F) includes males combined across all release years. An asterisk indicates a significant difference (p < 0.05) between rearing treatments within a maturation category as determined by two-sample t-test. Mature males were not included in statistical analyses but are included on the graphs for visual reference.
(TIFF)

S21 Fig Gill ATPase activity by rearing treatment and maturation status. Gill Na+/K+ ATPase activity (μmol ADP · mg protein^-1^ · hr^-1^) of juvenile male steelhead sampled at Winthrop National Fish Hatchery in release years 2012–2015 separated according to rearing treatment (S1 in red, S2 in blue) and maturation status as determined by finite mixture model analysis. Data are mean ± SEM. Graph in gray box (E) includes males combined across all release years. An asterisk indicates a significant difference (p < 0.05) between rearing treatments within a maturation category as determined by two-sample t-test. Mature males were not included in statistical analyses but are included on the graphs for visual reference.
(TIFF)

S22 Fig Pituitary *fshb* by rearing treatment and maturation status. Pituitary follicle stimulating hormone beta-subunit (*fshb*) mRNA relative expression of juvenile male steelhead sampled at Winthrop National Fish Hatchery in release years 2011–2015 separated according to rearing treatment (S1 in red, S2 in blue) and maturation status as determined by finite mixture model analysis. Data are mean ± SEM. An asterisk indicates a significant difference (p < 0.05) between rearing treatments within a maturation category as determined by two-sample t-test. Mature males were not included in statistical analyses but are included on the graphs for visual reference. Graph for pooled release years is contained in Fig 9.
(TIFF)

S23 Fig Pituitary *lhb* by rearing treatment and maturation status. Pituitary luteinizing hormone beta-subunit (*lhb*) mRNA relative expression of juvenile male steelhead sampled at Winthrop National Fish Hatchery in release years 2011–2015 separated according to rearing treatment (S1 in red, S2 in blue) and maturation status as determined by finite mixture model analysis. Data are mean ± SEM. An asterisk indicates a significant difference (p < 0.05) between rearing treatments within a maturation category as determined by two-sample t-test. Mature males were not included in statistical analyses but are included on the graphs for visual reference. Graph for pooled release years is contained in Fig 9.
(TIFF)

S24 Fig Testis *amh* and *igf3* by rearing treatment and maturation status. Testis anti-Mullerian hormone (amh; A-B) and insulin-like growth factor-3 (igf3; C-D) mRNA relative expression of juvenile male steelhead sampled at Winthrop National Fish Hatchery in release years 2011 and 2012 separated according to rearing treatment (S1 in red, S2 in blue) and maturation status as determined by finite mixture model analysis. Data are mean ± SEM. An asterisk indicates a significant difference (p < 0.05) between rearing treatments within a maturation category as determined by two-sample t-test. Measurement of mature (spermiating) males is not possible with our method. Due to logistical constraints, we were unable to measure testis *amh* and *igf3* in all years of the study. Graphs for pooled release years are contained in Fig 9.
(TIFF)

S25 Fig GSI by rearing treatment and maturation status. Gonadosomatic index (GSI; %) of juvenile male steelhead sampled at Winthrop National Fish Hatchery in release years 2011–2015 separated according to rearing treatment (S1 in red, S2 in blue) and maturation status as determined by finite mixture model analysis. Data are mean ± SEM. An asterisk indicates a significant difference (p < 0.05) between rearing treatments within a maturation category as determined by two-sample t-test. Mature males were not included in statistical analyses but are included on the graphs for visual reference. In release years 2014 and 2015, mature male testes were not weighed so no data is shown. Graph for pooled release years is contained in Fig 9.
(TIFF)

S26 Fig Plasma 11KT by rearing treatment and maturation status. Plasma 11-ketotestosterone (11KT; ng/ml) of juvenile male steelhead sampled at Winthrop National Fish Hatchery in release years 2011–2015 separated according to rearing treatment (S1 in red, S2 in blue) and maturation status as determined by finite mixture model analysis. Data are mean ± SEM. An asterisk indicates a significant difference (p < 0.05) between rearing treatments within a maturation category as determined by two-sample t-test. Mature males were not included in statistical analyses but are included on the graphs for visual reference. Graph for pooled release years is contained in Fig 9.
(TIFF)

S1 Table Two-way ANOVA analysis results for rearing treatment and histological stage. Results from two-way ANOVA analysis of juvenile male steelhead sampled at Winthrop National Fish Hatchery in release years 2011–2015 with rearing treatment (S1, S2), histological stage (immature, stage 0; maturing, stage 1–4), and their interaction as factors. Asterisks indicate p-value significance < 0.05, symbol meanings are as follows: “***”, p-value < 0.001; “**”, p-value between 0.001 and 0.01; “*”, p-value between 0.01 and 0.05; “.”, p-value between 0.05 and 0.1; “^”, an interaction term creates aliased coefficients in the model. Abbreviations and units of measure are as follows: fork length (mm); body weight (g); gill Na+/K+ ATPase activity (μmol ADP · mg protein^-1^ · hr^-1^); pituitary follicle stimulating hormone beta-subunit (*fshb*) and luteinizing hormone beta-subunit (*lhb*), testis anti-Mullerian hormone (*amh*) and insulin-like growth factor-3 (*igf3*) (relative mRNA expression); gonadosomatic index (GSI; %); plasma 11-ketotestosterone (11KT; ng/ml).
(XLSX)

S2 Table Sample sizes for morphological and physiological measures by rearing treatment and histological stage. Sample sizes of juvenile male steelhead sampled at Winthrop National Fish Hatchery separated according to rearing treatment (S1, S2), release year (2011–2015), and histological stage (immature, stage 0; maturing, stage 1–4; mature, spermiating). Morphological and physiological measures are represented in S1–S8 Figs with sample sizes corresponding to individual bars. Abbreviations and units of measure are as follows: fork length (mm); body weight (g); gill Na+/K+ ATPase activity (μmol ADP · mg protein^-1^ · hr^-1^); pituitary mRNA (follicle stimulating hormone beta-subunit, *fshb*; luteinizing hormone beta-subunit, *lhb*), testis mRNA (anti-Mullerian hormone, *amh*; insulin-like growth factor-3, *igf3*) (relative mRNA expression); gonadosomatic index (GSI; %); plasma 11-ketotestosterone (11KT; ng/ml).
(XLSX)

S3 Table Morphological measures, gill ATPase activity, and smolt phenotype percentage by sex and rearing treatment. Mean fork length (mm), body weight (g), and gill Na+/K+ ATPase activity (μmol ADP · mg protein^-1^ · hr^-1^) of juvenile steelhead sampled at Winthrop National Fish Hatchery in release years 2011–2015 separated according to sex and rearing treatment. Data are mean ± SEM. Sample sizes represent total fish measured for fork length and weight [N (Total)] or the subset of fish also measured for gill ATPase activity [N (ATPase)]. Smolt phenotypes (parr, transitional, smolt, mature male) were determined visually and are represented as a percentage of N (Total); these percentages were used to create Fig 4.
(XLSX)

S4 Table Male maturation measures by rearing treatment. Mean pituitary mRNA (follicle stimulating hormone beta-subunit, *fshb*; luteinizing hormone beta-subunit, *lhb*), testis mRNA (anti-Mullerian hormone, *amh*; insulin-like growth factor-3, *igf3*) (relative mRNA expression); gonadosomatic index (GSI; %); and plasma 11-ketotestosterone (11KT; ng/ml) of juvenile male steelhead sampled at Winthrop National Fish Hatchery in release years 2011–2015 separated according to rearing treatment. Data are mean ± SEM. Sample sizes represent total males analyzed for each measure as noted in parentheses. Due to logistical constraints, we were unable to measure testis *amh* and *igf3* in all years of the study. GSI is not included for release year 2014 and 2015 because mature male testes were not weighed.
(XLSX)

S5 Table Maturation status and predicted residuals by rearing treatment and sex. Sample sizes and percentage of juvenile steelhead sampled at Winthrop National Fish Hatchery in release years 2011–2015 separated according to rearing treatment and sex for maturation status (immature, maturing, mature) and predicted residualism. Categorization of maturity is based on physiological measures (pituitary mRNA expression, follicle stimulating hormone beta-subunit (*fshb*), luteinizing hormone beta subunit (*lhb*); testis mRNA expression, anti-Mullerian hormone (*amh*), insulin-like growth factor-3 (*igf3*); gonadosomatic index, GSI; and plasma 11-ketotestosterone, 11KT) and their threshold values determined by finite mixture model analysis as described in the Methods section. Categorization of residuals is based on a combination of maturation status and fork length threshold determined by finite mixture model analysis as described in the Methods. Percentages are represented as a portion of total sample size within a release year for a given rearing treatment (S1, S2) and sex (male, female, all fish); these percentages were used to create Figs 8 and 10 (values in bold).
(XLSX)

S6 Table Two-way ANOVA analysis for rearing treatment and smolt phenotype–male. Results from two-way ANOVA analysis of juvenile male steelhead sampled at Winthrop National Fish Hatchery in release years 2011–2015 with rearing treatment (S1, S2), visually determined qualitative smolt phenotype (parr, transitional, smolt), and their interaction as factors. Asterisks indicate p-value significance < 0.05, symbol meanings are as follows: “***”, p-value < 0.001; “**”, p-value between 0.001 and 0.01; “*”, p-value between 0.01 and 0.05; “.”, p-value between 0.05 and 0.1. Abbreviations and units of measure are as follows: fork length (mm); body weight (g); gill Na+/K+ ATPase activity (μmol ADP · mg protein^-1^ · hr^-1^); pituitary follicle stimulating hormone beta-subunit (*fshb*) and luteinizing hormone beta-subunit (*lhb*), testis anti-Mullerian hormone (*amh*) and insulin-like growth factor-3 (*igf3*) (relative mRNA expression); gonadosomatic index (GSI; %); plasma 11-ketotestosterone (11KT; ng/ml). Results for fork length, body weight and gill ATPase activity for all years combined are contained in Table 2.
(XLSX)

S7 Table Two-way ANOVA analysis for rearing treatment and smolt phenotype–female. Results from two-way ANOVA analysis of juvenile female steelhead sampled at Winthrop National Fish Hatchery in release years 2011–2015 with rearing treatment (S1, S2), visually determined qualitative smolt phenotype (parr, transitional, smolt), and their interaction as factors. Asterisks indicate p-value significance < 0.05, symbol meanings are as follows: “***”, p-value < 0.001; “**”, p-value between 0.001 and 0.01; “*”, p-value between 0.01 and 0.05; “.”, p-value between 0.05 and 0.1; “^”, an interaction term creates aliased coefficients in the model. Abbreviations and units of measure are as follows: fork length (mm), body weight (g), gill Na+/K+ ATPase activity (μmol ADP · mg protein^-1^ · hr^-1^). Results for all years combined are contained in Table 2.
(XLSX)

S8 Table Sample sizes for morphological and physiological measures by rearing treatment and smolt phenotype—male. Sample sizes of juvenile male steelhead sampled at Winthrop National Fish Hatchery separated according to rearing treatment (S1, S2), release year (2011–2015), and visually determined qualitative smolt phenotype (parr, transitional, smolt, mature male). Morphological and physiological measures are represented in Fig 3 and S9–S16 Figs with sample sizes corresponding to individual bars. Abbreviations and units of measure are as follows: fork length (mm); body weight (g); gill Na+/K+ ATPase activity (μmol ADP · mg protein^-1^ · hr^-1^); pituitary mRNA (follicle stimulating hormone beta-subunit, *fshb*; luteinizing hormone beta-subunit, *lhb*), testis mRNA (anti-Mullerian hormone, *amh*; insulin-like growth factor-3, *igf3*) (relative mRNA expression); gonadosomatic index (GSI; %); plasma 11-ketotestosterone (11KT; ng/ml).
(XLSX)

S9 Table Sample sizes for morphological measures and gill ATPase activity by rearing treatment and smolt phenotype—female. Sample sizes of juvenile female steelhead sampled at Winthrop National Fish Hatchery separated according to rearing treatment (S1, S2), release year (2011–2015), and visually determined qualitative smolt phenotype (parr, transitional, smolt). Morphological measures and gill ATPase activity are represented in Fig 3 and S9–S11 Figs with sample sizes corresponding to individual bars. Abbreviations and units of measure are as follows: fork length (mm); body weight (g); gill Na+/K+ ATPase activity (μmol ADP · mg protein^-1^ · hr^-1^).
(XLSX)

S10 Table Two-sample t-test analysis results comparing rearing treatments within a maturation category. Results from two-sample t-tests of juvenile male steelhead sampled at Winthrop National Fish Hatchery in release years 2011–2015 comparing morphological and physiological measures between rearing treatments within a maturation category. P-values < 0.05 were considered statistically significant. Abbreviations and units of measure are as follows: fork length (mm); body weight (g); gill Na+/K+ ATPase activity (μmol ADP · mg protein^-1^ · hr^-1^); pituitary follicle stimulating hormone beta-subunit (*fshb*) and luteinizing hormone beta-subunit (*lhb*), testis anti-Mullerian hormone (*amh*) and insulin-like growth factor-3 (*igf3*) (relative mRNA expression); gonadosomatic index (GSI; %); plasma 11-ketotestosterone (11KT; ng/ml). Results for *fshb*, *lhb*, *amh*, *igf3*, GSI, and plasma 11KT for all years combined are contained in Table 4.
(XLSX)

S11 Table Sample sizes for morphological and physiological measures by rearing treatment and maturation status. Sample sizes of juvenile male steelhead sampled at Winthrop National Fish Hatchery separated according to rearing treatment (S1, S2), release year (2011–2015), and maturation status as determined by finite mixture model analysis (immature, maturing) or visual identification (mature). Morphological and physiological measures are represented in Fig 9 and S19–S26 Figs with sample sizes corresponding to individual bars. Abbreviations and units of measure are as follows: fork length (mm); body weight (g); gill Na+/K+ ATPase activity (μmol ADP · mg protein^-1^ · hr^-1^); pituitary mRNA (follicle stimulating hormone beta-subunit, *fshb*; luteinizing hormone beta-subunit, *lhb*), testis mRNA (anti-Mullerian hormone, *amh*; insulin-like growth factor-3, *igf3*) (relative mRNA expression); gonadosomatic index (GSI; %); plasma 11-ketotestosterone (11KT; ng/ml).
(XLSX)
