# Supplementary material for: Age at release affects developmental physiology and sex-specific phenotypic diversity of hatchery steelhead trout (Oncorhynchus mykiss)
Source: PLoS One. 2025 Feb 13;20(2):e0315016. doi: 10.1371/journal.pone.0315016 (PMC11825032; doi:10.1371/journal.pone.0315016)
Supplement: S1 File — Methods and results for testis histology staging of juvenile male steelhead sampled at Winthrop National Fish Hatchery in release years 2011–2015. (DOCX) [file pone.0315016.s001.docx]

**Testis histology**

**Methods**

Testes were preserved in Histochoice (AMRESCO, Solon, OH) in release year 2011 and Davidson’s fixative in release years 2012-2015. Histological samples remained in fixative for seven days before transfer to 70% ethanol for long-term storage at room temperature. Histological analysis of stage of spermatogenesis was performed on all testis samples with a GSI greater than 0.040 to identify fish that had initiated puberty. Fish with a GSI less than 0.040 were assumed to be immature (stage 0) based on previous research [48, 49]. Fixed testes were processed through a graded series of ethanol and xylene substitute followed by embedding in paraffin. Sections were cut at 5 microns on a standard rotary microtome and stained with hematoxylin and eosin. Stages of spermatogenesis were semi-quantitatively determined, similar to methods previously described [48, 49]. Stages of spermatogenesis were then grouped into categories of maturation; immature, maturing, and mature. Briefly, categories of maturation in relation to stage of spermatogenesis were defined as follows: immature, stage 0, nearly 100% type A undifferentiated spermatogonia with a small amount of type A differentiated spermatogonia; maturing, stage 1-4, no less than 50% type B spermatogonia or presence of any meiotic germ cell stage without milt production; mature, stage 5, milt production.

**Results**

In release year 2012, we found a single male representing an “attempted maturation” phenotype, which is categorized by mostly type B spermatogonia with a small amount of milt production. Approximately one-third of all two-way ANOVA interactions for treatment and maturation status based on histological stage were statistically significant (p < 0.05) when separated by year (S1 Table). Within treatments, immature and maturing fork lengths were not significantly different (S1 Fig). Within a maturation category, S2 fish either had significantly larger fork length than S1 fish or there was no statistical difference (S1 Fig). Within treatments, immature and maturing body weight was not significantly different, with the exception that in release year 2015 body weight for S2 maturing fish was larger than for S2 immature fish (S2 Fig). Within a maturation category, S2 fish either had significantly larger body weight than S1 fish or there was no statistical difference (S2 Fig). Within treatments, gill ATPase activity for immature and maturing fish was not significantly different (S3 Fig). Within a maturation category, there was no difference between S1 and S2 fish except in release year 2014 where S1 immature fish had lower gill ATPase activity than S2 immature fish and in release year 2015 where S1 immature fish had higher gill ATPase activity than S2 immature fish (S3 Fig). Pituitary *fshb* was higher for maturing fish than immature fish, regardless of treatment, with the exception that in release year 2015, S1 maturing fish were not statistically different from S1 immature fish (S4 Fig). Within treatments, pituitary *lhb* exhibited a mostly statistically significant trend of higher transcript levels in maturing fish than immature fish (S5 Fig). Testis *amh* was higher for immature fish than maturing fish within treatments and *igf3* was lower for immature fish than maturing fish within treatments (S6 Fig). GSI was higher in maturing fish than immature fish within treatments and there was almost no statistical difference in treatments within a maturation category except in release year 2013 immature fish, release year 2015 maturing fish, and maturing fish for all years combined where S2 fish had higher GSI than S1 fish (S7 Fig). Plasma 11KT was higher for maturing fish than immature fish within treatments, except in release year 2014 S2 and release year 2015 S1 when immature and maturing fish were comparable (S8 Fig). Comparison of treatments within a maturation category varied for 11KT, but where there was a statistical difference S1 fish had lower 11KT than S2 fish (S8 Fig). Total sample sizes for each bar in S1-S8 Figs are contained in S2 Table.
