# Supplementary material for: Age at release affects developmental physiology and sex-specific phenotypic diversity of hatchery steelhead trout (Oncorhynchus mykiss)
Source: PLoS One. 2025 Feb 13;20(2):e0315016. doi: 10.1371/journal.pone.0315016 (PMC11825032; doi:10.1371/journal.pone.0315016)
Supplement: S2 File — Results for juvenile steelhead sampled at Winthrop National Fish Hatchery in individual release years. Results for maturation measures (male only) separated according to rearing treatment (S1, S2) and visually determined qualitative smolt phenotype (parr, transitional, smolt, mature male). Results for finite mixture model (fmm) analysis of body weight and gill Na+/K+ ATPase activity. Morphological measures separated according to rearing treatment (S1, S2) and maturation status as determined by fmm analysis (immature, maturing, mature). (DOCX) [file pone.0315016.s002.docx]

**Supplementary results**

Fork length, body weight and ATPase (mean ± SEM), total sample sizes for each measure, and proportion of each visually identified smolt phenotype were separated by year, treatment, and sex (S3 Table). Physiological measures for males only (mean ± SEM) and total sample sizes for each measure were separated by year and treatment (S4 Table). Total sample sizes and proportions used to create Fig 8 and Fig 10 are contained in S5 Table.

Approximately 20% of the two-way ANOVA analyses conducted for individual release years, or combined release years for male maturation markers, revealed statistically significant interactions between the effects of treatment and smolt phenotype (S6-S7 Tables). In all release years, fish from both rearing treatments increased in fork length (S9 Fig) and body weight (S10 Fig) from the parr to transitional to smolt phenotypes. For the most part, gill ATPase activity demonstrated an increase from parr to the transitional or smolt phenotypes with rearing treatments, however, the pattern of increase (parr to transitional or parr to smolt) was not consistent across years (S11 Fig). Male pituitary *fshb* was comparable across smolt phenotypes and treatments within release years, but when all release years were combined, *fshb* decreased from the parr to the transitional phenotype (S12 Fig). There was a further decrease in *fshb* from the transitional to the smolt phenotype for the S1 males while the S2 males were not significantly different between transitional and smolts (S12 Fig). Male pituitary *lhb* was mostly comparable across smolt phenotypes and treatments in individual release years and after release years were combined (S13 Fig). However, in release year 2014 the S1 rearing treatment exhibited a statistically significant decrease in *lhb* from the parr to transitional to smolt phenotypes and the S1 males had higher *lhb* relative expression than S2 males for all smolt phenotypes (S13 Fig). Testis *amh* and *igf3* was mostly comparable across smolt phenotypes and treatments for individual release years and with all release years combined. However, *igf3* relative expression from the S2 parr was significantly higher than all other smolt phenotypes and treatments (S14 Fig). For the most part, GSI was higher in the parr phenotype than either the transitional or smolt phenotype across release years and treatments; however, in release year 2014 only S1 parr were statistically different and in release year 2015 there were no statistical difference regardless of treatment or smolt phenotype (S15 Fig). Plasma 11KT was generally higher in parr while the transitional and smolt phenotypes were comparable within each rearing treatment (S16 Fig). Total sample sizes for each bar in Fig 3 and S9-S16 Figs are contained in S8 Table for males. Total sample sizes for each bar in Fig 3 and S9-11 Figs are contained in S9 Table for females.

Frequency distributions for both S1 and S2 body weights were trimodal (S1: fmm1 BIC = 28585.23, fmm2 BIC = 28215.02, fmm3 BIC = **28209.77**; S2: fmm1 BIC = 28413.03, fmm2 BIC = 28297.31, fmm3 BIC = **28251.13**) (S17 Fig). For S1 fish, the first and second modes intersect at 26.8 g and the second and third modes intersect at 122.4 g. For S2 fish, the first and second modes intersect at 30.9 g and the second and third modes intersect at 110.4 g. For gill ATPase activity, the S1 treatment was bimodal (fmm1 BIC = 2613.34, fmm2 BIC = **2494.36**), while the S2 treatment was trimodal (fmm 1 BIC = 2478.05, fmm2 BIC = 2353.25, fmm3 BIC = **2348.87**) (S18 Fig). For S1 fish, the first and second modes intersect at 4.02 µmol ADP · mg protein^-1^ · hr^-1^; for S2 fish the first and second modes intersect at 3.38 µmol ADP · mg protein^-1^ · hr^-1^ and the second and third modes intersect at 6.22 µmol ADP · mg protein^-1^ · hr^-1^. Neither body weight nor ATPase were used to categorize maturation status or residual/migrant status.

Results of two-sample t-tests comparing rearing treatments within a maturation category (immature and maturing only) are contained in S10 Table. S1 and S2 immature males had significantly different fork length and body weight in all individual release years, and combined release years, with the exception of release year 2013 (S19-20 Fig). S1 and S2 maturing males had significantly different fork lengths and body weights in release years 2011, 2012, 2015, and when all years were combined (S19-20 Fig). S1 and S2 immature males had significantly different gill ATPase activity in all individual release years, and combined release years, with the exception of release year 2012 (S21 Fig). S1 and S2 maturing males did not have significantly different gill ATPase activity except in release year 2015 (S21 Fig). For pituitary *fshb*, S1 and S2 immature and maturing males were different in all release years except for release years 2012-2014 where maturing fish were not different between treatments (S22 Fig). S1 and S2 immature males had significantly different pituitary *lhb* in all release years, except release year 2012 (S23 Fig). S1 and S2 maturing males did not have significantly different pituitary *lhb* in release years 2011 and 2013, but did have significantly different *lhb* in release years 2012, 2014, and 2015 (S23 Fig). Testis *amh* and *igf3* were significantly different between rearing treatments for both immature and maturing males, except in release year 2011 where testis *igf3* was not different between treatments for maturing males (S24 Fig). Immature males had significantly different GSI between the S1 and S2 rearing treatments in release years 2013-2015, but rearing treatments were not significantly different in release years 2011-2012 (S25 Fig). Maturing males only had significantly different GSI between rearing treatments in release year 2013 (S25 Fig). Immature males had significantly different plasma 11KT between the S1 and S2 rearing treatments in release years 2012-2014, but rearing treatments were not significantly different in release years 2011 and 2015 (S26 Fig). Maturing males had significantly different plasma 11KT between rearing treatments in all release years except release year 2011 (S26 Fig). Total sample sizes for each bar in Figs 9 and S19-S26 Figs are contained in S11 Table.
