# Supplementary figures and images for: Age at release affects developmental physiology and sex-specific phenotypic diversity of hatchery steelhead trout (Oncorhynchus mykiss)
Source: PLoS One. 2025 Feb 13;20(2):e0315016. doi: 10.1371/journal.pone.0315016 (PMC11825032; doi:10.1371/journal.pone.0315016)

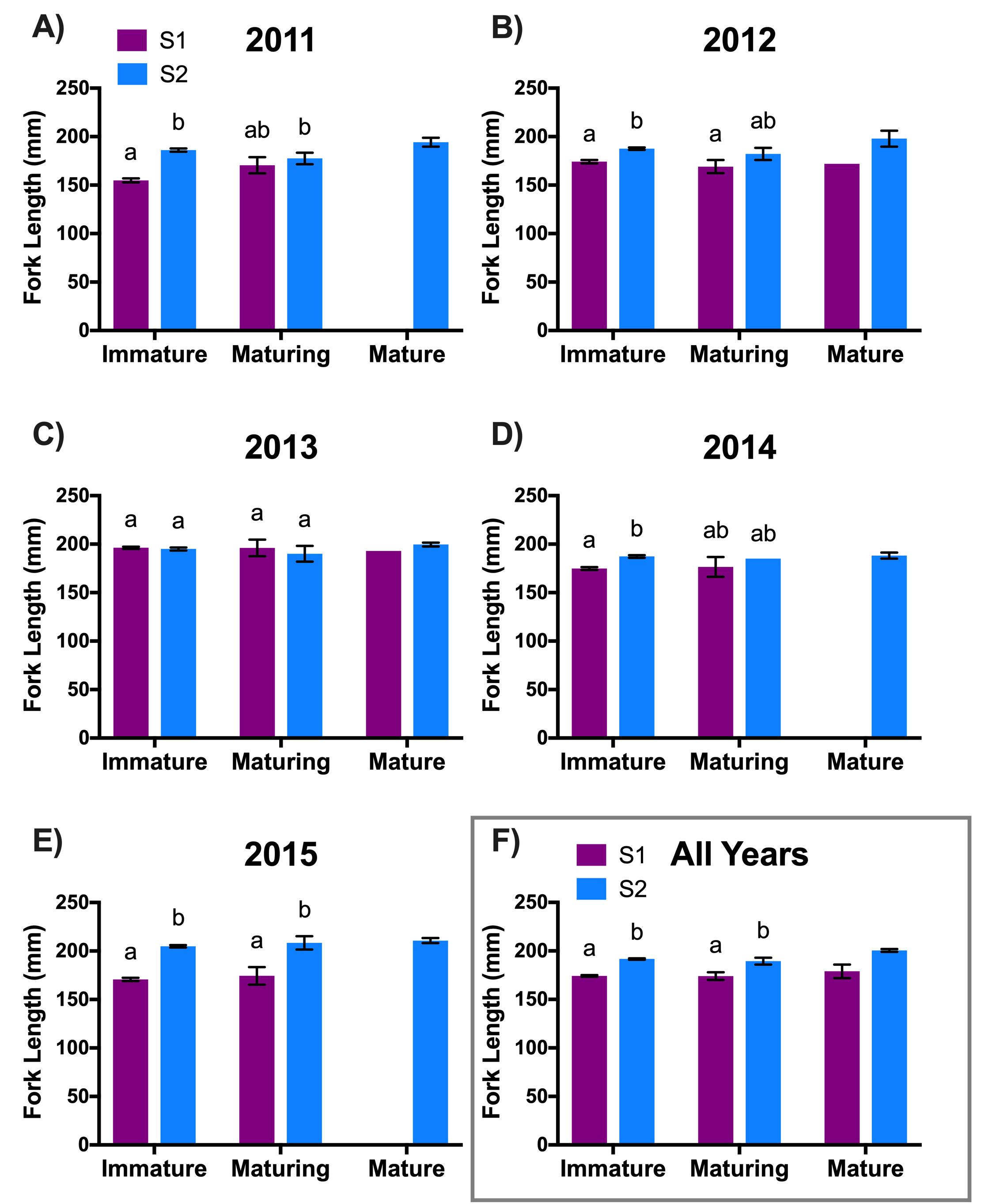

Supplement: S1 Fig — Fork length (mm) of juvenile male steelhead sampled at Winthrop National Fish Hatchery in release years 2011–2015 separated according to rearing treatment (S1 in purple, S2 in blue) and histological stage (immature, stage 0; maturing, stage 1–4; mature, spermiating). Data are mean ± SEM. Graph in box (F) includes males combined across all release years. Different letters indicate significant differences (p < 0.05) as determined by two-way ANOVA with Tukey’s post-hoc test. Mature males were not included in statistical analyses but are included on the graphs for visual reference. (TIFF) [file pone.0315016.s003.tiff]

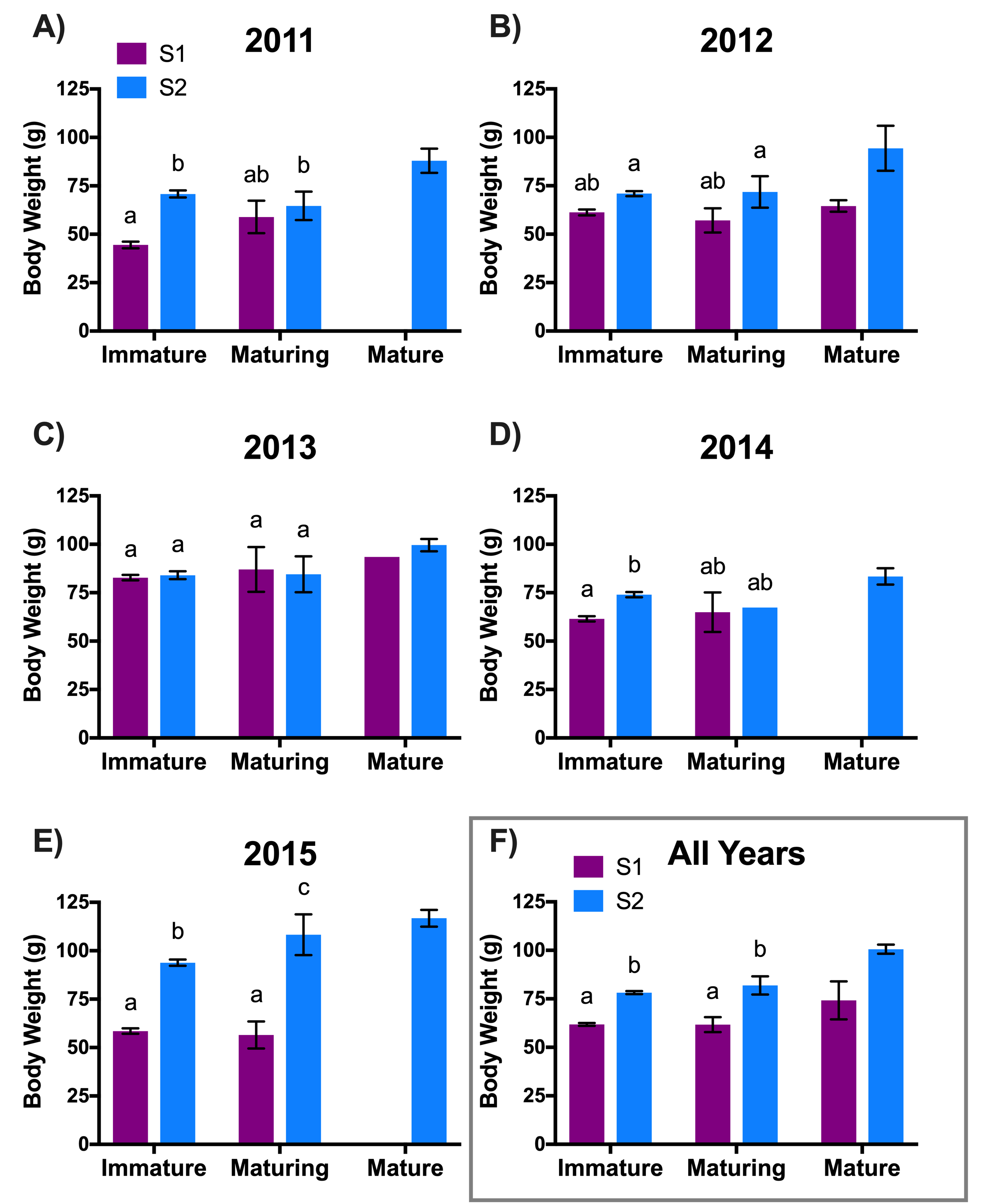

Supplement: S2 Fig — Body weight (g) of juvenile male steelhead sampled at Winthrop National Fish Hatchery in release years 2011–2015 separated according to rearing treatment (S1 in purple, S2 in blue) and histological stage (immature, stage 0; maturing, stage 1–4; mature, spermiating). Data are mean ± SEM. Graph in box (F) includes males combined across all release years. Different letters indicate significant differences (p < 0.05) as determined by two-way ANOVA with Tukey’s post-hoc test. Mature males were not included in statistical analyses but are included on the graphs for visual reference. (TIFF) [file pone.0315016.s004.tiff]

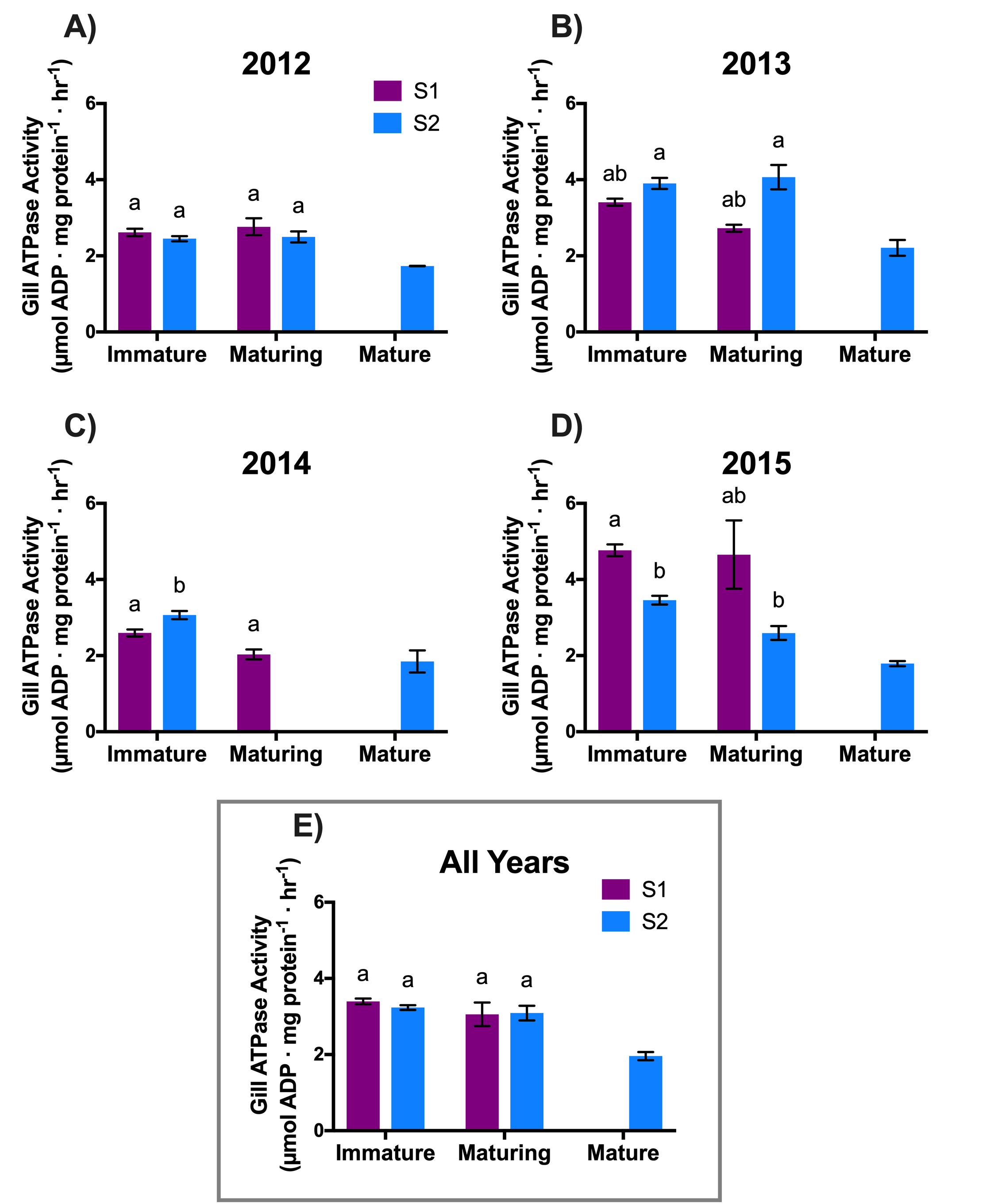

Supplement: S3 Fig — Gill Na+/K+ ATPase activity (μmol ADP · mg protein-1 · hr-1) of juvenile male steelhead sampled at Winthrop National Fish Hatchery in release years 2012–2015 separated according to rearing treatment (S1 in purple, S2 in blue) and histological stage (immature, stage 0; maturing, stage 1–4; mature, spermiating). Data are mean ± SEM. Graph in box (E) includes males combined across all release years. Different letters indicate significant differences (p < 0.05) as determined by two-way ANOVA with Tukey’s post-hoc test. Mature males were not included in statistical analyses but are included on the graphs for visual reference. (TIFF) [file pone.0315016.s005.tiff]

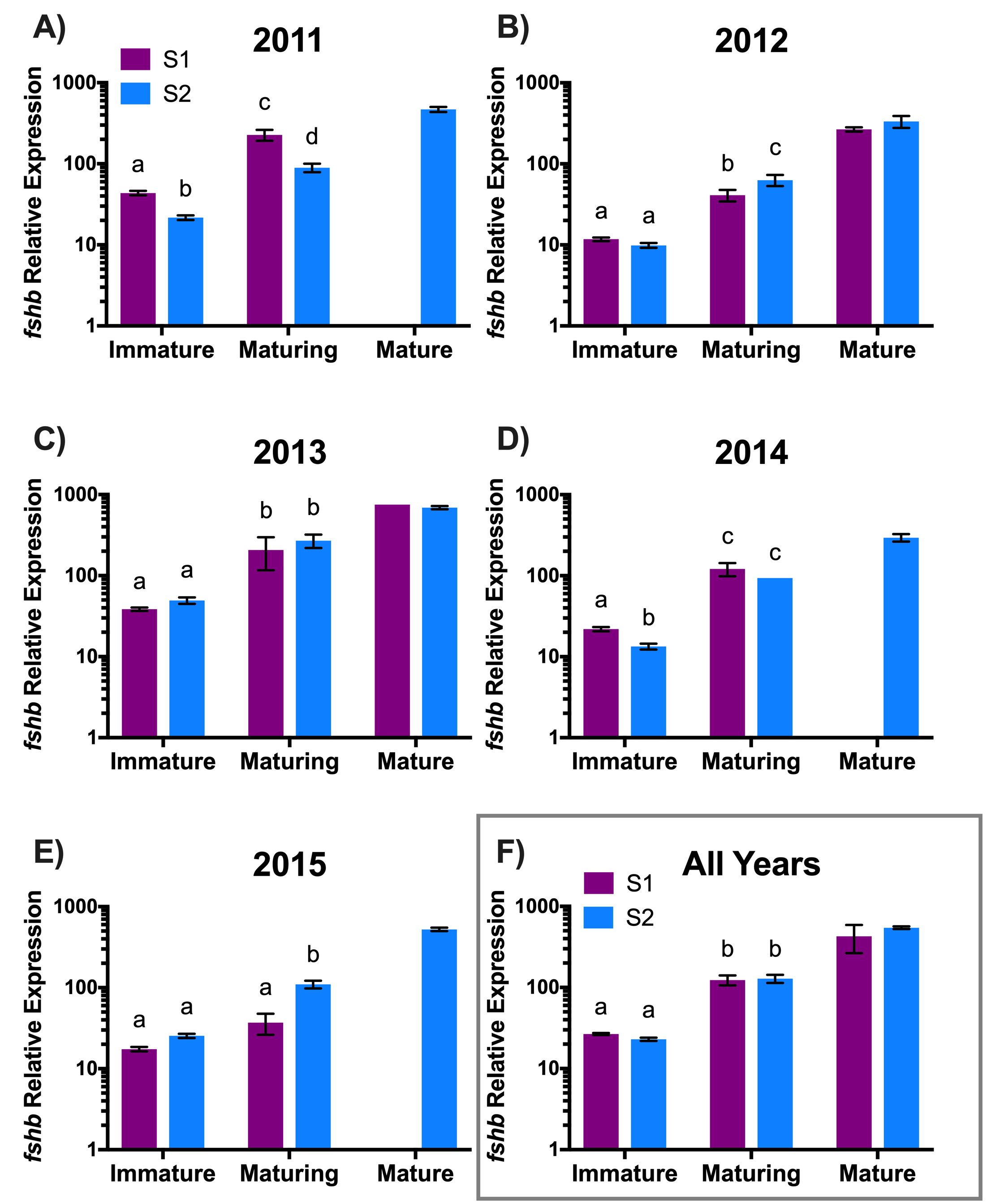

Supplement: S4 Fig — Pituitary follicle stimulating hormone beta-subunit (fshb) mRNA relative expression of juvenile male steelhead sampled at Winthrop National Fish Hatchery in release years 2011–2015 separated according to rearing treatment (S1 in purple, S2 in blue) and histological stage (immature, stage 0; maturing, stage 1–4; mature, spermiating). Data are mean ± SEM. Graph in box (F) includes males combined across all release years. Different letters indicate significant differences (p < 0.05) as determined by two-way ANOVA with Tukey’s post-hoc test. Mature males were not included in statistical analyses but are included on the graphs for visual reference. (TIFF) [file pone.0315016.s006.tiff]

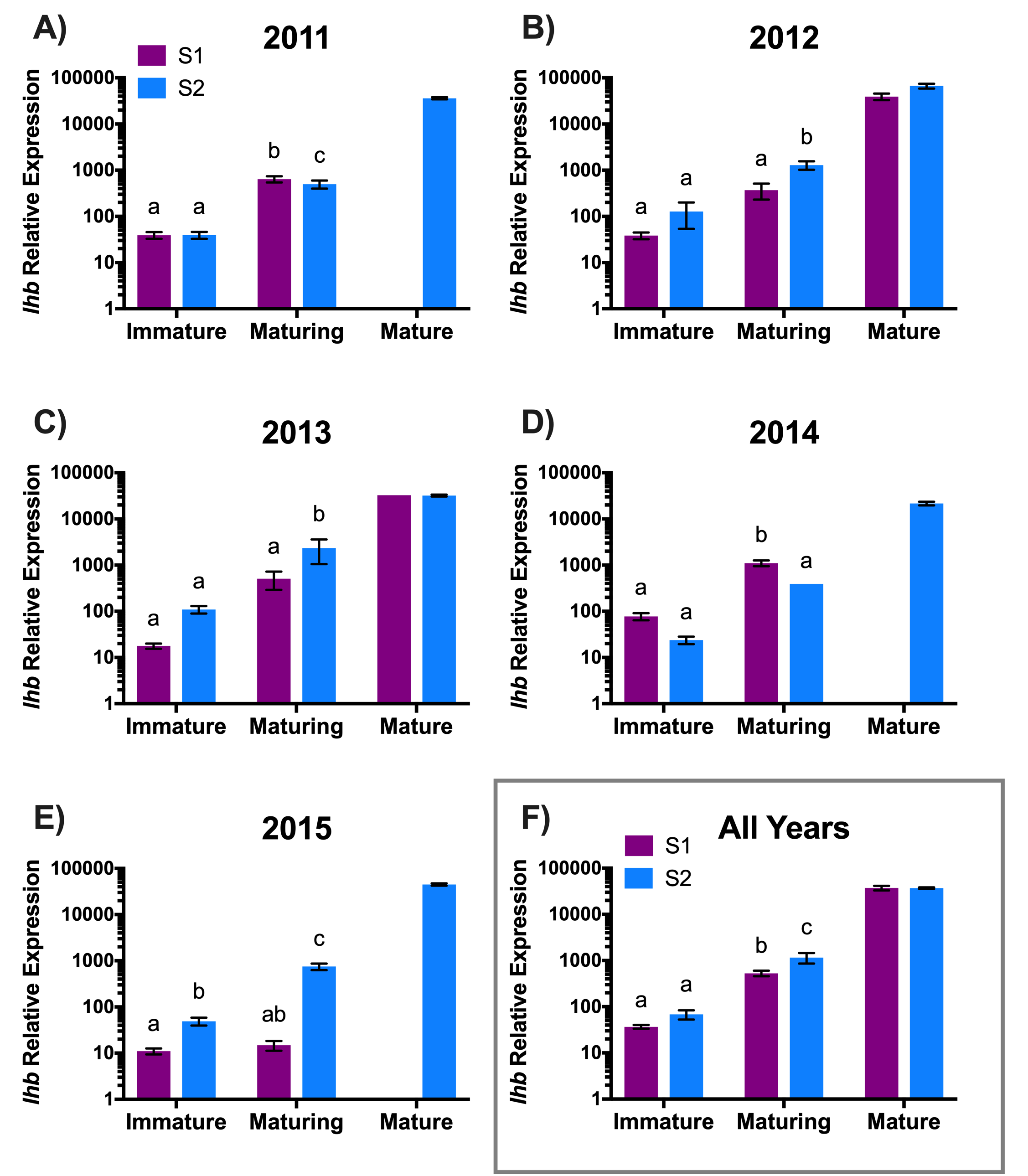

Supplement: S5 Fig — Pituitary luteinizing hormone beta-subunit (lhb) mRNA relative expression of juvenile male steelhead sampled at Winthrop National Fish Hatchery in release years 2011–2015 separated according to rearing treatment (S1 in purple, S2 in blue) and histological stage (immature, stage 0; maturing, stage 1–4; mature, spermiating). Data are mean ± SEM. Graph in box (F) includes males combined across all release years. Different letters indicate significant differences (p < 0.05) as determined by two-way ANOVA with Tukey’s post-hoc test. Mature males were not included in statistical analyses but are included on the graphs for visual reference. (TIFF) [file pone.0315016.s007.tiff]

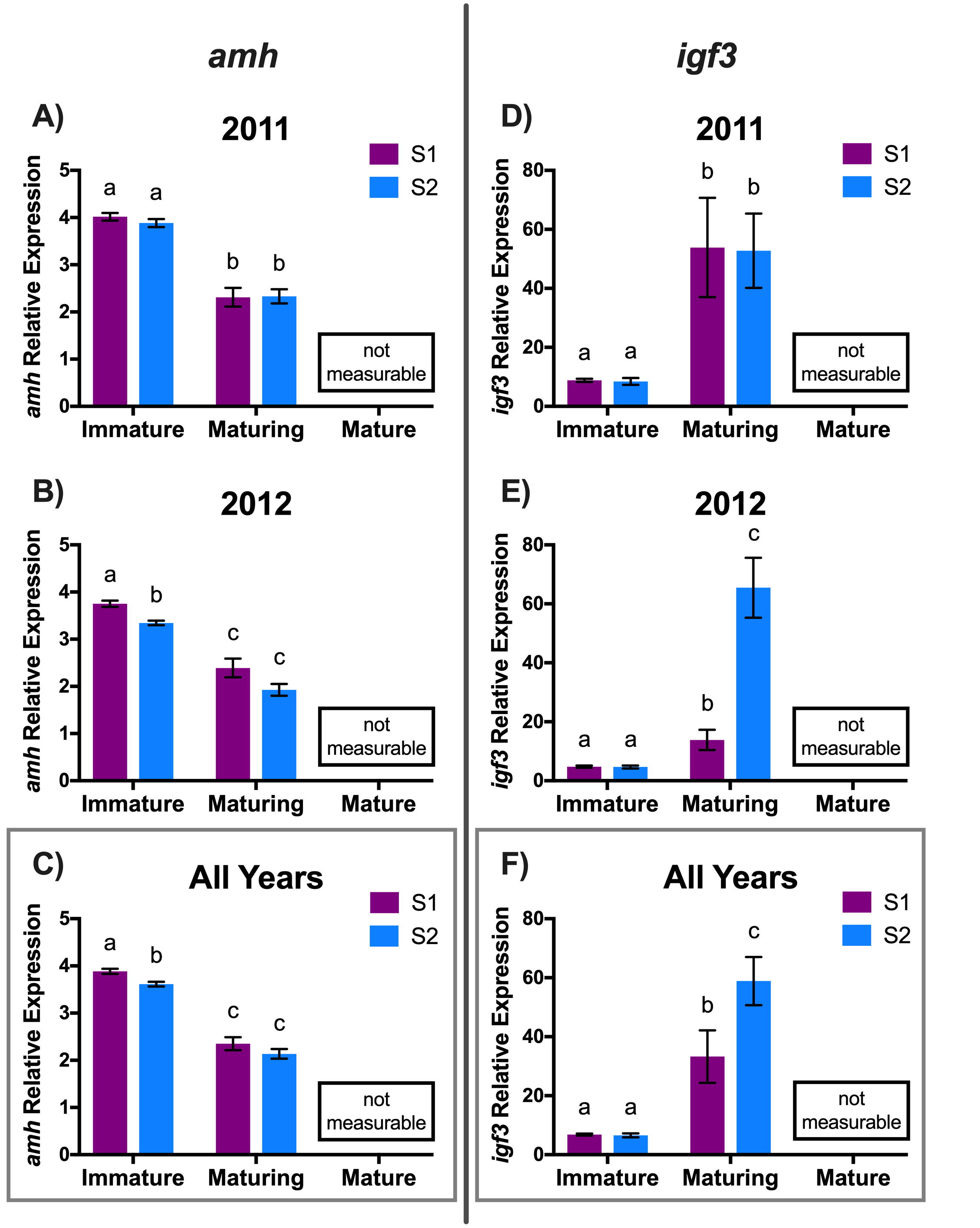

Supplement: S6 Fig — Testis anti-Mullerian hormone (amh; A-C) and insulin-like growth factor-3 (igf3; D-F) mRNA relative expression of juvenile male steelhead sampled at Winthrop National Fish Hatchery in release years 2011 and 2012 separated according to rearing treatment (S1 in purple, S2 in blue) and histological stage (immature, stage 0; maturing, stage 1–4; mature, spermiating). Data are mean ± SEM. Graphs in boxes (C and F) include males combined across all release years. Different letters indicate significant differences (p < 0.05) as determined by two-way ANOVA with Tukey’s post-hoc test. Measurement of mature (spermiating) males is not possible with our method. Due to logistical constraints we were unable to measure testis amh and igf3 in all years of the study. (TIFF) [file pone.0315016.s008.tiff]

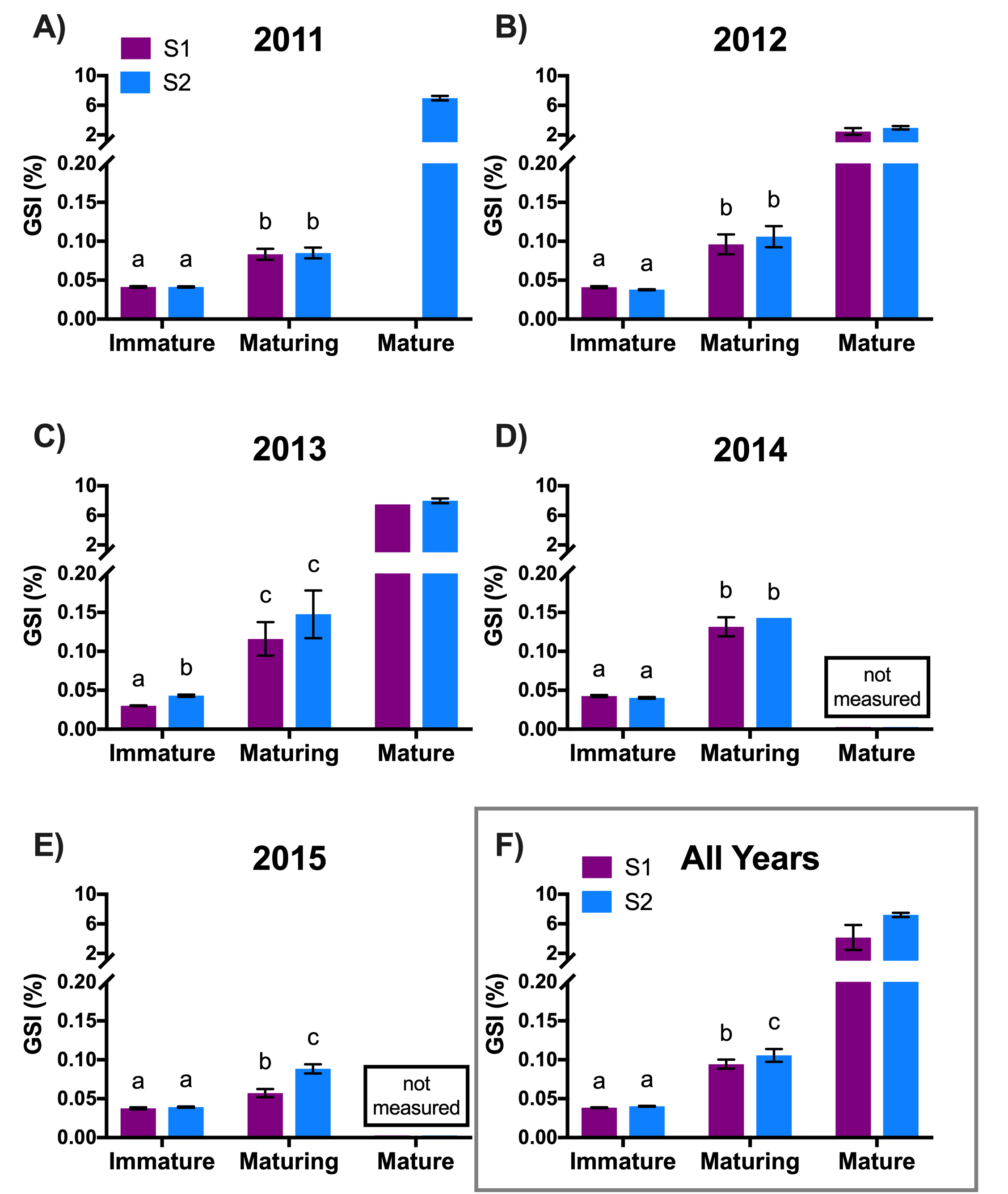

Supplement: S7 Fig — Gonadosomatic index (GSI; %) of juvenile male steelhead sampled at Winthrop National Fish Hatchery in release years 2011–2015 separated according to rearing treatment (S1 in purple, S2 in blue) and histological stage (immature, stage 0; maturing, stage 1–4; mature, spermiating). Data are mean ± SEM. Graph in box (F) includes males combined across all release years. Different letters indicate significant differences (p < 0.05) as determined by two-way ANOVA with Tukey’s post-hoc test. Mature males were not included in statistical analyses but are included on the graphs for visual reference. In release years 2014 and 2015, mature male testes were not weighed so no data is shown. (TIFF) [file pone.0315016.s009.tiff]

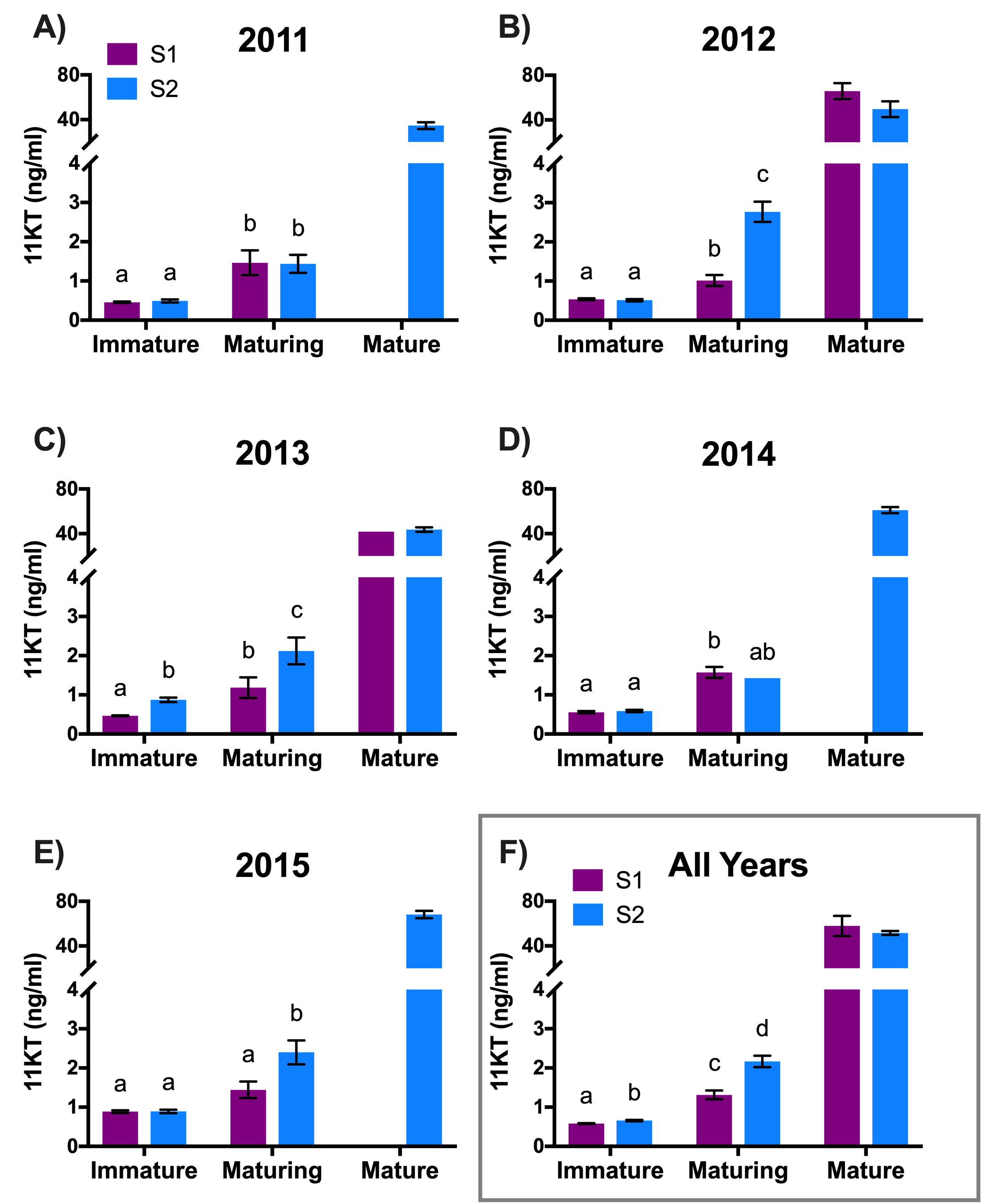

Supplement: S8 Fig — Plasma 11-ketotestosterone (11KT; ng/ml) of juvenile male steelhead sampled at Winthrop National Fish Hatchery in release years 2011–2015 separated according to rearing treatment (S1 in purple, S2 in blue) and histological stage (immature, stage 0; maturing, stage 1–4; mature, spermiating). Data are mean ± SEM. Graph in box (F) includes males combined across all release years. Different letters indicate significant differences (p < 0.05) as determined by two-way ANOVA with Tukey’s post-hoc test. Mature males were not included in statistical analyses but are included on the graphs for visual reference. (TIFF) [file pone.0315016.s010.tiff]

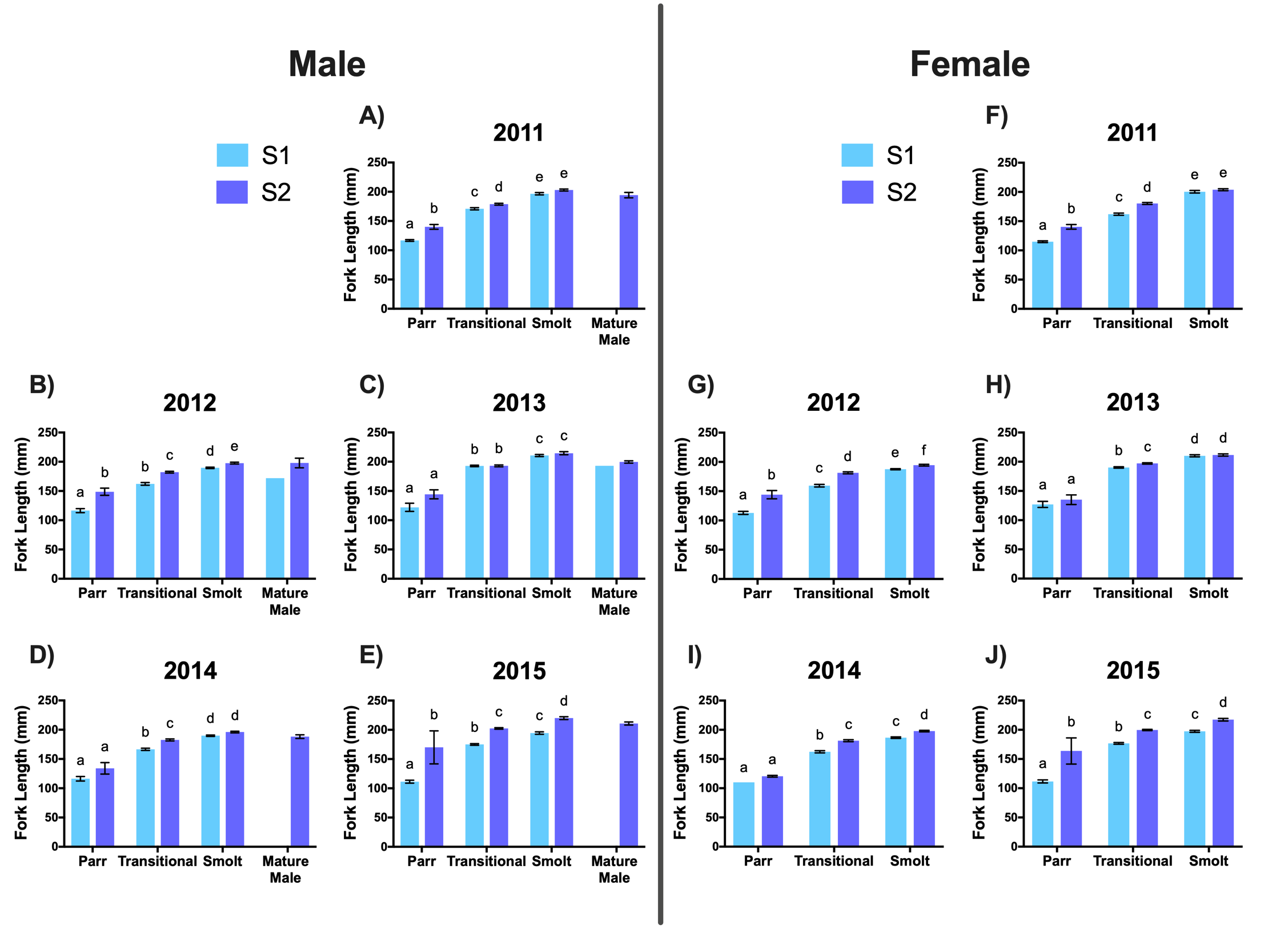

Supplement: S9 Fig — Fork length (mm) of juvenile steelhead sampled at Winthrop National Fish Hatchery in release years 2011–2015 separated according to rearing treatment (S1 in light blue, S2 in violet) and visually determined qualitative smolt phenotype for males (A-E) and females (F-J). Data are mean ± SEM. Different letters indicate significant differences (p < 0.05) as determined by two-way ANOVA with Tukey’s post-hoc test. Mature males were not included in statistical analyses but are included on the graphs for visual reference. Graphs for pooled release years are contained in Fig 3. (TIFF) [file pone.0315016.s011.tiff]

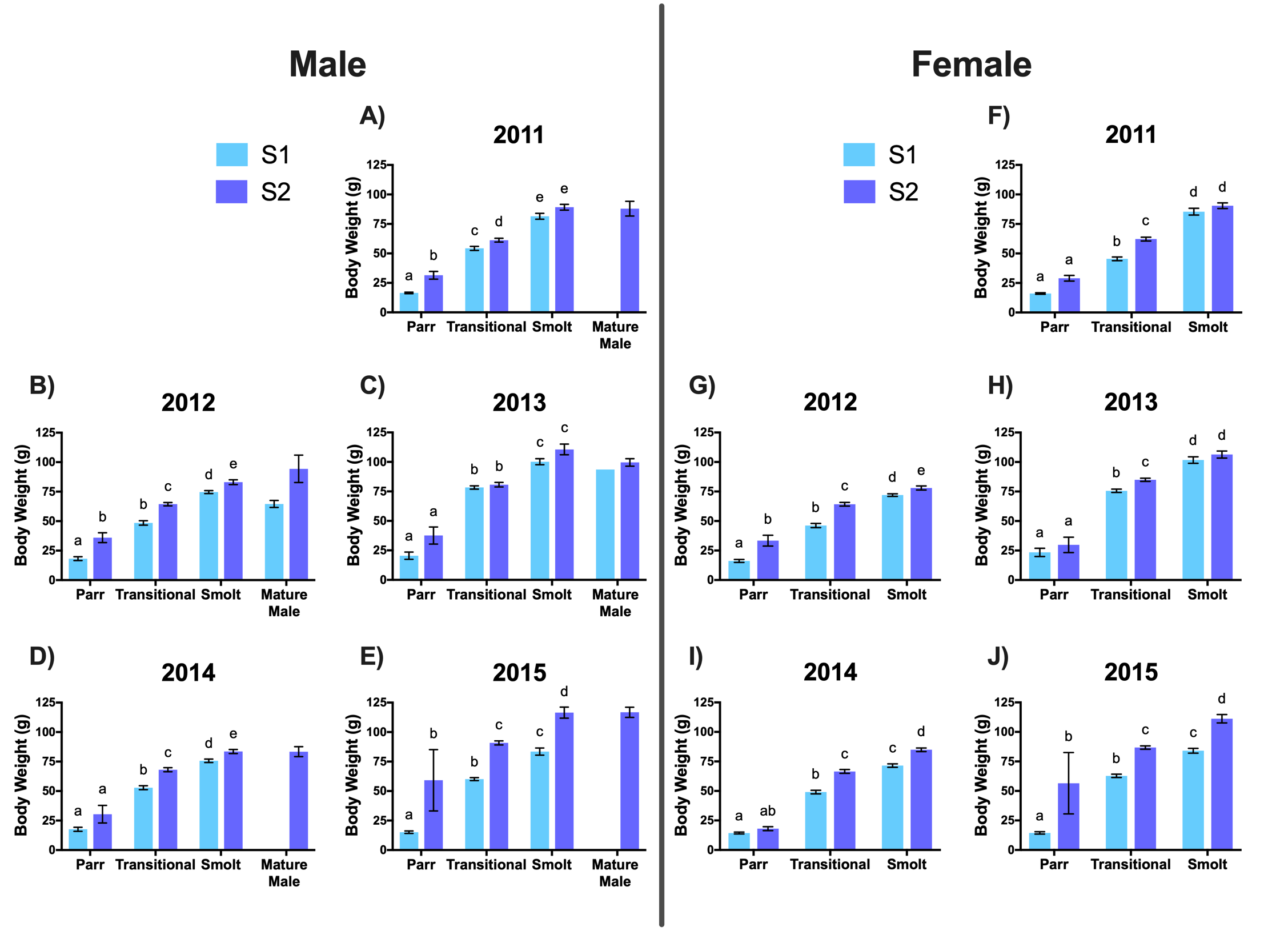

Supplement: S10 Fig — Body weight (g) of juvenile steelhead sampled at Winthrop National Fish Hatchery in release years 2011–2015 separated according to rearing treatment (S1 in light blue, S2 in violet) and visually determined qualitative smolt phenotype for males (A-E) and females (F-J). Data are mean ± SEM. Different letters indicate significant differences (p < 0.05) as determined by two-way ANOVA with Tukey’s post-hoc test. Mature males were not included in statistical analyses but are included on the graphs for visual reference. Graphs for pooled release years are contained in Fig 3. (TIFF) [file pone.0315016.s012.tiff]

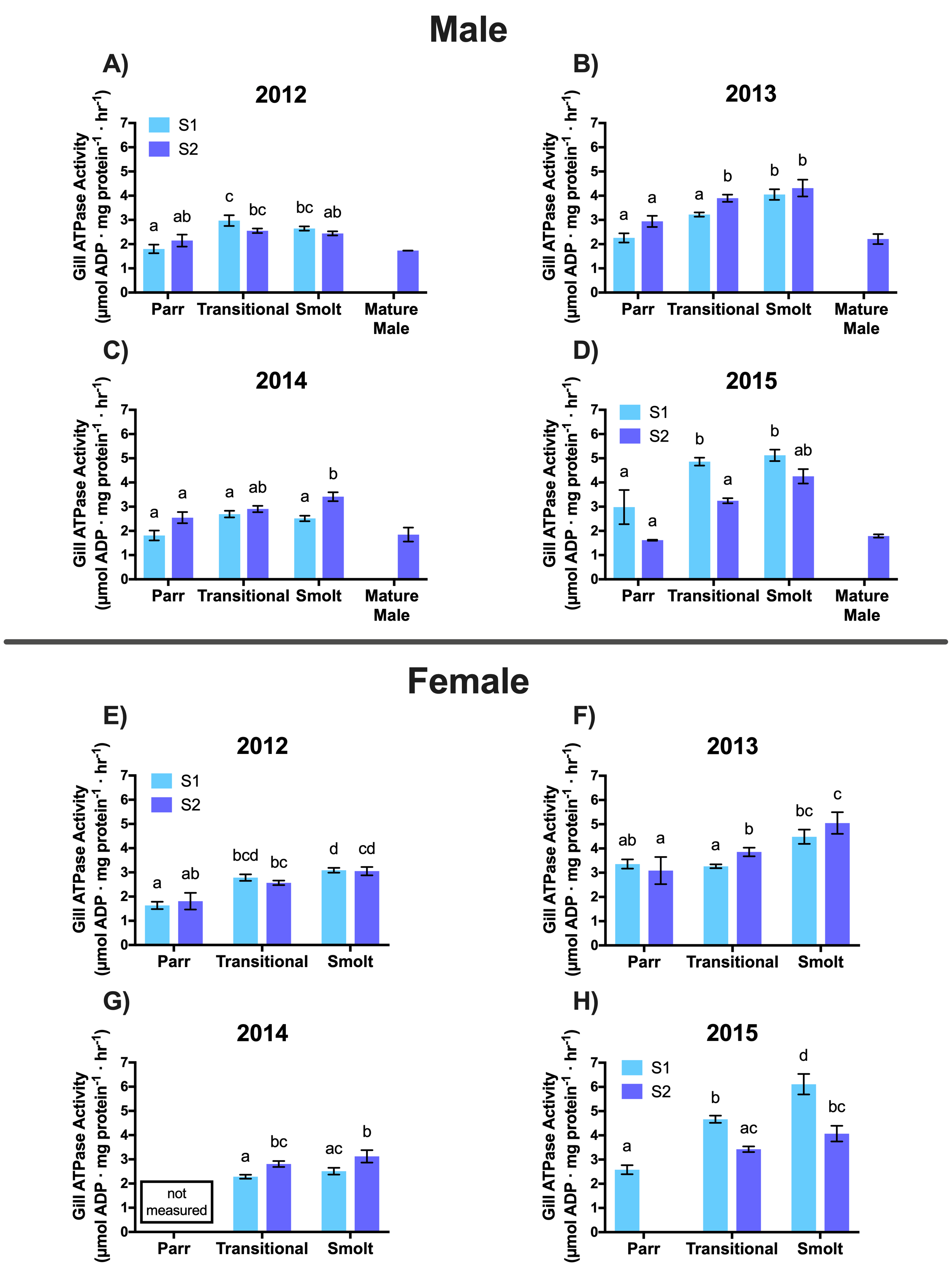

Supplement: S11 Fig — Gill Na+/K+ ATPase activity (μmol ADP · mg protein-1 · hr-1) of juvenile steelhead sampled at Winthrop National Fish Hatchery in release years 2012–2015 separated according to rearing treatment (S1 in light blue, S2 in violet) and visually determined qualitative smolt phenotype for males (A-D) and females (E-H). Data are mean ± SEM. Different letters indicate significant differences (p < 0.05) as determined by two-way ANOVA with Tukey’s post-hoc test. Mature males were not included in statistical analyses but are included on the graphs for visual reference. Graphs for pooled release years are contained in Fig 3. (TIFF) [file pone.0315016.s013.tiff]

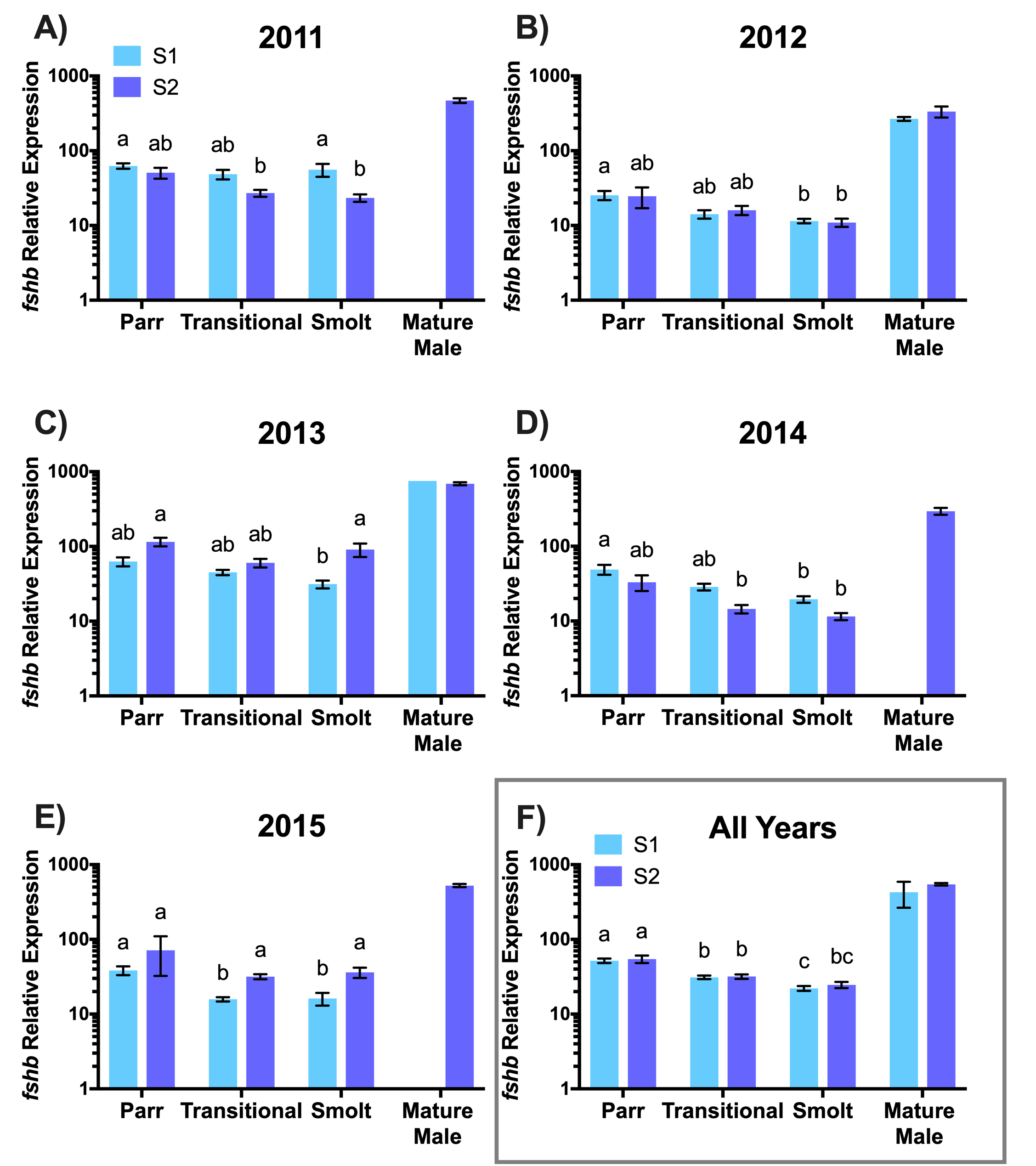

Supplement: S12 Fig — Pituitary follicle stimulating hormone beta-subunit (fshb) mRNA relative expression of juvenile male steelhead sampled at Winthrop National Fish Hatchery in release years 2011–2015 separated according to rearing treatment (S1 in light blue, S2 in violet) and visually determined qualitative smolt phenotype. Data are mean ± SEM. Graph in gray box (F) includes males combined across all release years. Different letters indicate significant differences (p < 0.05) as determined by two-way ANOVA with Tukey’s post-hoc test. Mature males were not included in statistical analyses but are included on the graphs for visual reference. (TIFF) [file pone.0315016.s014.tiff]

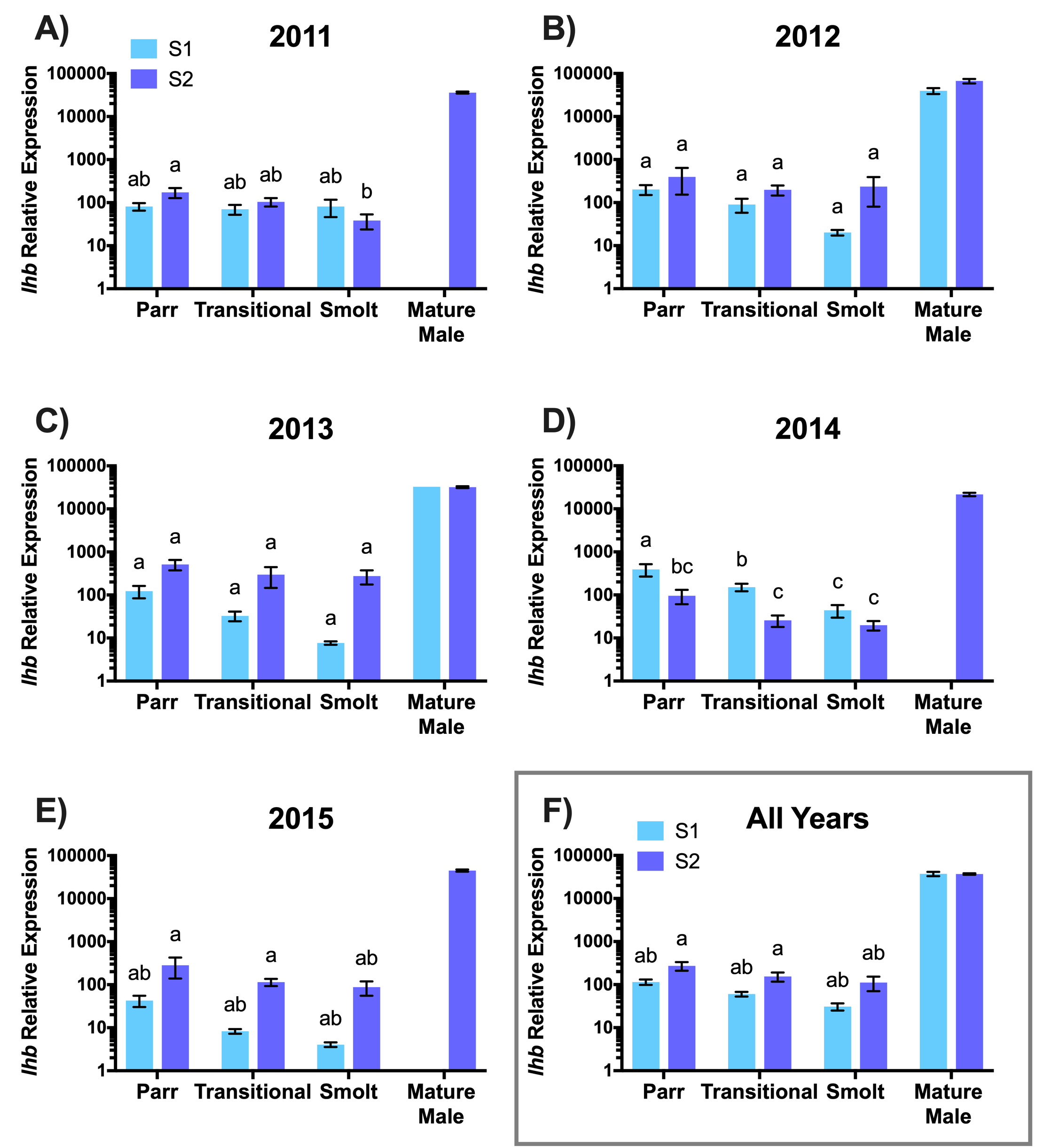

Supplement: S13 Fig — Pituitary luteinizing hormone beta-subunit (lhb) mRNA relative expression of juvenile male steelhead sampled at Winthrop National Fish Hatchery in release years 2011–2015 separated according to rearing treatment (S1 in light blue, S2 in violet) and visually determined qualitative smolt phenotype. Data are mean ± SEM. Graph in gray box (F) includes males combined across all release years. Different letters indicate significant differences (p < 0.05) as determined by two-way ANOVA with Tukey’s post-hoc test. Mature males were not included in statistical analyses but are included on the graphs for visual reference. (TIFF) [file pone.0315016.s015.tiff]

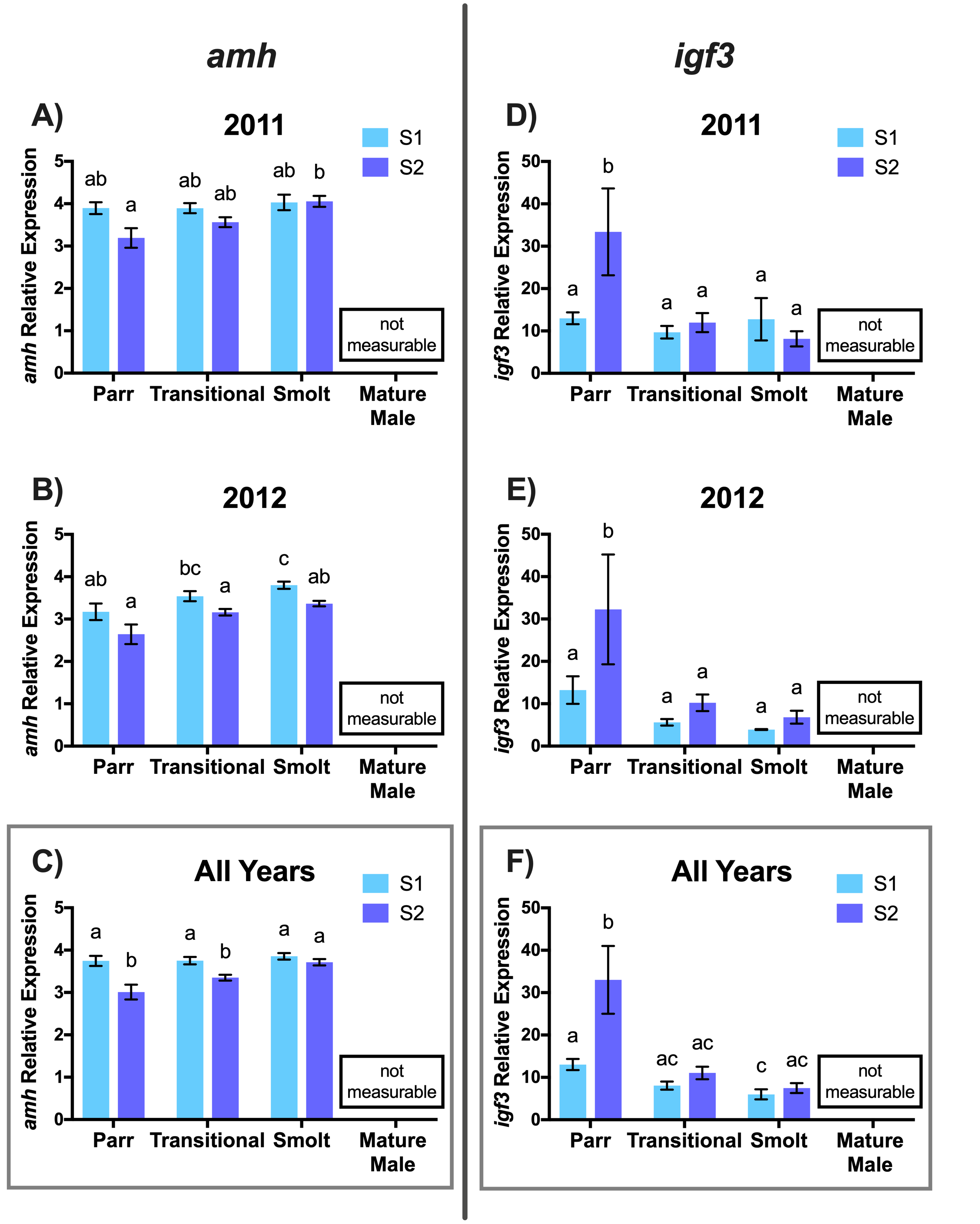

Supplement: S14 Fig — Testis anti-Mullerian hormone (amh; A-C) and insulin-like growth factor-3 (igf3; D-F) mRNA relative expression of juvenile male steelhead sampled at Winthrop National Fish Hatchery in release years 2011 and 2012 separated according to rearing treatment (S1 in light blue, S2 in violet) and visually determined qualitative smolt phenotype. Data are mean ± SEM. Graphs in gray boxes (C and F) include males combined across all release years. Different letters indicate significant differences (p < 0.05) as determined by two-way ANOVA with Tukey’s post-hoc test. Measurement of mature (spermiating) males is not possible with our method. Due to logistical constraints, we were unable to measure testis amh and igf3 in all years of the study. (TIFF) [file pone.0315016.s016.tiff]

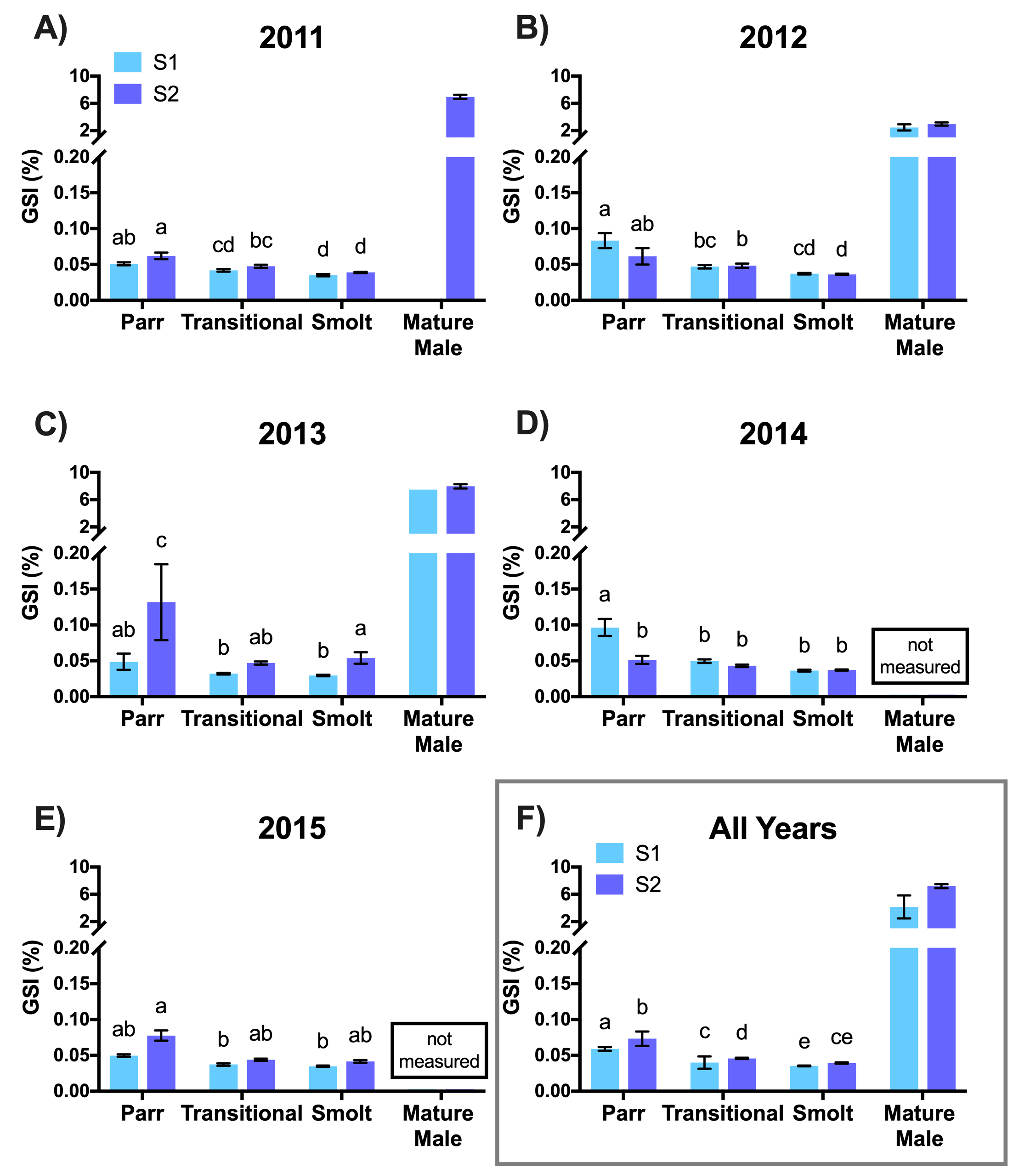

Supplement: S15 Fig — Gonadosomatic index (GSI; %) of juvenile male steelhead sampled at Winthrop National Fish Hatchery in release years 2011–2015 separated according to rearing treatment (S1 in light blue, S2 in violet) and visually determined qualitative smolt phenotype. Data are mean ± SEM. Graph in gray box (F) includes males combined across all release years. Different letters indicate significant differences (p < 0.05) as determined by two-way ANOVA with Tukey’s post-hoc test. Mature males were not included in statistical analyses but are included on the graphs for visual reference. In release years 2014 and 2015, mature male testes were not weighed so no data is shown. (TIFF) [file pone.0315016.s017.tiff]

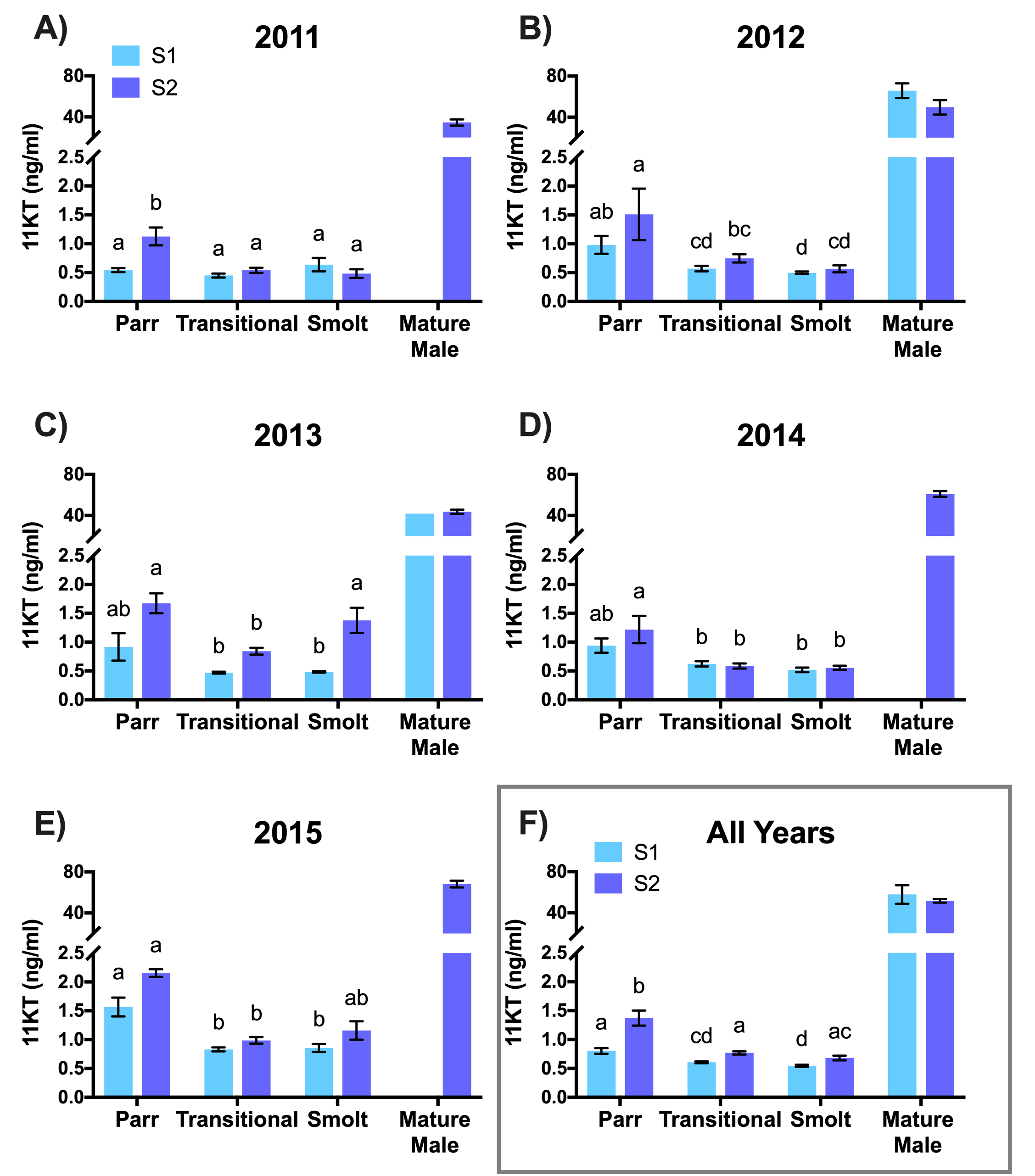

Supplement: S16 Fig — Plasma 11-ketotestosterone (11KT; ng/ml) of juvenile male steelhead sampled at Winthrop National Fish Hatchery in release years 2011–2015 separated according to rearing treatment (S1 in light blue, S2 in violet) and visually determined qualitative smolt phenotype. Data are mean ± SEM. Graph in gray box (F) includes males combined across all release years. Different letters indicate significant differences (p < 0.05) as determined by two-way ANOVA with Tukey’s post-hoc test. Mature males were not included in statistical analyses but are included on the graphs for visual reference. (TIFF) [file pone.0315016.s018.tiff]

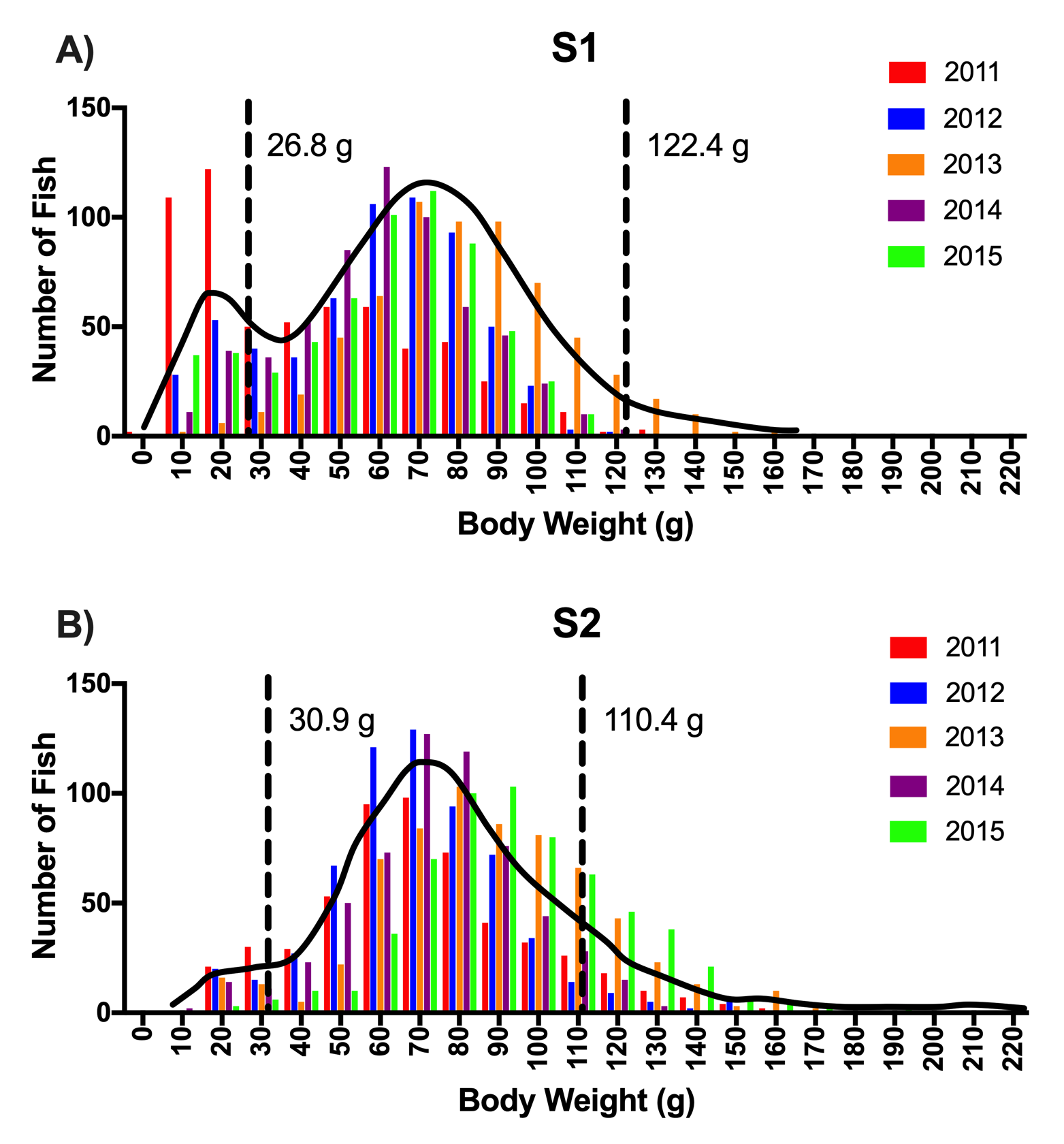

Supplement: S17 Fig — Frequency distributions of S1 (A) and S2 (B) body weight (g) for juvenile steelhead sampled at Winthrop National Fish Hatchery in release years 2011–2015, interleaved colored bars represent individual release years. Solid black lines indicate the density distribution for pooled release years. Dashed black reference lines indicate mode intersection as determined by finite mixture model analysis; however, these values were not used to categorize maturation status or residualism. In release year 2015, there is a single fish that is not represented graphically for the S2 treatment because it is large (in the 260 g bin) and accommodating its presence would reduce the spread of all other data. (TIFF) [file pone.0315016.s019.tiff]

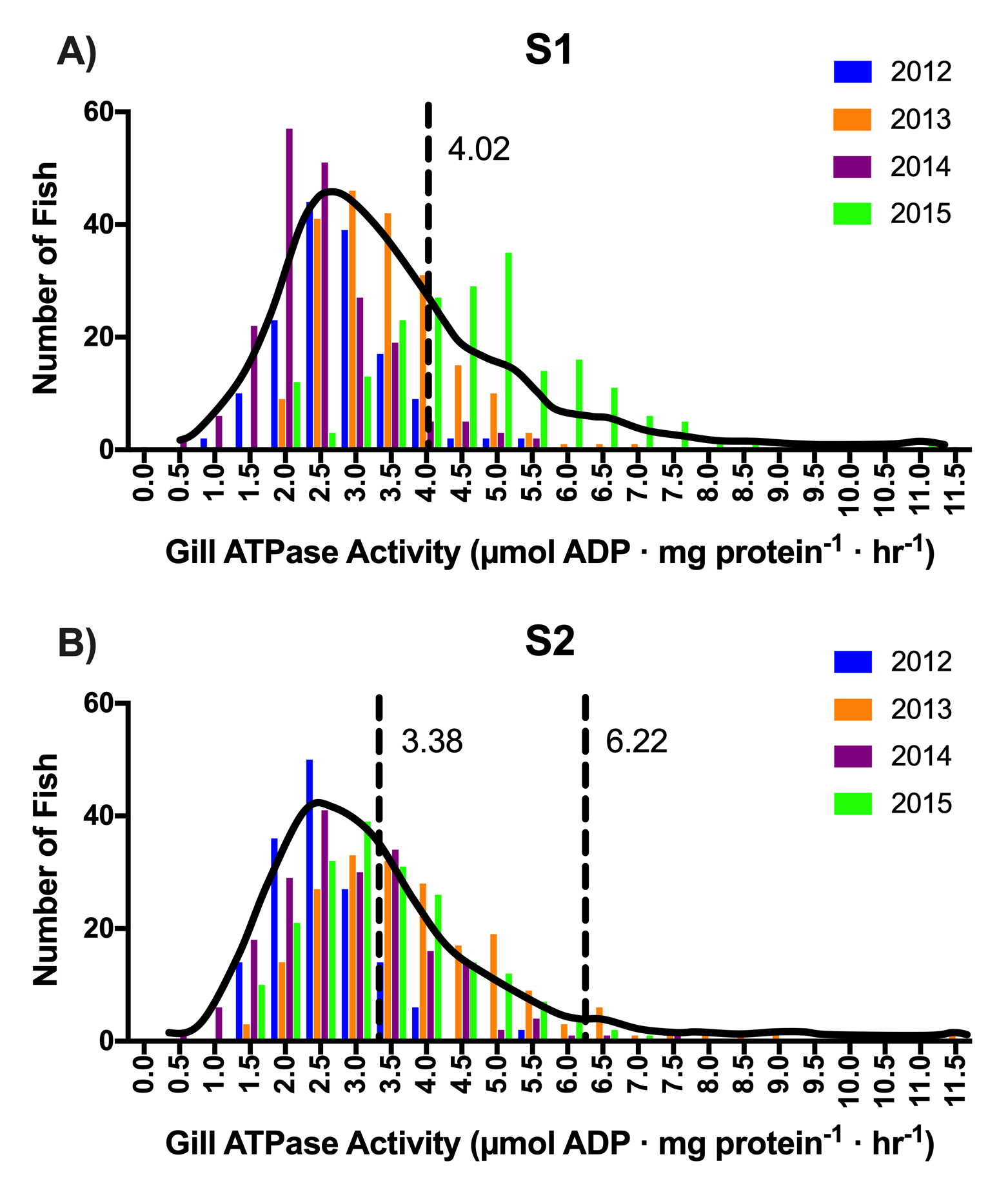

Supplement: S18 Fig — Frequency distributions of S1 (A) and S2 (B) gill Na+/K+ ATPase activity (μmol ADP · mg protein-1 · hr-1) for juvenile steelhead sampled at Winthrop National Fish Hatchery in release years 2012–2015, interleaved colored bars represent individual release years. Solid black lines indicate the density distribution for pooled release years. Dashed black reference lines indicate mode intersection as determined by finite mixture model analysis; however, these values were not used to categorize maturation status or residualism. (TIFF) [file pone.0315016.s020.tiff]

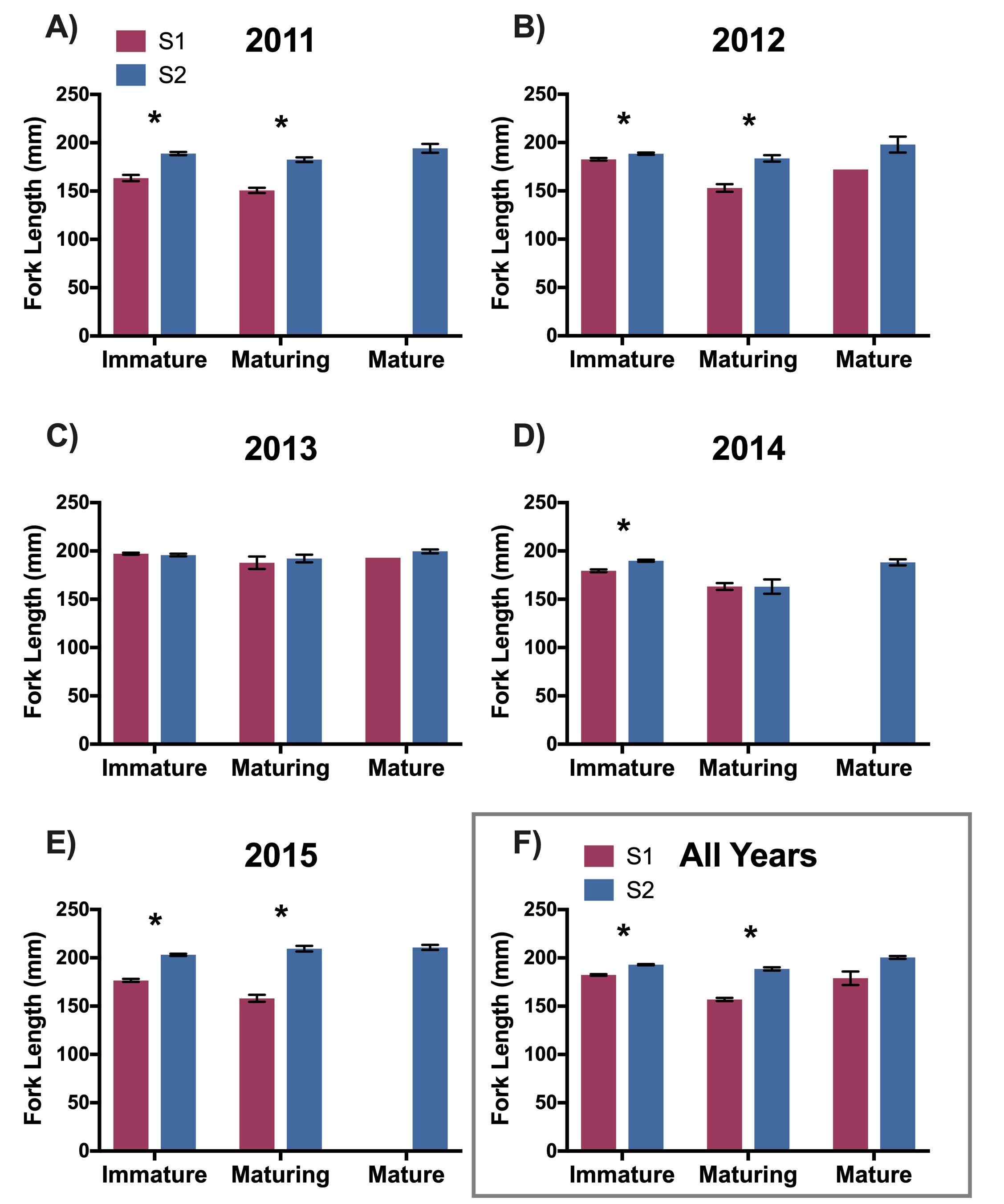

Supplement: S19 Fig — Fork length (mm) of juvenile male steelhead sampled at Winthrop National Fish Hatchery in release years 2011–2015 separated according to rearing treatment (S1 in red, S2 in blue) and maturation status as determined by finite mixture model analysis. Data are mean ± SEM. Graph in gray box (F) includes males combined across all release years. An asterisk indicates a significant difference (p < 0.05) between rearing treatments within a maturation category as determined by two-sample t-test. Mature males were not included in statistical analyses but are included on the graphs for visual reference. (TIFF) [file pone.0315016.s021.tiff]

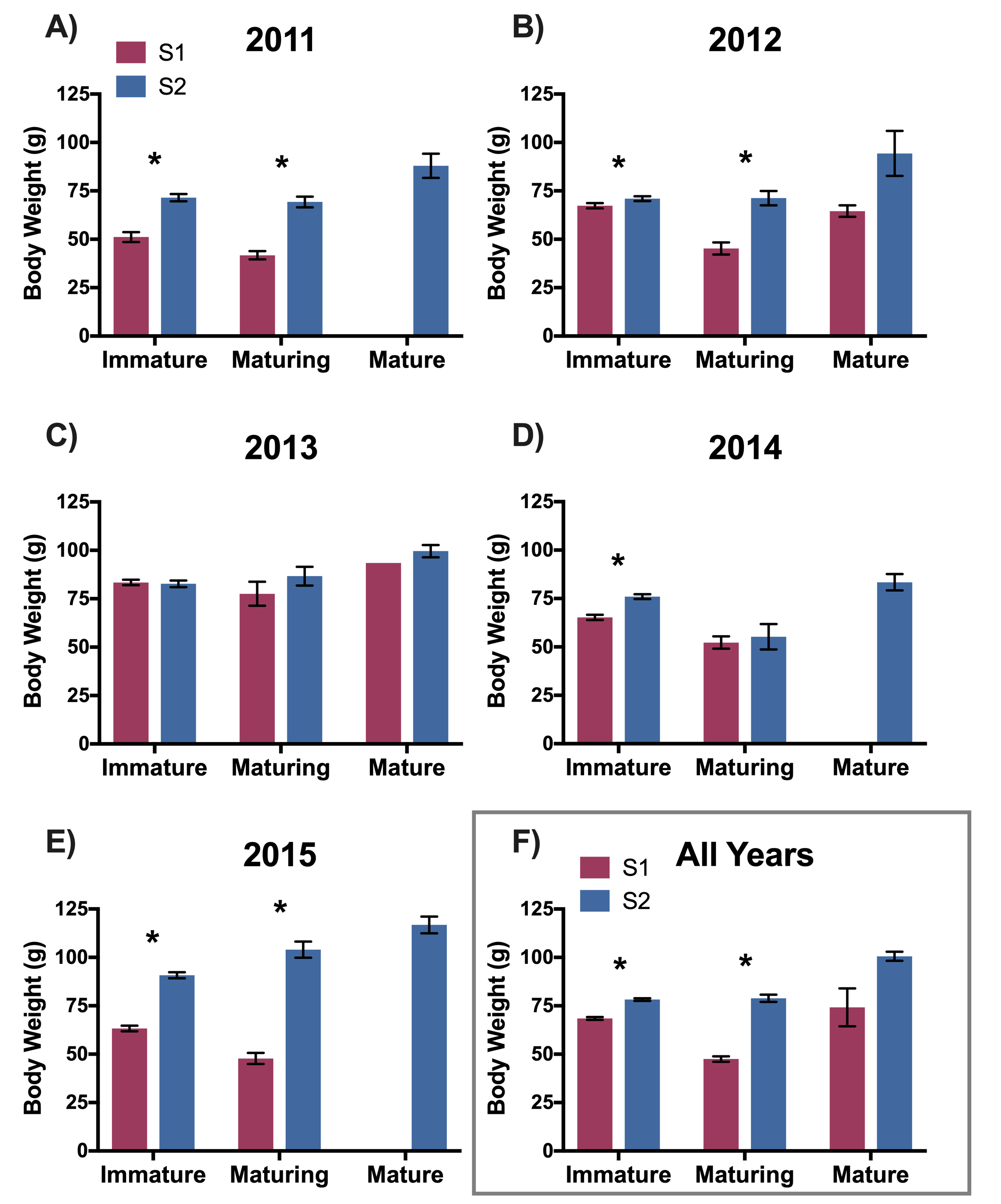

Supplement: S20 Fig — Body weight (g) of juvenile male steelhead sampled at Winthrop National Fish Hatchery in release years 2011–2015 separated according to rearing treatment (S1 in red, S2 in blue) and maturation status as determined by finite mixture model analysis. Data are mean ± SEM. Graph in gray box (F) includes males combined across all release years. An asterisk indicates a significant difference (p < 0.05) between rearing treatments within a maturation category as determined by two-sample t-test. Mature males were not included in statistical analyses but are included on the graphs for visual reference. (TIFF) [file pone.0315016.s022.tiff]

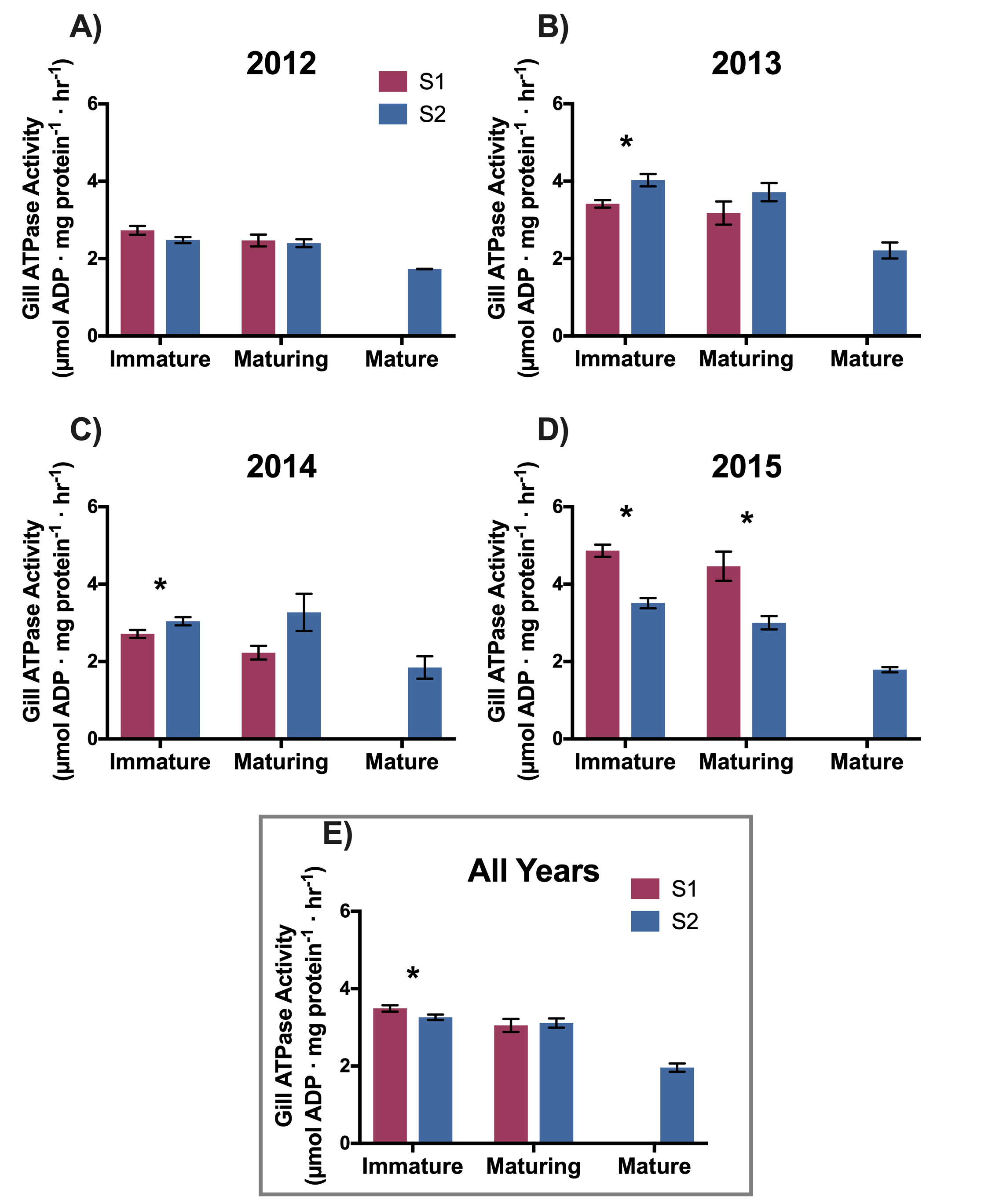

Supplement: S21 Fig — Gill Na+/K+ ATPase activity (μmol ADP · mg protein-1 · hr-1) of juvenile male steelhead sampled at Winthrop National Fish Hatchery in release years 2012–2015 separated according to rearing treatment (S1 in red, S2 in blue) and maturation status as determined by finite mixture model analysis. Data are mean ± SEM. Graph in gray box (E) includes males combined across all release years. An asterisk indicates a significant difference (p < 0.05) between rearing treatments within a maturation category as determined by two-sample t-test. Mature males were not included in statistical analyses but are included on the graphs for visual reference. (TIFF) [file pone.0315016.s023.tiff]

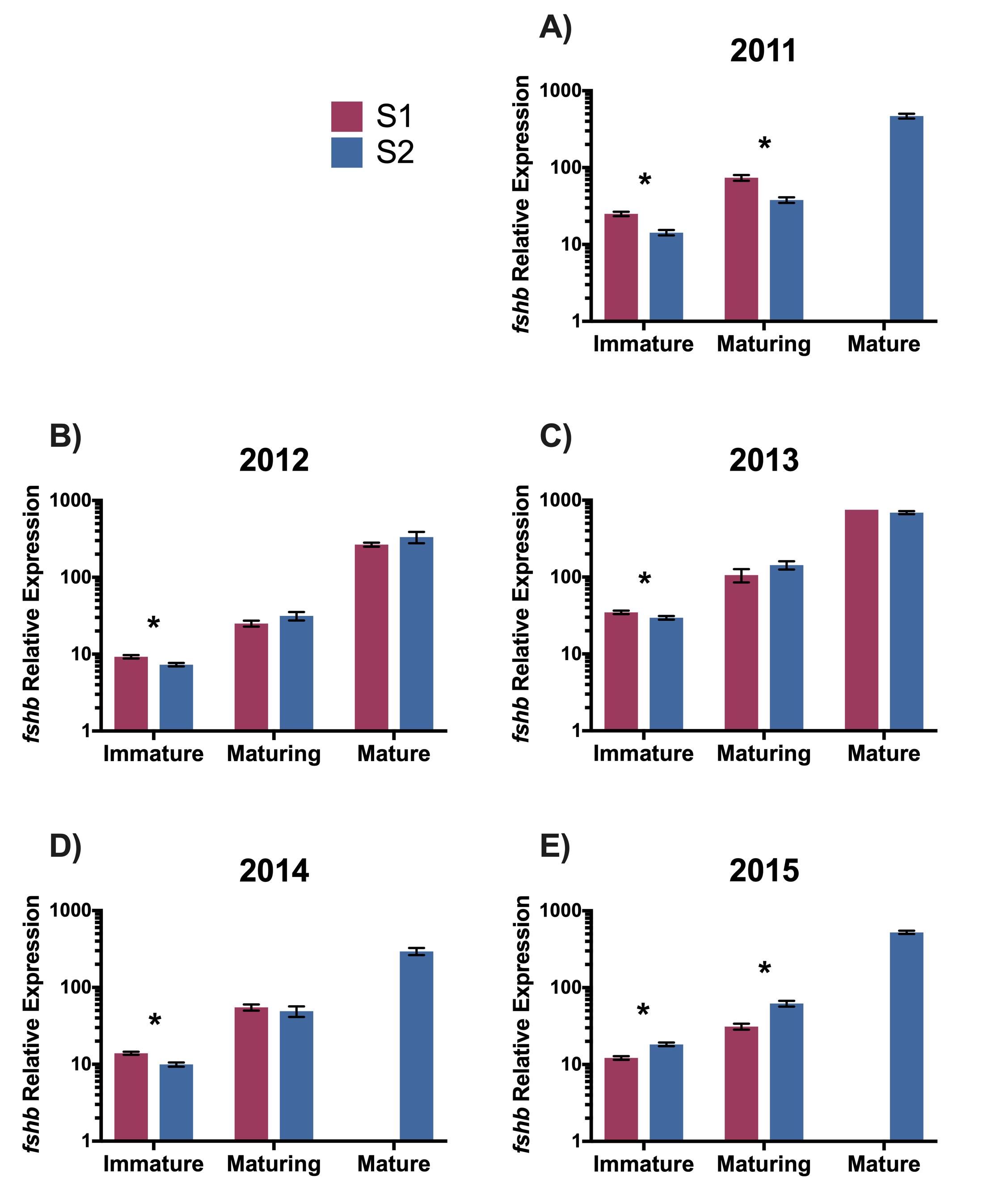

Supplement: S22 Fig — Pituitary follicle stimulating hormone beta-subunit (fshb) mRNA relative expression of juvenile male steelhead sampled at Winthrop National Fish Hatchery in release years 2011–2015 separated according to rearing treatment (S1 in red, S2 in blue) and maturation status as determined by finite mixture model analysis. Data are mean ± SEM. An asterisk indicates a significant difference (p < 0.05) between rearing treatments within a maturation category as determined by two-sample t-test. Mature males were not included in statistical analyses but are included on the graphs for visual reference. Graph for pooled release years is contained in Fig 9. (TIFF) [file pone.0315016.s024.tiff]

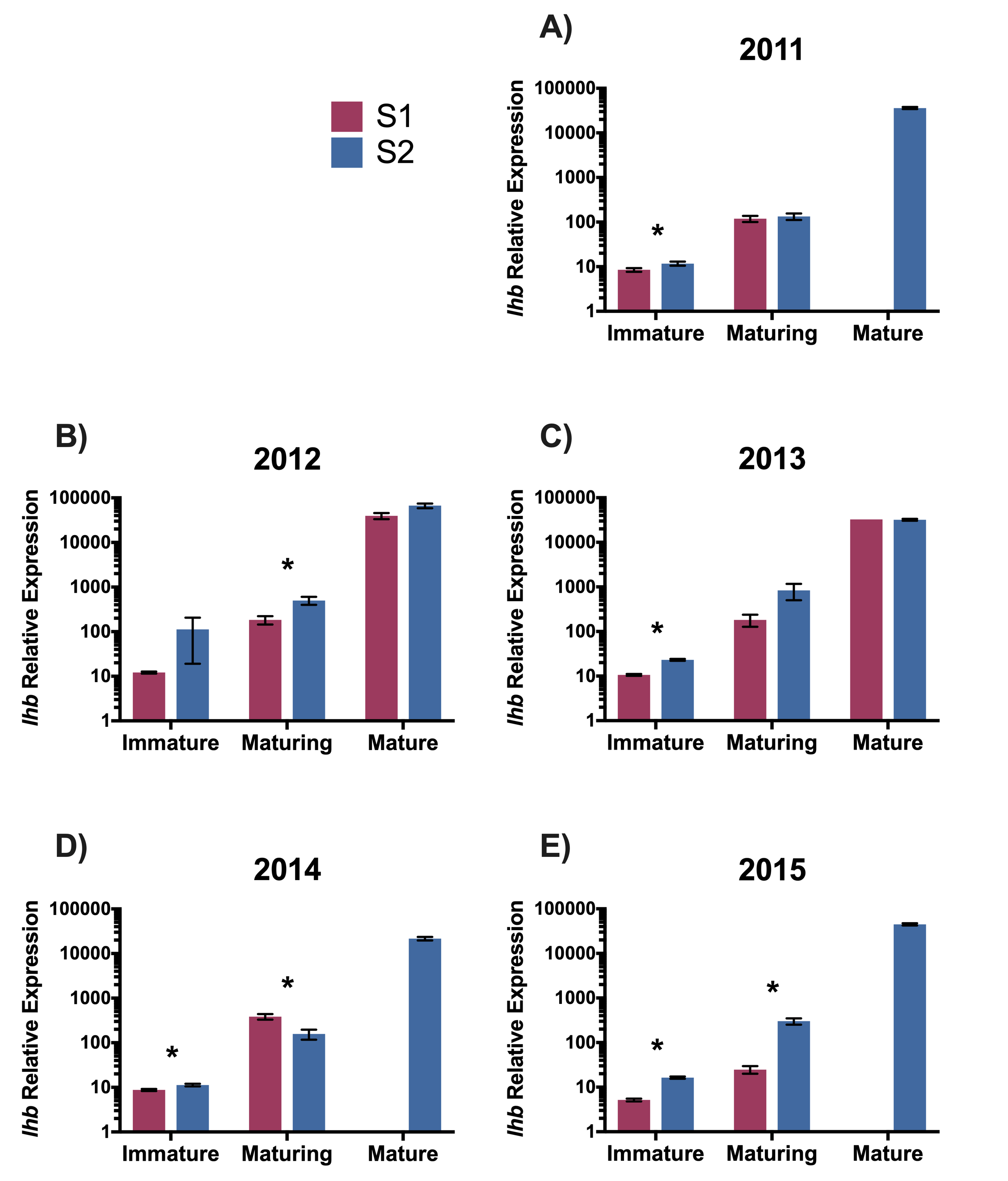

Supplement: S23 Fig — Pituitary luteinizing hormone beta-subunit (lhb) mRNA relative expression of juvenile male steelhead sampled at Winthrop National Fish Hatchery in release years 2011–2015 separated according to rearing treatment (S1 in red, S2 in blue) and maturation status as determined by finite mixture model analysis. Data are mean ± SEM. An asterisk indicates a significant difference (p < 0.05) between rearing treatments within a maturation category as determined by two-sample t-test. Mature males were not included in statistical analyses but are included on the graphs for visual reference. Graph for pooled release years is contained in Fig 9. (TIFF) [file pone.0315016.s025.tiff]

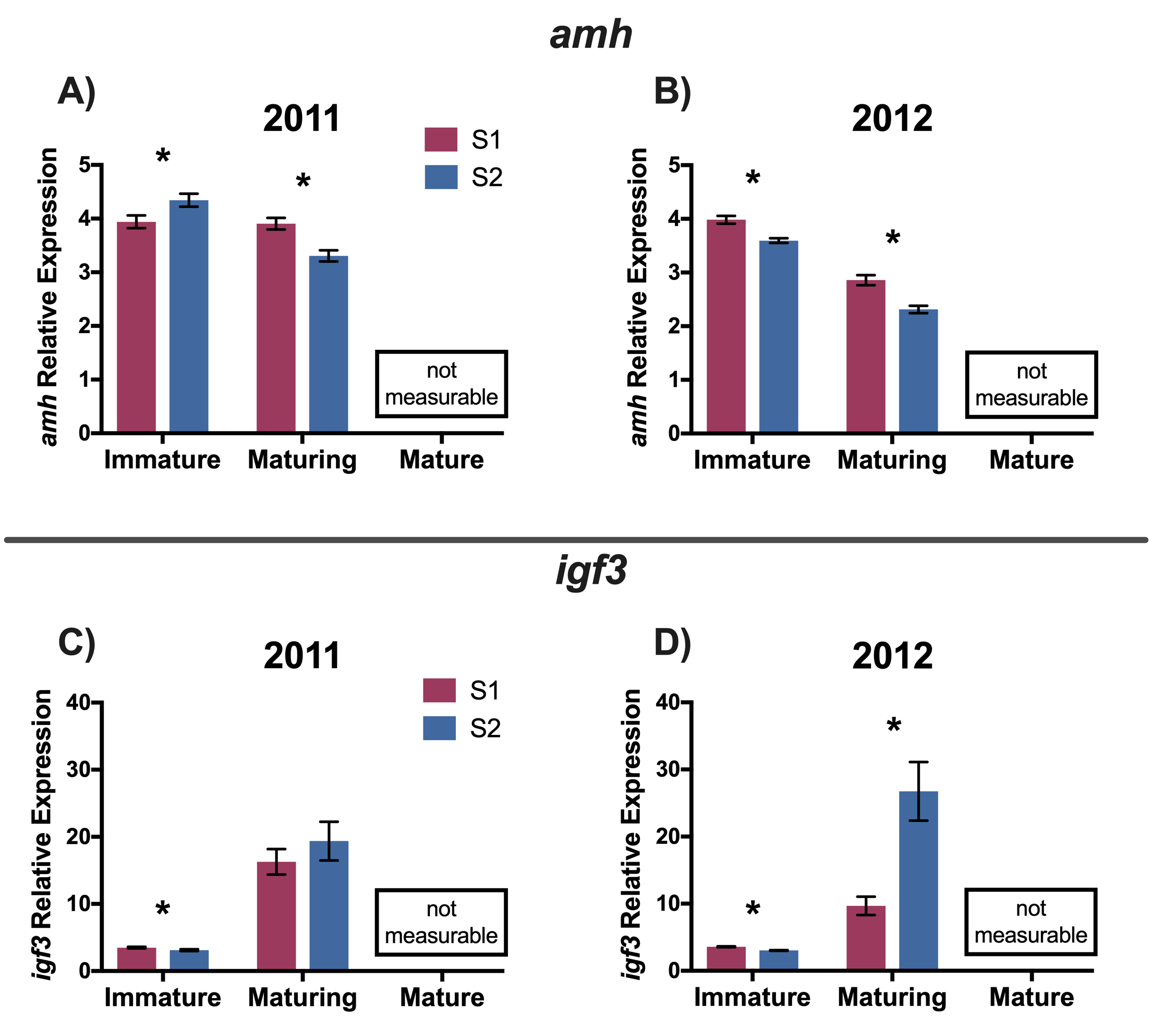

Supplement: S24 Fig — Testis anti-Mullerian hormone (amh; A-B) and insulin-like growth factor-3 (igf3; C-D) mRNA relative expression of juvenile male steelhead sampled at Winthrop National Fish Hatchery in release years 2011 and 2012 separated according to rearing treatment (S1 in red, S2 in blue) and maturation status as determined by finite mixture model analysis. Data are mean ± SEM. An asterisk indicates a significant difference (p < 0.05) between rearing treatments within a maturation category as determined by two-sample t-test. Measurement of mature (spermiating) males is not possible with our method. Due to logistical constraints, we were unable to measure testis amh and igf3 in all years of the study. Graphs for pooled release years are contained in Fig 9. (TIFF) [file pone.0315016.s026.tiff]

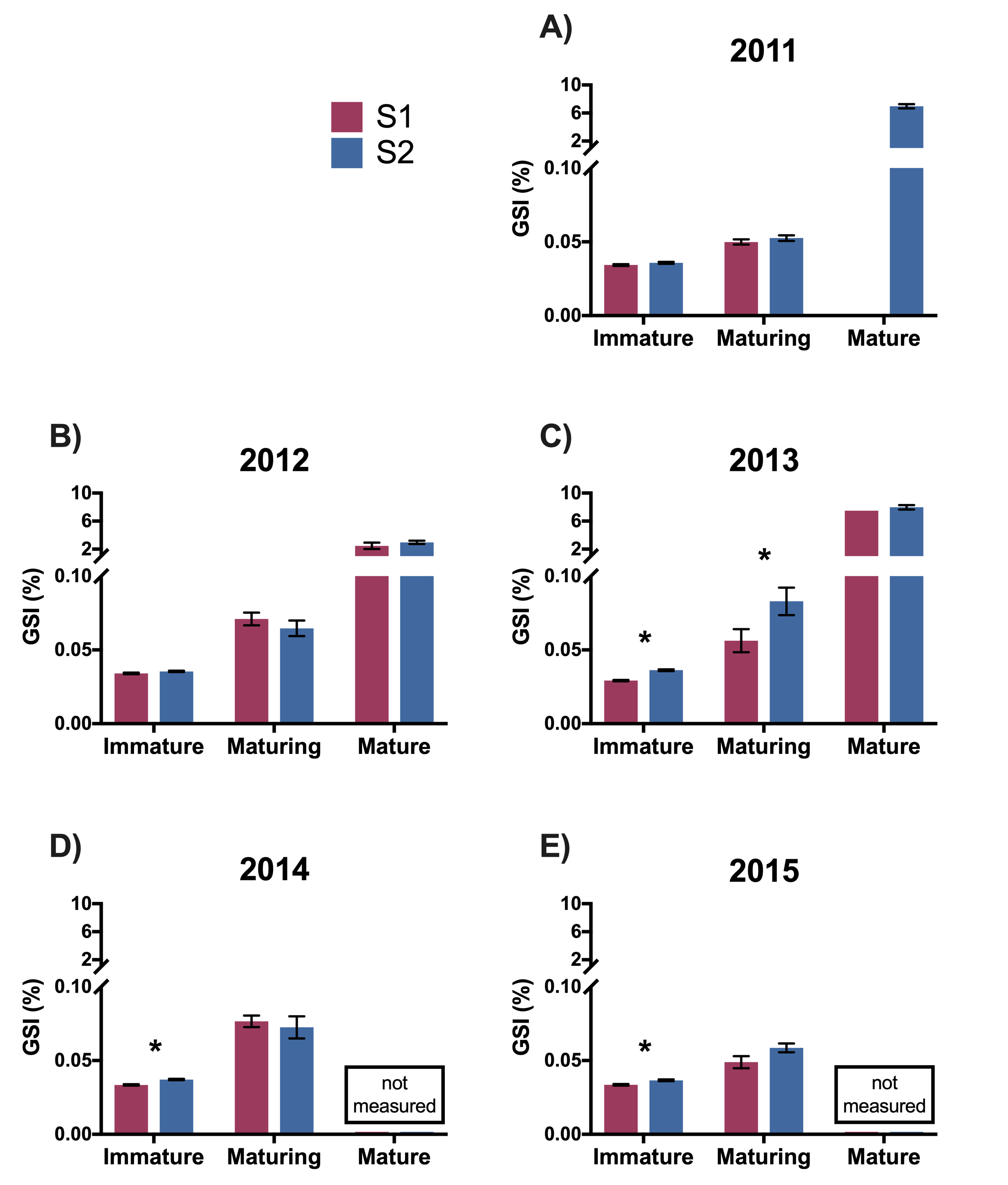

Supplement: S25 Fig — Gonadosomatic index (GSI; %) of juvenile male steelhead sampled at Winthrop National Fish Hatchery in release years 2011–2015 separated according to rearing treatment (S1 in red, S2 in blue) and maturation status as determined by finite mixture model analysis. Data are mean ± SEM. An asterisk indicates a significant difference (p < 0.05) between rearing treatments within a maturation category as determined by two-sample t-test. Mature males were not included in statistical analyses but are included on the graphs for visual reference. In release years 2014 and 2015, mature male testes were not weighed so no data is shown. Graph for pooled release years is contained in Fig 9. (TIFF) [file pone.0315016.s027.tiff]

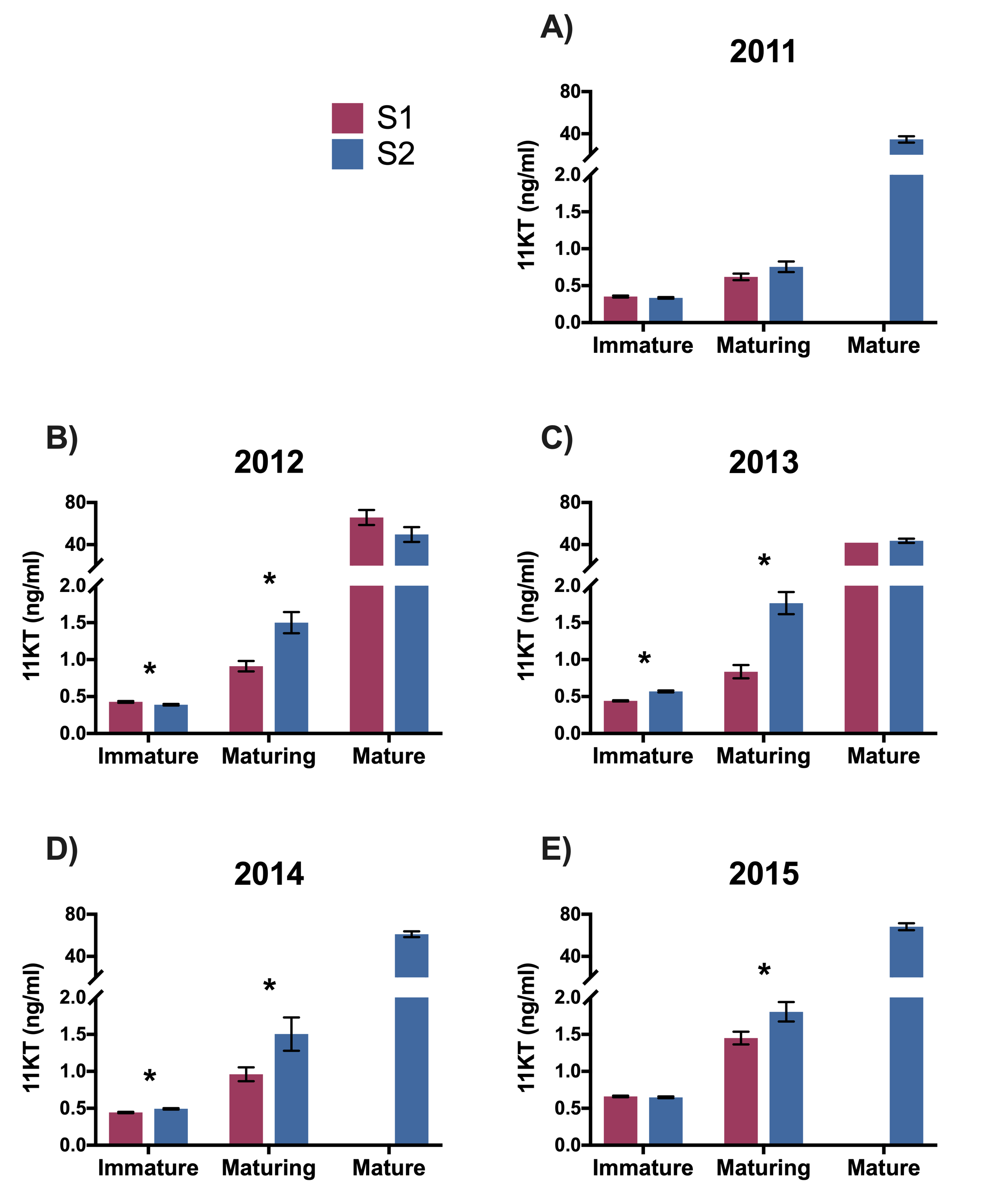

Supplement: S26 Fig — Plasma 11-ketotestosterone (11KT; ng/ml) of juvenile male steelhead sampled at Winthrop National Fish Hatchery in release years 2011–2015 separated according to rearing treatment (S1 in red, S2 in blue) and maturation status as determined by finite mixture model analysis. Data are mean ± SEM. An asterisk indicates a significant difference (p < 0.05) between rearing treatments within a maturation category as determined by two-sample t-test. Mature males were not included in statistical analyses but are included on the graphs for visual reference. Graph for pooled release years is contained in Fig 9. (TIFF) [file pone.0315016.s028.tiff]
